# Cerebral Oximetry in Extremely Preterm Infants: 2-Year Follow-Up of the SafeBoosC-III Randomized Clinical Trial

**DOI:** 10.1001/jamapediatrics.2026.1066

**Published:** 2026-04-20

**Authors:** Marie Isabel Skov Rasmussen, Mathias L. Hansen, Adelina Pellicer, Simon Hyttel-Sørensen, Ebru Ergenekon, Tomasz Szczapa, Cornelia Hagmann, Gunnar Naulaers, Jonathan Mintzer, Monica Fumagalli, Gabriel Dimitriou, Eugene Dempsey, Jakub Tkaczyk, Siv Fredly, Anne M. Heuchan, Gerhard Pichler, Hans Fuchs, Saudamini Nesargi, Gitte H. Hahn, Salvador Piris-Borregas, Jan Širc, Miguel Alsina-Casanova, Martin Stocker, Hilal Ozkan, Kosmas Sarafidis, Nicole J. Kraus, Tanja Karen, Beata Rzepecka-Weglarz, Serife S. Oguz, Liesbeth Thewissen, Luis Arruza, Asli C. Memisoglu, Ruth del Rio Florentino, Mariana Baserga, Pierre Maton, Juliane Schneider, M. Isabel de las Cuevas, Sofie Sommer Hedegaard, Pamela Zafra, Lars Bender, Sarah Farquharson, Agnieszka Ochoda-Mazur, Chantal Lecart, Afif El-Khuffash, Caitríona Ní Chathasaigh, Jan Miletin, Evangelia Papathoma, Zachary Vesoulis, Francesca Serrao, and the SafeBoosC-III Follow-Up Writing Group for the SafeBoosC-III Follow-Up Collaborator Group, Luc Cornette, Beril Yasa, Anja Klamer, Francisca Barcos-Munoz, Tatiana Boetti, Merih Cetinkaya, Mahmoud Montasser, Eleftheria Hatzidaki, Renata Bokiniec, Sylwia Marciniak, Lina Chalak, Shashidhar A. Rao, Iwona Sadowska-Krawczenko, Itziar Serrano-Viñuales, Barbara Krolak-Olejnik, Anne Mette Plomgaard, Bo Mølholm Hansen, Markus Harboe Olsen, Christian Gluud, Janus C. Jakobsen, Gorm Greisen

**Affiliations:** 1 Department of Neonatology, Copenhagen University Hospital–Rigshospitalet, Copenhagen, Denmark; 2 Copenhagen Trial Unit, Centre for Clinical Intervention Research, The Capital Region, Copenhagen University Hospital–Rigshospitalet, Copenhagen, Denmark; 3 Department of Neonatology, La Paz University Hospital, Madrid, Spain; 4 Hospital La Paz Institute for Health Research-IdiPAZ, Madrid, Spain; 5 Department of Intensive Care, Copenhagen University Hospital–Rigshospitalet, Copenhagen, Denmark; 6 Division of Newborn Medicine, Gazi University Hospital, Ankara, Turkey; 7 2nd Department of Neonatology, Neonatal Biophysical Monitoring and Cardiopulmonary Therapies Research Unit, Chair of Neonatology, Poznan University of Medical Sciences, Poznan, Poland; 8 Pediatric Intensive Care and Neonatology, Children’s University Hospital of Zurich, Zurich, Switzerland; 9 Department of Development and Regeneration, KU Leuven, Leuven, Belgium; 10 Department of Pediatrics, Division of Newborn Medicine, Mountainside Medical Center, Montclair, New Jersey; 11 Fondazione IRCCS Ca’ Granda Ospedale Maggiore Policlinico, Milan, Italy; 12 Department of Clinical Sciences and Community Health, University of Milan, Milan, Italy; 13 NICU, Department of Pediatrics, Patras Medical School, Patras, Greece; 14 Infant Centre and Department of Paediatrics and Child Health, University College Cork, Cork, Ireland; 15 Department of Neonatology, University Hospital Motol, Prague, Czech Republic; 16 Department of Neonatology, Oslo University Hospital, Oslo, Norway; 17 Department of Neonatal Medicine, Royal Hospital for Children, Glasgow, United Kingdom; 18 Department of Pediatrics, Medical University of Graz, Graz, Austria; 19 Research Unit for Neonatal Micro- and Macrocirculation, Medical University of Graz, Graz, Austria; 20 Division of Neonatology and Pediatric Intensive Care Medicine, Center for Pediatrics and Adolescents Medicine, Medical Center - University of Freiburg, Freiburg, Germany; 21 Faculty of Medicine, University of Freiburg, Freiburg, Germany; 22 Department of Neonatology, St John’s Medical College, Bangalore, India; 23 Neonatology Department, 12 de Octubre University Hospital, Madrid, Spain; 24 The Institute for the Care of Mother and Child, Prague, Czech Republic; 25 Third Faculty of Medicine, Charles University, Prague, Czech Republic; 26 Department of Neonatology, Hospital Clínic Barcelona, BCNatal-Barcelona Center for Maternal-Fetal and Neonatal Medicine, Barcelona, Spain; 27 Intensive Care Unit, Children’s Hospital Lucerne, Lucerne, Switzerland; 28 Faculty of Medicine and Medical Science, University of Lucerne, Lucerne, Switzerland; 29 Department of Neonatology, Faculty of Medicine, Bursa Uludag University, Bursa, Turkey; 30 1st Department of Neonatology, Aristotle University of Thessaloniki, Ippokrateion General Hospital of Thessaloniki, Greece; 31 Division of Neonatology, Department of Pediatrics, Loma Linda University Children’s Hospital, Loma Linda, California; 32 Department of Neonatology, University Hospital Zurich, Zurich, Switzerland; 33 Department of Neonatology, Centrum Medyczne “Ujastek” Sp. z o.o., Krakow, Poland; 34 Department of Neonatology, NICU, University of Health Sciences, Ankara City Hospital, Ankara, Turkey; 35 Department of Neonatology, Hospital Clinico San Carlos–IdISSC, Madrid, Spain; 36 Division of Neonatology, Department of Pediatrics, Marmara University Research and Education Hospital, Marmara University, School of Medicine, Istanbul, Turkey; 37 Department of Neonatology, Hospital Sant Joan de Deu, Barcelona, Spain; 38 Division of Neonatology, University of Utah Hospital, Salt Lake City; 39 Service de Néonatologie, Clinique CHC Montlégia-Liège-Belgium, Belgium; 40 Clinic of Neonatology, Department of Women, Mother and Child, University Hospital Center, Vaud, Switzerland; 41 University of Lausanne, Vaud, Switzerland; 42 Neonatal Unit, Donostia University Hospital-IIS Biogipuzkoa, Basque Country, Spain; 43 Department of Pediatrics and Adolescent Medicine, Gødstrup Hospital, Denmark; 44 Neonatal Intensive Care Unit, Puerta del Mar University Hospital, Cádiz, Spain; 45 Department of Neonatology, Aalborg University Hospital, Aalborg, Denmark; 46 Neonatology Clinic, Jagiellonian University Medical College, Kraków, Poland; 47 Department of Neonatology, GHdC Charleroi, Belgium; 48 Department of Paediatrics, School of Medicine, Royal College of Surgeons in Ireland, Dublin, Ireland; 49 National Maternity Hospital, Dublin, Ireland; 50 The Coombe Hospital, Dublin, Ireland; 51 University College Dublin, Dublin, Ireland; 52 2nd Faculty of Medicine, Charles University, Prague, Czech Republic; 53 Neonatal Intensive Care Unit, Alexandra University and State Hospital, Athens, Greece; 54 Division of Newborn Medicine, Department of Pediatrics, Washington University School of Medicine in St Louis, St Louis, Missouri; 55 Unità Operativa Complessa di Neonatologia, Dipartimento Scienze della Salute della Donna, del Bambino e di Sanità Pubblica, Fondazione Policlinico Universitario A. Gemelli IRCCS, Rome, Italy; 56 Department of Neonatology, AZ St-Jan Bruges, Bruges, Belgium; 57 Basaksehir Cam and Sakura City Hospital, Basaksehir, Turkey; 58 Department of Neonatology, Kanuni Sultan Suleyman Training and Research Hospital, Küçükçekmece/İstanbul, Turkey; 59 Department of Pediatrics, Odense University Hospital, Odense, Denmark; 60 Division of Neonatology and Pediatric Intensive Care, Children’s University Hospital of Geneva and University of Geneva, Geneva, Switzerland; 61 SC Neonatologia, Osp. S.Anna - Città della Salute e della Scienza di Torino, Turin, Italy; 62 Neonatology Department, University Hospital Wishaw, Wishaw, Scotland, United Kingdom; 63 Department of Neonatology & NICU, University Hospital of Heraklion, Crete, Greece; 64 Department of Neonatology and Neonatal Intensive Care, Medical University of Warsaw, Warsaw, Poland; 65 Neonatal Unit, Specialist Hospital No. 2, Bytom, Poland; 66 Division of Pediatrics–Neonatal-Perinatal, UT Southwestern, Dallas, Texas; 67 Department of Neonatology, Collegium Medicum in Bydgoszcz Nicolaus Copernicus University in Torun, Bydgoszcz, Poland; 68 Neonatology Division, Miguel Servet University Hospital, Zaragoza, Spain; 69 Department of Neonatology, Wroclaw Medical University, Wroclaw, Poland; 70 Department of Pediatrics, Hvidovre University Hospital, Hvidovre, Denmark; 71 Department of Paediatrics and Adolescent Medicine, Copenhagen University Hospital, Hilleroed, Denmark; 72 Department of Neuroanaesthesiology, Neuroscience Centre, Copenhagen University Hospital – Rigshospitalet, Copenhagen, Denmark; 73 Department of Regional Health Research, The Faculty of Health Sciences, University of Southern Denmark, Odense, Denmark

## Abstract

**Importance:**

Cerebral oximetry monitoring in the first 72 hours after birth has not been shown to reduce death or severe brain injury at 36 weeks’ postmenstrual age in extremely preterm infants. The long-term effects remain uncertain.

**Objective:**

To determine whether treatment guided by cerebral oximetry monitoring during the first 72 hours after birth reduces the risk of death or longer-term neurodevelopmental outcomes at 2 years’ corrected age, compared with usual care.

**Design, Setting, and Participants:**

In the phase 3 Safeguarding the Brain of Our Smallest Children (SafeBoosC-III) randomized clinical trial, we compared treatment guided by cerebral oximetry monitoring with usual care for the first 72 hours after birth. Seventy sites across 17 countries randomized 1601 infants within 6 hours of birth. Infants from 56 sites participated in this follow-up. Blinded assessors evaluated outcomes using a predefined 3-tier data model combining formal clinical assessments, parental questionnaires, and informal assessments. Data were analyzed from October to December 2024.

**Interventions:**

Treatment guided by cerebral oximetry monitoring for the first 72 hours after birth vs usual care.

**Main Outcomes and Measures:**

The coprimary outcomes were as follows: (1) death or moderate or severe neurodevelopmental disability and (2) Bayley cognitive composite score, both assessed at approximately 2 years’ corrected age.

**Results:**

A total of 1438 infants (mean [SD] age, 26.0 [1.3] weeks; 758 male [52.7%]) participated in this follow-up study. Participants were followed up from October 2021 to October 2024. Death or moderate or severe neurodevelopmental disability occurred in 292 of 620 infants (47.1%) in the cerebral oximetry group compared with 321 of 669 infants (48.0%) in the usual-care group (relative risk with cerebral oximetry, 0.96; 97.5% CI, 0.85-1.07; *P* = .45). The mean (SD) Bayley cognitive score was 92.8 (17.0) in the cerebral oximetry group compared with 93.2 (17.3) in the usual-care group (mean difference with cerebral oximetry, −0.14; 97.5% CI, −3.24 to 2.96; *P* = .92).

**Conclusions and Relevance:**

In extremely preterm infants, treatment guided by cerebral oximetry monitoring compared with usual care for the first 72 hours after birth did not result in a lower incidence of death or moderate or severe neurodevelopmental disability nor higher Bayley cognitive scores at 2 years’ corrected age. The routine use of cerebral oximetry monitoring during the first 72 hours after birth in extremely preterm infants to reduce neurodevelopmental disability was not supported by this trial.

**Trial Registration:**

ClinicalTrials.gov Identifier: NCT05134116

## Key Points

**Question:**

Does treatment guided by cerebral oximetry monitoring during the first 72 hours after birth reduce the risk of death or moderate or severe neurodevelopmental disability and cognitive impairment at 2 years of corrected age in extremely preterm infants?

**Findings:**

In this 2-year follow-up of the Safeguarding the Brain of Our Smallest Children (SafeBoosC-III) randomized clinical trial including 1438 infants, death or moderate or severe neurodevelopmental disability did not differ between the cerebral oximetry group and the usual-care group, and mean Bayley cognitive scores at 2 years did not differ significantly between groups.

**Meaning:**

Results reveal that the routine use of cerebral oximetry monitoring during the first 72 hours after birth in extremely preterm infants to reduce death or moderate or severe neurodevelopmental disability and cognitive impairment was not supported by this trial.

## Teaser

This randomized clinical trial investigates if treatment guided by cerebral oximetry monitoring during the first 72 hours after birth reduces the risk of death or moderate or severe neurodevelopmental disability and cognitive impairment at 2 years of corrected age in extremely preterm infants.

## Introduction

Despite advancements in the care of extremely preterm infants, mortality rates remain around 20%, and up to 25% of survivors experience substantial neurodevelopmental disabilities.^1^ These include cerebral palsy, cognitive and neurosensory deficits, and other conditions affecting the daily life of both children and their families.^2^ The risk of brain injury is particularly high during the earliest postnatal days due to immature respiratory and circulatory systems as well as impaired autoregulation of the cerebral blood flow.^3^ This hemodynamic instability can lead to episodes of cerebral hypoxia,^4^ which are associated with an increased risk of death, intracranial hemorrhage, ischemic lesions, and later neurodevelopmental disabilities.^5^ Cerebral oximetry monitoring may detect cerebral hypoxia, enabling clinicians to adjust cardiorespiratory support accordingly.^6^ The Safeguarding the Brain of Our Smallest Children (SafeBoosC-III) trial randomized 1601 extremely preterm infants across 70 sites in 17 countries and assessed whether treatment guided by cerebral oximetry monitoring during the first 72 hours after birth could reduce these risks.^7^ The trial found no differences in the composite primary outcome of death or severe brain injury detected on routine cerebral ultrasound scans at 36 weeks’ postmenstrual age compared with usual care. However, the relationship between neonatal brain injury and long-term neurodevelopmental disability is not simple.^8^ The correlation is strong for the most severe brain injuries; while ultrasound scans can detect these conditions, their limited predictive capacity highlights the need for assessing long-term outcomes in neonatal trials.^9^ To address these patient-relevant outcomes, we conducted a 2-year follow-up of the SafeBoosC-III trial to evaluate whether treatment guided by cerebral oximetry monitoring improves survival and neurodevelopmental outcomes compared with usual care.

## Methods

### Trial Design

The SafeBoosC-III follow-up is an investigator-initiated, pragmatic, multinational follow-up study of the participants in a phase 3 randomized clinical trial.^7^ The study protocol and statistical analysis plan were published before data analysis (Supplement 1 and Supplement 2, respectively).^10,11^ The protocol received approval from the ethics committees at each participating site, ensuring adherence to regulatory standards, and a list of investigators is available in the eAppendix in Supplement 3. Prior publications have documented details on randomization, blinding and interventions.^7,12,13^ The SafeBoosC-III follow-up was not prespecified in the original SafeBoosC-III trial and was mentioned as a potential study in the protocol appendix. The follow-up study was deemed feasible to conduct and was initiated 6 months after randomization started when sufficient recruitment and site participation had been achieved. Data were collected from October 2021 to October 2024. This study is reported following the Consolidated Standards of Reporting Trials (CONSORT) reporting guidelines.

### Participants

Of the 70 sites that randomized infants in the SafeBoosC-III trial, 56 participated in this follow-up study. Of the 14 nonparticipating sites, 2 withdrew and 12 were excluded due to lack of progress (eMethods in Supplement 3). No follow-up data were collected from the excluded sites. Participant race and ethnicity data were not gathered. Eligibility criteria were enrollment in the SafeBoosC-III trial (gestational age at birth less than 28 weeks, decision to provide full life support, possibility to start cerebral oximetry monitoring within 6 hours from birth, and prior informed parental consent unless deferred consent or opt-out was used). Because the follow-up study was not planned from the outset, some sites, particularly those initiating randomization later, were able to include it in their initial ethical approvals and consent procedures. Most sites required separate consent once the follow-up study was initiated, in accordance with local ethical requirements. Informed consent was written and obtained from the families of the participants. Central and local monitoring was conducted following Standard Operating Procedures (eMethods in Supplement 3).

### Randomization and Intervention in the SafeBoosC-III Trial

Infants were randomly assigned in a 1:1 ratio to either the cerebral oximetry group or the usual-care group. Randomization was stratified by site and gestational age (above or below 26 weeks of gestational age). In the cerebral oximetry group, infants were monitored for the first 72 hours after birth using a forehead sensor that emitted near-infrared light. A bedside monitor continuously displayed the cerebral oxygen saturation percentage, primarily reflecting oxygen levels in the cerebral veins. If cerebral oxygenation fell below a device-specific hypoxic threshold, a treatment guideline was provided suggesting appropriate clinical actions, ie, potential interventions to normalize cerebral oxygenation.^14^ Infants in the usual-care group did not undergo monitoring with cerebral oximetry but received treatment as usual.^12^

### Primary and Exploratory Outcomes for the 2-Year Follow-Up Study

The dichotomous coprimary outcome was a composite of death or moderate or severe neurodevelopmental disability, assessed at approximately 2 years’ corrected age. Death was defined as death occurring before the 2-year follow-up assessment. Moderate or severe neurodevelopmental disability was defined as the presence of 1 or more of the following criteria: cerebral palsy with a Gross Motor Function Classification System score of greater than or equal to 2, a cognitive score below 85 on the Bayley-III/IV cognitive scores or an another neurodevelopmental assessment score below 2 SDs from the mean, visual impairment defined as a diagnosis of moderate reduced vision or worse (blind in 1 eye with good vision in the contralateral eye or blind or can only perceive light or light reflecting objects), and hearing impairment defined as diagnosis of hearing loss corrected with aids or some hearing that is not corrected by aids or no useful hearing even with aids. The continuous coprimary outcome was the cognitive score on the Bayley-III/IV cognitive scores assessment, conducted by a trained professional. All components of the dichotomous coprimary outcome were reported separately as exploratory outcomes. Further exploratory outcomes included head circumference, height, and body weight; daily medication for the last 2 months; and any other chronic illness.^10^

### Data Collection and Outcome Assessment

The SafeBoosC-III follow-up study builds on routinely collected clinical data and parental questionnaires. To minimize missing data, data collection accepted a wider than normal range of age at assessment and followed a prioritized 3-tier model, focusing on the coprimary outcome of death or moderate or severe neurodevelopmental disability (eFigure 1 in Supplement 3).

#### Tier 1: Formal Clinical Data

The prioritized data source was clinical follow-up records from 18 to 30 months’ corrected age. The collected data included information on cerebral palsy and Gross Motor Function Classification System classification, diagnoses of impaired vision and/or hearing, and assessment of cognitive function. The priority for the cognitive function was the Bayley-III/IV cognitive score. If unavailable, other tests measuring a cognitive domain were used according to predefined selection (eMethods in Supplement 3). Exploratory outcomes were also obtained from clinical records.

#### Tier 2: Parental Questionnaires

Access to an online questionnaire was distributed to parents of all eligible infants between 23.5 and 27.5 months’ corrected age. If components of the coprimary outcome death or moderate or severe neurodevelopmental disability were missing from the formal clinical data, parental responses were used. The questionnaires included the following (1) The Parent Report of Children’s Abilities-Revised Nonverbal Cognitive (PARCA-R NVC) scale with scores below 2 SDs classified as events and (2) questions regarding general health and development (eFigure 4 in Supplement 3). Parental confirmation of any of the following resulted in categorization of moderate or severe neurodevelopmental disability: a physician diagnosis of cerebral palsy, inability to walk independently at 2 years’ corrected age, visual impairment (blindness in 1 or both eyes or poor vision even with correction), or hearing impairment requiring hearing aids or cochlear implants. For exploratory outcomes, these questionnaires additionally gathered data on chronic diseases, daily medication use, hospitalizations since initial discharge, infants thriving, parental concerns regarding infant development, and parental education.

#### Tier 3: Informal Assessments

If no formal neurodevelopmental test or PARCA-R NVC scores were available, the cognitive component was assessed informally. If formal clinical data and parental questionnaires were unavailable, all clinical records from 12 months’ corrected age onward were used to determine moderate or severe neurodevelopmental disability. A detailed description of the 3-tier model is available in the eMethods in Supplement 3.

### Blinding

Parents and clinicians were not blinded to group allocation. A blinded outcome assessor reviewed the infants’ clinical records, decided on the classification of outcomes, and reported findings in the electronic case report form (eFigure 3 in Supplement 3). To ensure sufficient blinding of the outcome assessor, a local blinding procedure was developed by each site (eMethods in Supplement 3). Statisticians, data managers, and authors were blinded to group allocation. After analysis and prior to unblinding, abstracts for both outcome scenarios were written and agreed on by the authors.

### Statistical Analysis

A total of 1601 infants were included in the SafeBoosC-III trial.^7^ Assuming that all 1601 infants participated in this follow-up study, power calculations for the dichotomous, coprimary outcome of death or moderate or severe neurodevelopmental disability determined 80% power to detect an 8% absolute risk difference from an expected 50% incidence^15^ of the coprimary outcome, with a 2-sided α level of 2.5%. The Bonferroni adjustment was used to account for 2 coprimary outcomes.^16^ This power calculations was done before the results of the SafeBoosC-III trial and site withdrawals/exclusions. Due to the low maximum power for a large effect, a more sensitive but less patient-relevant coprimary outcome was chosen. Based on answers to a questionnaire on systematic routine follow-up, we assumed that two-thirds of the sites participating in SafeBoosC-III would be able to provide data on Bayley-III/IV cognitive score and that these sites would recruit a total of 850 infants. This sample size would provide 90% power to detect a mean difference of 5 points (Cohen *d* = 0.25) on the mean cognitive score, assuming a SD of 20 points and a 2-sided α level of 2.5%.^10^

Statistical analyses were conducted independently by M.H.O. and J.C.J.. The results of these analyses were compared for discrepancies before unblinding. No significant discrepancies were found. All primary outcomes analyses were performed on the intention-to-treat population. Dichotomous outcomes were analyzed using mixed-effects logistic regression, while continuous outcomes were analyzed using mixed-effects linear regression. All regression models included the trial site as a random effect and gestational age below or above 26 weeks’ and group allocation as fixed effects.^13^ We assumed data were missing at random and used multiple imputation for missing dichotomous coprimary outcomes based on predefined 36-week covariates.^11^ We did not apply imputation to the continuous coprimary outcome due to the high proportion of missing data.^16^ Prespecified sensitivity analyses were conducted for the coprimary outcomes, including a per-protocol analysis; a random-effects meta-analysis to account for possible between-site heterogeneity in treatment effect and a generalized estimation equation analysis to account for nonindependence of multiple births. Exploratory outcomes were analyzed without adjustment for multiple testing and results are presented with effect estimates and 95% CIs and should only be hypothesis generating. Statistical assumptions were systematically assessed for each method. Data were analyzed from October to December 2024 using R, version 4.4.2 (R Foundation for Statistical Computing), by M.H.O. and Stata, version 17 (StataCorp) by J.C.J.

## Results

### Participants

Of the 1601 infants initially randomized from the 70 participating sites, 1438 infants (90%; mean [SD] age, 26.0 [1.3] weeks; 680 female [47.3%]; 758 male [52.7%]) from 56 sites in Asia, Europe, and North America were included in the follow-up study, with 697 in the cerebral oximetry group and 741 in the usual-care group (Figure). A total of 149 infants were lost to follow-up due to the following: 8 declined consent to use data, 38 had clinical follow-up in other hospital where outcome data were unavailable, 34 families moved away, 35 were due to other reasons, and 34 were unknown reasons. Characteristics were similar between the cerebral oximetry and usual-care group at birth and 36 weeks’ postmenstrual age (Table 1). Characteristics of infants eligible for the follow-up study and those from sites withdrawn or excluded were similar at 36 weeks’ postmenstrual age as well (eTable 1 in Supplement 3). An overview of follow-up times and the health care professionals involved can be found in eTables 14 to 16 in Supplement 3.

**Figure.**
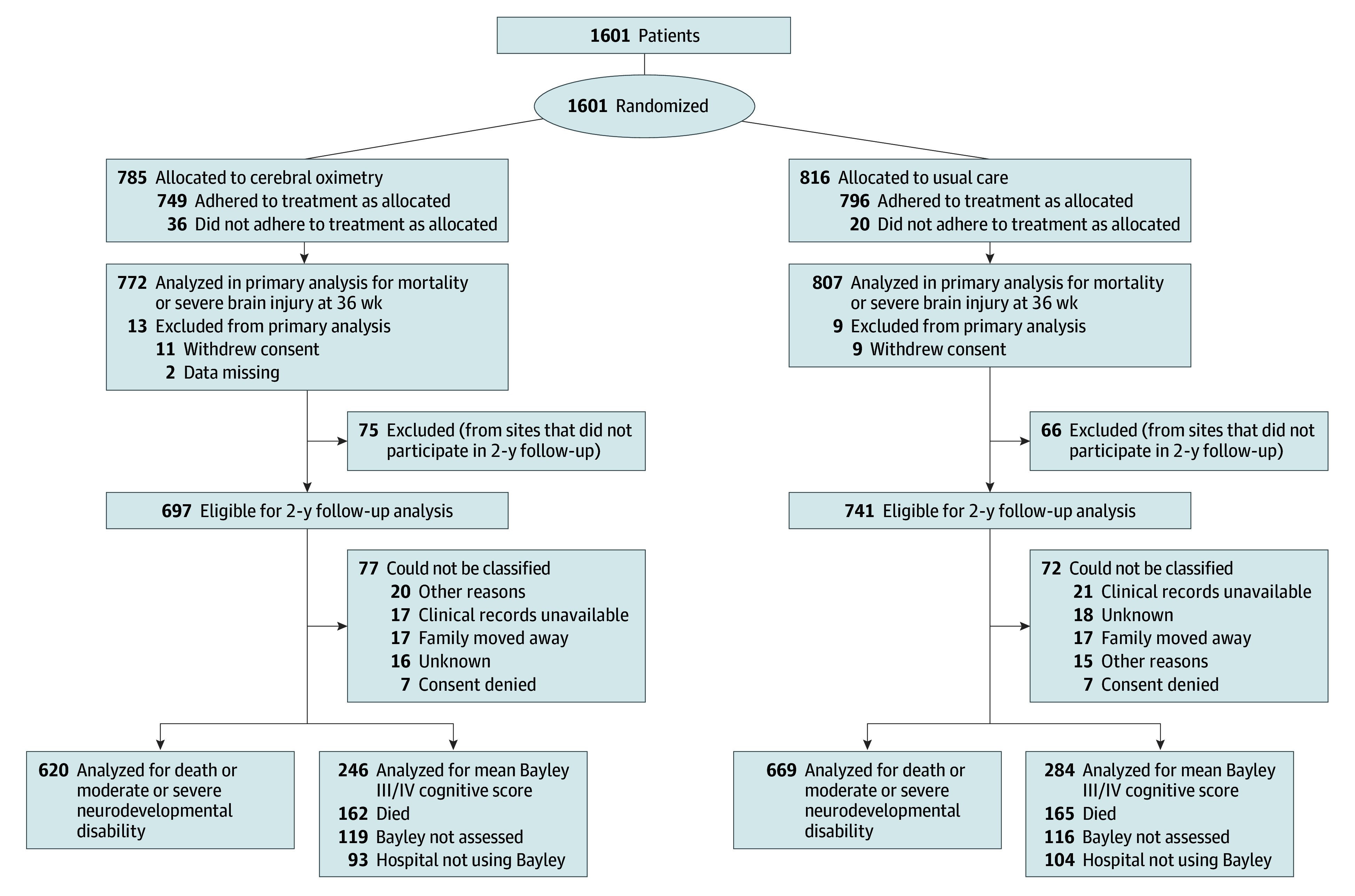
Flowchart Depicting Randomization, 36-Week Follow-Up, and 2-Year Follow-Up

**Table 1.** Baseline Characteristics and Neonatal Clinical Characteristics of the Infants Randomized at the 56 Sites Who Took Part in the 2-Year Follow-Up Study

Characteristic	Cerebral oximetry (n = 697)	Usual care (n = 741)
Birth weight, median (IQR), g	800 (660-958)	800 (660-950)
Gestational age, median (IQR), wk	26.1 (25.0-27.1)	26.1 (25.0-27.1)
Gestational age >26 wk, No. (%)	381 (54.7)	409 (55.2)
Twins or triplets, No. (%)^a^	168 (24.1)	219 (29.6)
Sex, No. (%)		
Female	322 (46.2)	358 (48.3)
Male	375 (53.8)	383 (51.7)
Apgar score at 5 min, median (IQR)^b^	7 (6-8)	7 (6-8)
Neonatal clinical characteristics		
Major congenital anomaly, No. (%)	15 (2.2)	17 (2.3)
Cardiovascular support within 72 h of life, No. (%)	256 (36.7)	227 (30.6)
Mechanical ventilation, No. (%)^c^	551 (79.1)	577 (77.9)
Median days using mechanical ventilation (IQR), No.^d^	9 (3-23)	9 (3-25)
Bronchopulmonary dysplasia, No./total No. (%)^e^	287/524 (54.6)	340/572 (59.4)
Retinopathy of prematurity, No. (%)^f^	97 (13.9)	88 (11.9)
Late-onset sepsis, No. (%)^g^	425 (61.0)	470 (63.4)
Necrotizing enterocolitis, No. (%)^h^	87 (12.5)	82 (11.1)
Severe brain injury at 36 wk PMA, No./total No. (%)^i^	171/688 (24.8)	170/732 (23.2)
Death before 36 wk PMA, No. (%)	150 (21.5)	147 (19.8)
Intervention: cerebral oximetry		
Age at initiation of cerebral oximetry monitoring median (IQR), h	3 (2-4)	NA
Cerebral oximetry monitoring discontinued >14 h, No. (%)	32 (4.6)	NA
Change of medical management due to cerebral hypoxia, No. (%)	199 (28.6)	NA
Cerebral oximetry monitoring in usual care group, No. (%)	NA	20 (2.6)

Abbreviations: NA, not applicable; PMA, postmenstrual age.

^a^ For twins/triplet status, data were available for 694 in the cerebral oximetry group and 741 in the usual-care group.

^b^ For median Apgar score at 5 minutes, data were available for 694 in the cerebral oximetry group and 740 in the usual care group.

^c^ Mechanical ventilation was defined as invasive mechanical ventilation delivered by means of an endotracheal tube or tracheostomy tube.

^d^ For median days using mechanical ventilation, data were available for 551 in the cerebral oximetry group and 577 in the usual-care group.

^e^ Bronchopulmonary dysplasia was defined as the receipt of any respiratory support or supplemental oxygen (or both) at 36 weeks postmenstrual age.

^f^ Retinopathy of prematurity was defined as stage 3 or above (as classified according to the International Classification of Retinopathy of Prematurity) or treatment at any time point until 2-year follow-up.

^g^ Late-onset sepsis was defined as the initiation of antibiotics later than 72 hours from birth for at least 5 days.

^h^ Necrotizing enterocolitis was defined as stage 2 or higher based on the modified Bell staging system or focal intestinal perforation at any time point until 36 weeks follow-up (or both).

^i^ Severe brain injury was defined as one or more of the following diagnoses: intraventricular hemorrhage grade 3 or 4, cystic periventricular leukomalacia, posthemorrhagic ventricular dilatation, cerebellar hemorrhage, or cerebral atrophy shown on any routine cerebral ultrasound scan conducted until 36 weeks follow-up.

### Outcomes

The coprimary outcome death or moderate or severe neurodevelopmental disability was available for 1289 of 1438 participants (90%). In total, 1002 infants (77.7%) underwent categorization of cerebral palsy and visual, hearing, and cognitive impairment using formal clinical records (tier 1), 154 (11.9%) were classified using data from parental questionnaires (tier 2), and 133 (10.3%) were classified from informal assessment (tier 3) (Table 2). Bayley-III/IV cognitive scores were available for 533 of 1111 alive infants (48%).

**Table 2.** Distribution of Data Sources Available to Classify the Coprimary Outcome Death or Moderate or Severe Neurodevelopmental Disability (Percentages Calculated Without Missing Data)

Overall (N = 1289)	Tier		
	1: Death or formal clinical records between 18-30 mo	2: Parental questionnaire	3: Informal assessment of clinical records from 12 mo and onward
Classification of death or moderate or severe neurodevelopmental disability, No. (%)	1002 (77.7)	154 (11.9)	133 (10.3)
Components			
Classification of death, No. (%)	327 (100)	NA	NA
Classification of cerebral palsy, No. (%)^a^^,^^b^	1186 (93.0)	89 (7.0)	NA
Classification of visual impairment, No. (%)^a^^,^^c^	1185 (93.1)	88 (6.9)	NA
Classification of hearing impairment, No. (%)^a^^,^^d^	1184 (92.9)	91 (7.1)	NA
Classification of cognitive impairment, No. (%)^a^^,^^e^	967 (77.0)	165 (13.1)	124 (9.9)

Abbreviations: NA, not applicable; PARCA-R NVC, Parent Report of Children’s Abilities-Revised Nonverbal Cognitive.

^a^ Deaths are classified within tier 1 and are included in the composite outcome of death or moderate or severe neurodevelopmental disability.

^b^ Cerebral palsy was defined as a Global Motor Function Classification score ≥2.

^c^ Visual impairment was defined as a diagnosis of moderate reduced vision or worse (blind in 1 eye with good vision in the contralateral eye or blind or can only perceive light or light reflecting objects).

^d^ Hearing impairment was defined as diagnosis of hearing loss corrected with aids or some hearing but loss not corrected by aids or no useful hearing even with aids.

^e^ Cognitive impairment was defined as (1) Bayley-III/IV cognitive score <85 (1st priority); (2) any developmental assessment <−2SD (including the PARCA-R NVC score) (2nd priority); and (3) if none of the above are available, blinded assessment of health care records from 12 months of corrected age and onwards concluding if the child has a cognitive impairment equivalent to moderate or severe neurodevelopmental disability (3rd priority).

At 2 years’ corrected age, death or moderate or severe neurodevelopmental disability occurred in 292 of 620 infants (47.1%) in the cerebral oximetry group compared with 321 of 669 infants (48.0%) in the usual-care group (relative risk with cerebral oximetry, 0.96; 97.5% CI, 0.85-1.07; *P* = .45) (Table 3). The mean (SD) Bayley-III/IV cognitive score was 92.8 (17.3) in the cerebral oximetry group compared with 93.2 (17.0) in the usual-care group (mean difference with cerebral oximetry, −0.14; 97.5% CI, −3.24 to 2.96; *P* = .92) (Table 3). No important differences were observed when comparing the exploratory outcomes between the cerebral oximetry and usual-care groups (Table 3). Death beyond 36 weeks’ postmenstrual age was rare (eTable 2 in Supplement 3). Parental education did not differ between groups (eTable 16 in Supplement 3).

**Table 3.** Outcomes at 2 Years’ Corrected Age^a^

Outcome	Cerebral oximetry (n = 697)	Usual care (n = 741)	Adjusted RR or mean difference (97.5% CI)	*P* value
Coprimary outcomes				
Death or moderate or severe neurodevelopmental disability, No./total No. (%)	292/620 (47.1)	321/669 (48.0)	0.96 (0.85 to 1.07)	.58
Bayley III/IV cognitive score, mean (SD)	92.8 (17.3)	93.18 (17.0)	−0.14 (−3.24 to 2.96)^b^	.92
No. assessed	249	284	NA	NA
			**RR or mean difference (95% CI)**	
Components of dichotomous outcome, No./total No. (%)				
Death	162/625 (25.9)	165/671 (24.5)	1.01 (0.83 to 1.21)	NA
Cerebral palsy^c^	25/453 (5.5)	22/495 (4.4)	1.30 (0.79 to 2.14)	NA
Visual impairment^d^	25/453 (5.5)	43/493 (8.7)	0.56 (0.38 to 0.8)	NA
Hearing impairment^e^	16/451 (3.5)	14/497 (2.8)	1.15 (0.69 to 1.92)	NA
Cognitive impairment^f^	106/442 (24.0)	116/485 (24.0)	0.97 (0.77 to 1.22)	NA
Moderate or severe neurodevelopmental disability decided by informal assessment	5/60 (8.3)	14/73 (19.2)	0.58 (0.25 to 1.37)	NA
Exploratory outcomes				
Head circumference, median (IQR), cm	47.5(46.0 to 49.0)	47.0 (46.0 to 49.0)	0.03 (−0.27 to 0.34)^b^	NA
No. assessed	221	256	NA	NA
Height, median (IQR), cm	85.0 (82.0 to 88.0)	85.0 (82.0 to 88.0)	−0.20 (−0.87 to 0.46)^b^	NA
No. assessed	222	258	NA	NA
Body weight, median (IQR), kg	11.1 (10.0 to 12.0)	11.1 (10.0 to 12.3)	−0.10 (−0.35 to 0.14)^b^	NA
No. assessed	233	274	NA	NA
Any other chronic illness, No./total No. (%)	117/451 (26.0)	154/488 (31.2)	0.80 (0.65 to 0.98)	NA
Any daily medication for the last 2 mo, No./total No. (%)	93/451 (20.6)	108/488 (22.0)	0.91 (0.71 to 1.17)	NA
PARCA-R NVC score, No. assessed (mean score [SD])	320 (86.4 [21.2])	337 (87.4 [21.3])	0.00 (−4.0 to 3.0)^a^	NA
Hospitalization since discharge from birth hospitalization, No./total No. (%)	181/349 (52.0)	186/381 (48.8)	1.06 (0.91 to 1.23)	NA
Parental report of thriving child, No./total No. (%)	337/348 (97.0)	368/381 (96.6)	1.00 (0.97 to 1.03)	NA
Parental report of worries regarding the child, No./total No. (%)	124/341 (36.0)	144/374 (38.5)	0.96 (0.79 to 1.17)	NA

Abbreviations: PARCA-R NVC, Parent Report of Children’s Abilities-Revised Nonverbal Cognitive; RR, relative risk.

^a^ Effect estimates for the coprimary outcomes are derived from prespecified regression models. The dichotomous outcome (death or moderate or severe neurodevelopmental disability) was analyzed using mixed-effect logistic regression, while continuous outcome (Bayley III/IV cognitive score mean score) was analyzed using mixed-effect linear regression. Models were adjusted for stratification variables. Two-sided *P* values correspond to these model-based estimates. CIs for the coprimary outcomes are reported at the 97.5% level to account for multiplicity.

^b^ For this outcome, the treatment effect is the mean difference.

^c^ Cerebral palsy was defined as a Global Motor Function Classification score ≥2.

^d^ Visual impairment was defined as a diagnosis of moderate reduced vision or worse (blind in 2 eye with good vision in the contralateral eye or blind or can only perceive light or light reflecting objects).

^e^ Hearing impairment was defined as diagnosis of hearing loss corrected with aids or some hearing but loss not corrected by aids or no useful hearing even with aids.

^f^ Cognitive impairment was defined as (1) Bayley-III/IV Cognitive score <85 (1st priority); (2) any developmental assessment below 2 SDs (including the PARCA-R NVC score) (2nd priority); and (3) if none of the above are available, blinded assessment of health care records from 12 months of corrected age and onward concluding that the child has a cognitive impairment equivalent to moderate or severe neurodevelopmental disability (3rd priority).

The sensitivity analyses consistently supported the results in the coprimary outcome death or moderate or severe neurodevelopmental disability. Multiple imputation analyses indicated that missing data did not substantially affect results (eTable 4 in Supplement 3). The generalized estimation equation sensitivity analysis did not suggest that the result was significantly affected by the high proportion of twins (eTable 10 in Supplement 3). The exclusion of informal assessments did not change the results (eTable 6 in Supplement 3). The remaining prespecified analyses are presented in eFigure 2 and eTables 3, 5, 7-9, 11-13, and 17 in Supplement 3.

## Discussion

In this 2-year follow-up of the SafeBoosC-III randomized clinical trial, extremely preterm infants receiving treatment guided by cerebral oximetry monitoring compared with usual care for the first 72 hours after birth did not result in a lower incidence of death or moderate or severe neurodevelopmental disability, nor did it result in higher Bayley cognitive scores.

The findings in this follow-up study are consistent with our 36-week outcome results of the SafeBoosC-III trial.^7^ The confidence limits for the dichotomous outcome are wide, thus not excluding either important potential benefits or harms, and reflect limited statistical power.

In contrast, the confidence limits of the effect size on Bayley-III/IV cognitive scores are narrow, thus excluding a major effect at the population level. Although cognitive tests at school age are more reliable than at 2 years,^17^ the likelihood that effects emerge later is low.^18^ This is supported by an ancillary study^19^ of the SafeBoosC-III trial population, which did not find less abnormalities on magnetic resonance imaging at term equivalent age.

Of the exploratory outcomes, visual impairment was reported less frequently in the cerebral oximetry group (Table 3), whereas severe retinopathy of prematurity was slightly higher in the cerebral oximetry group (Table 1). This difference in visual outcomes likely reflects a chance finding rather than a biologically plausible effect and may partly relate to different definitions of visual impairment.

No evidence of an effect of cerebral oximetry monitoring may have several possible explanations. Monitoring was limited to the first 72 hours after birth, representing a short exposure relative to the overall duration of neonatal intensive care. Hypoxia may not be the primary driver of brain injury in extremely preterm infants as brain injury in this population is multifactorial. Cerebral oximetry with near-infrared spectroscopy measures only from local and superficial layers of the brain and is subject to significant imprecision particularly after sensor repositioning.^20^ To improve the effectiveness of the intervention, a web-based training program was implemented. However, only 39% of staff caring for the participants at the 70 sites obtained certification, as this was not mandatory. This may have led to suboptimal implementation in sites with limited prior clinical experience and modest engagement in the web-based training.^21^ Continuous cerebral oximetry data were not collected, hindering estimation of the baseline burden of hypoxia. Only 29% of the experimental group had management changes due to cerebral hypoxia documented in their clinical records, limiting the intervention’s potential effect to this subgroup. Although hypoxic thresholds triggered alerts, there was no standardized or mandated management response. Finally, a targeted 22% relative risk reduction may have been optimistic and smaller effects may not be detectable by a trial of this size. Cerebral oxygenation may be more directly affected in specific clinical scenarios, such as systemic hypotension, significant anemia, mechanical ventilation, or hemodynamically relevant patent ductus arteriosus, where cerebral perfusion and oxygen delivery are compromised.^22,23,24,25^ Future studies could target such high-risk populations.

### Limitations

Our study has several limitations. First, the SafeBoosC-III follow-up trial was not prespecified as part of the original trial design, and the available sample size was, therefore, not powered to detect differences in long-term outcomes. The targeted sample size for the 2 coprimary outcomes was not achieved, thereby increasing the risk that the study was underpowered. Second, we encountered 10% missing data for the primary outcome of death or moderate or severe neurodevelopmental disability, which may introduce bias dependent on the missing mechanism.^26^ However, the proportion of missing data was similar between the cerebral oximetry and usual-care groups, and multiple imputation analysis did not alter the intervention effect. The Bayley-III/IV cognitive scores were available for only 48% of eligible alive infants. The prespecified sample size calculation targeted a mean difference of 5 points that, while modest at the individual level, may be considered clinically relevant at the population level for an intervention with few adverse effects. Third, routine clinical assessments and reporting of these in the clinical records were not blinded. Group allocation is unlikely to have influenced clinical assessments, as the intervention only lasted the first 3 days after birth, in a hospital stay that for survivors may last several months. To reduce potential bias, extraction of outcome data from clinical records was performed blindly. Fourth, the nonparticipation of 14 sites may reduce external validity, but similarly, the 36-week outcomes between excluded and included sites suggest minimal impact. Fifth, informal assessments provided 10% of the data for the dichotomous coprimary outcome. While subjective, these assessments were blinded, minimizing the risk of systematic bias, and results did not differ materially when these data were excluded. Importantly, our 3-tier model enabled a 90% follow-up rate for the dichotomous coprimary outcome, despite the lack of funding for trial-specific follow-up at local sites. This is both a strength but also a limitation as this pragmatic approach prioritized completeness of follow-up over the uniform data granularity seen in population-based cohorts or studies with dedicated trial-specific assessments. By minimizing missing data, a common challenge in 2-year outcome studies, this model offers potential for adaptation in other neonatal trials assessing similar long-term outcomes. A limitation may also be the pragmatic design of the trial: the intervention was limited to the first 72 hours after birth, training in the use of cerebral oximetry was variable across sites, and no details were collected on the clinical actions taken when hypoxic thresholds were reached, the response, nor on other patient monitoring data. These methodological limitations should be considered when interpreting the results.

## Conclusions

In this follow-up study of the SafeBoosC-III randomized clinical trial, in extremely preterm infants, treatment guided by cerebral oximetry monitoring compared with usual care for the first 72 hours after birth did not result in a lower incidence of death or moderate or severe neurodevelopmental disability, nor did it result in higher Bayley cognitive scores at 2 years’ corrected age. The routine use of cerebral oximetry monitoring during the first 72 hours after birth in extremely preterm infants to reduce death or moderate or severe neurodevelopmental disability and cognitive impairment was not supported by this trial.

## Notes

Supplement 1.
Trial Protocol.

Supplement 2.
Statistical Analysis Plan.

Supplement 3. **eAppendix.** List of Investigators
eMethods.
**eTable 1.** Death or Severe Brain Injury at 36 Weeks’ Postmenstrual Age and at 2-Year Follow-Up in Included and Excluded Sites **eTable 2.** Causes of Death Beyond 36 Weeks’ Postmenstrual Age **eTable 3.** Per-Protocol Analysis **eTable 4.** Multiple Imputation Analysis on Death or Moderate or Severe Neurodevelopmental Disability **eTable 5.** Best-Worst and Worst-Best Case Analysis on Death or Moderate or Severe Neurodevelopmental Disability **eTable 6.** Exclusion of Informal Assessment (Tier 3) for the Coprimary Outcome Death or Moderate or Severe Neurodevelopmental Disability **eTable 7.** Number of Events in Coprimary Outcome Death or Moderate or Severe Neurodevelopmental Disability for Informal Assessments (Tier 3) **eTable 8.** Comparison of Sites With High Follow-Up Rates (≥90%) vs Low Follow-Up Rates (<90%) for the Coprimary Outcome Death or Moderate or Severe Neurodevelopmental Disability **eTable 9.** Comparison of Sites With High Follow-Up Rates (≥90 vs Low Follow-Up Rates (<90%) for the Coprimary Outcome Bayley III/IV Cognitive Score **eTable 10.** Generalized Estimation Equation (Twin Sensitivity Analysis) **eTable 11.** Proportion of Missingness Between Cerebral Oximetry Group and Experimental Group for the 2 Coprimary Outcomes **eTable 12.** Analysis of all PARCA-R Nonverbal Cognitive Scores Including Extrapolated Scores **eTable 13.** Death or Moderate or Severe Neurodevelopmental Disability With PARCA-R Nonverbal Cognitive Extrapolated Scores **eTable 14.** Follow-Up Times of Randomized Infants **eTable 15.** Clinical Data Based on Information From Health Care Professional **eTable 16.** Parental Education Level Based on ISCED Classification **eTable 17.** Number of Randomizations per Site **eFigure 1.** Prioritization of Data for the Coprimary Outcome Moderate or Severe Neurodevelopmental Disability **eFigure 2.** Random Effect Meta-Analysis for Coprimary Outcome Moderate or Severe Neurodevelopmental Disability and Bayley III/IV Cognitive Score **eFigure 3.** Extract From the Electronic Case Report Form for 2-Year Clinical Data **eFigure 4.** Parental Questionnaire Used for the SafeBoosC-III Follow-Up Study
eReferences.

Supplement 4. **Nonauthor Collaborators.** SafeBoosC-III Follow-Up Collaborator Group

Supplement 5.
Data Sharing Statement.
