# Supplementary material for: Cerebral Oximetry in Extremely Preterm Infants: 2-Year Follow-Up of the SafeBoosC-III Randomized Clinical Trial
Source: JAMA Pediatr. 2026 Apr 20;180(6):619–27. doi: 10.1001/jamapediatrics.2026.1066 (PMC13097032; doi:10.1001/jamapediatrics.2026.1066)
Supplement: Supplement 1. — Trial Protocol. [file jamapediatr-e261066-s001.pdf]

SafeBoosC III two-year follow-up

Safeguarding the Brain of our smallest Children

Cerebral near-infrared spectroscopy monitoring versus treatment as usual for extremely preterm infants: a protocol for the follow-up study for the SafeBoosC-III trial

Sponsor:

Region Hovedstaden
Department of Neonatology 5024, Rigshospitalet
Blegdamsvej 9, 2100 Copenhagen Ø, Denmark
Phone: +45 3545 1326
Email: gorm.greisen@regionh.dk

SafeBoosC III
SAFEGUARDING THE BRAIN OF OUR SMALLEST CHILDREN

Revision history

Version	Author	Date	Major changes
1.0 draft	The SafeBoosC-III Trial Group	17.05.2021	Initial version
2.0 draft	The SafeBoosC-III Trial Group	27.05.2021	• Condensing of exploratory outcomes<br>• Addition of Bayley IV to the co-primary outcomes and handling of scores
1.0 protocol	The SafeBoosC-III Trial Group	25.06.2021	• Approval by Steering Trial Group

Summary

Background

In the SafeBoosC-III trial, the objective is to investigate the effect of treatment based on cerebral near-infrared spectroscopy (NIRS) monitoring of brain oxygenation compared with treatment as usual in extremely preterm infants. The primary outcome of the trial is death or survival with severe brain injury at 36 weeks' postmenstrual age. Despite an association between severe brain injury diagnosed in the neonatal period and later neurodevelopmental disability, this relationship is not always strong. Therefore, if treatment based on cerebral NIRS monitoring decreases the risks of death or survival with severe brain injury, it is important to document if the beneficial effect persists into early childhood, in the form of better neurodevelopmental outcome. Furthermore, it is also important to identify evidence of unexpected harms. It would be unfortunate if cerebral NIRS monitoring became standard practice without good evidence that long-term patient-relevant benefits outweigh possible harms. In the SafeBoosC phase-II trial, 115/135 alive infants were followed-up at 24 months' corrected age to test if the intervention was beneficial in terms of improving neurodevelopment. No significant differences were found between the experimental versus control group. However, the trial was not powered to detect differences in long-term clinical outcomes. As the SafeBoosC-III trial intends to randomise 1600 infants, there is potential to achieve sufficient power for a meaningful assessment of the experimental intervention's effect on long-term neurodevelopment, as well as an evaluation of unexpected harms.

Methods

In total, 1600 infants will be randomised in the SafeBoosC-III trial. We expect approximately 1250 infants to survive and potentially be available for follow-up.

Inclusion criteria: Participation in the SafeBoosC-III trial and enrollment in a neonatal intensive care unit (NICU) taking part in the follow-up study, with parental consent according to local regulations.
Exclusion criteria: Parental objection to the use of their child's data in the follow-up study.
Participation criteria for NICUs: All NICUs participating in the SafeBoosC-III trial will be invited.

Assessments: We aim to collect follow-up data on as many children as possible by including data from routine neurodevelopmental follow-up programmes, utilizing all other health care records from the age of at least 12 months and by parental questionnaires including the PARCA-R.
Blinding: Due to the nature of the trial intervention, children and their parents will not be blinded to treatment allocation. A local co-investigator who is blinded to group allocation will conduct outcome assessments. To ensure that outcome assessment from health care records is blinded, the principal investigator and the co-investigator from each NICU will develop a local blinding procedure describing their individual workflow. Each local NICU blinding process will be approved by the central trial unit. Data managers, statisticians and those writing the abstracts as well as drawing conclusions will also be blinded.

Outcomes

There will be two co-primary outcomes:
Dichotomous effect measure: death or moderate-or-severe neurodevelopmental disability

A child will be classified with moderate-or-severe neurodevelopmental disability if any of the following conditions are present: cerebral palsy with a Gross Motor Function Classification Score ≥ 2; a cognitive function score below -2 standard deviations from the norm (or Bayley-III/IV < 85); hearing impairment; or vision impairment. Available data from at least 12 months' corrected age from health care records, including standardised neurodevelopmental assessments, as well as parental questionnaires completed between 18-30 months' corrected age will be used to assess mortality and neurodevelopment.

Power calculation: Based on answers from the questionnaire on systematic routine follow-up, as well as implementing parental questionnaires and informal assessments to classify neurodevelopment, we estimate the total sample size for the outcome death or moderate-or-severe NDD to 1600 children. Based on results from two randomised controlled clinical trials investigating neuroprotection in extremely preterm infants, it is estimated that the proportion of children with the outcome of death or moderate-or-severe NDD will be 50% in the control group.
An indicative power calculation shows that if we want to test a risk difference of eight percent between the experimental and control group, at an alfa of 2.5% and a sample size of 800 infants in each group, i.e. a total of 1600, we will reach a power of 80%.

Continuous effect measure: mean Bayley III/IV cognitive score

The cognitive scores from the Bayley III/IV assessment will constitute a single continuous outcome measure.

Power calculation: Based on answers to an investigator questionnaire, we expect to have access to data from Bayley III/IV assessments of 850 children. An indicative power estimation shows that if we want to test a mean difference of five points on the Bayley cognitive score, with a standard deviation of 20 (Cohens d' 0.25) between the experimental and the control group, at an alfa of 2.5%, and a sample size of 425 infants from each group i.e. a total of 850, we will reach a power of 90%.

The exploratory outcomes will be daily medication the last two months (yes/no), any other chronic illness (defined as any problem which has been diagnosed by a doctor and which 1) is expected to last more than a few months, 2) causes problems in everyday life, or 3) is a risk of early death or disability), mean head circumference, mean height and mean body weight.

All components of the co-primary outcomes will be reported for the two groups separately as well as effect estimates with confidence intervals and will be taken into consideration when interpreting the main results

Time plan

The first randomised infant in SafeBoosC III will reach two years of corrected age in September 2021, while the last follow-up assessment is expected to be completed before the end of 2024, which is an estimate of when the last infant randomised will reach two years of corrected age plus six months of reserve.

Organisation and funding

The SafeBoosC-III trial Steering Committee will be responsible for principal decisions. Professor Gorm Greisen will be the Coordinating Investigator. Local expenses must be covered by each NICU. The central expenses will be covered by the Sponsor (The Capital Region of Denmark, Copenhagen University Hospital).

1. Background and introduction

1.1 The population and condition

Worldwide, approximately 15 million infants are born preterm (below 37 weeks' gestational age) each year. Hereof, around 50,000 are born extremely preterm (below 28 weeks' gestational age) in countries where neonatal intensive care is offered routinely (1). The advances in neonatal intensive care over the past decades have resulted in a significant increase in the survival of extremely preterm infants (2-5). Currently, mortality is around 20%, but those surviving have a high risk of neonatal brain injury and subsequently long-term neurodevelopment disability (6). The risk of brain injury is especially high during the first days of life, as the infant transitions from intra- to extrauterine life. The combination of immature organs and impaired cerebral autoregulation may cause large fluctuations in cerebral blood flow (7), detectable by cerebral oximetry using near-infrared spectroscopy (NIRS). Brain injury acquired in the neonatal period may result in neurodevelopmental disability later in life. It is estimated that as many as 40% of surviving extremely preterm infants suffer from long-term neurodevelopmental disability such as cerebral palsy, cognitive and neurosensory deficits, attention deficit disorder, and major psychiatric disorder (8). These disabilities result in life-long consequences for the children and their families, such as reduced quality of life as well as increased health care and educational costs (9, 10). Evidence suggests that cerebral hypoxia is associated with the risk of brain injury as well as death in extremely preterm infants. Thus, it is plausible that a reduction of cerebral hypoxia during the first days of life may reduce the risk of severe brain injuries (11) and thus the risk of later neurodevelopmental disability (12).

1.2 The SafeBoosC consortium

1.2.1 The SafeBoosC II trial

The SafeBoosC phase-II trial demonstrated that NIRS monitoring in combination with an evidence-based treatment guideline reduced the burden of cerebral hypoxia and hyperoxia by more than 50%, compared with treatment as usual, during the first 72 hours of life (13). Furthermore, there were trends towards reduced mortality and occurrence of severe brain injury assessed at 36 weeks'

postmenstrual age (PMA). In total, 115/135 (85%) infants that were alive at 24 months' corrected age were followed-up to test if the intervention was beneficial in terms of improving neurodevelopment later in life. No differences were found between the experimental and control group in regards to the mean mental developmental index assessed by the Bayley II (89.6±19.5 versus 88.4±14.7, p=0.77) or the total Ages and Stages Questionnaire score (215±58 versus 213±58, p=0.88). The number of infants with moderate-or-severe neurodevelopmental disability were also similar, with ten (15%) in the experimental group and six (12%) in the control group (p = 0.58) (12). However, the SafeBoosC II trial was not powered to detect a relevant difference on any of the long-term clinical outcomes and thus there is a possibility that the neutral results were due to type II errors (14). Based on the above, the larger SafeBoosC-III trial is now being conducted, to test the effect of the intervention on clinical outcomes.

1.2.2 The SafeBoosC III trial

SafeBoosC III is a multi-centre, international, pragmatic phase III clinical trial investigating the effects of treatment guided by cerebral NIRS monitoring in extremely preterm infants (15). The hypothesis is that treatment based on cerebral NIRS monitoring of cerebral oxygenation in extremely preterm infants will result in a reduction of death or severe brain injury at 36 weeks' PMA. Infants randomised to the experimental group are monitored during the first 72 hours of life and receive cardio-respiratory support guided by cerebral NIRS monitoring (11). The NIRS sensor must be placed within six hours from birth. The control group does not receive any cerebral NIRS monitoring and is treated according to local guidelines and clinical practices. The SafeBoosC III trial will randomise 1600 infants, having started in June 2019 with recruitment expected to be completed by the end of 2021. Nearly 90 neonatal intensive care units (NICUs) from Europe, USA, China and India have signed up to take part in the trial (15). As of May 1st 2021, over 1000 infants have been randomised. The trial is registered at clinicaltrials.gov (NCT03770741).

The original sample size of the SafeBoosC III trial is calculated based on the primary outcome, which is a composite of death or severe brain injury at 36 weeks' PMA (16). Severe brain injury is defined as cerebral haemorrhage grade III or IV, cystic periventricular leukomalacia, cerebellar haemorrhage, post-haemorrhagic ventricular dilatation or cerebral atrophy detected on any of the cerebral ultrasound scans that are routinely performed in these infants up until 36 weeks' PMA or discharge to home, whichever comes first.

1.3 Assessment of brain injury and neurodevelopment

1.3.1 Ultrasound

Cerebral ultrasound is used to diagnose neonatal brain injury in the SafeBoosC III trial at 36 weeks' PMA. Severe (grade III or IV) periventricular-intraventricular haemorrhage is associated with an increased risk of adverse neurodevelopmental outcomes, including cerebral palsy and lower cognitive scores, but odds ratios are only 2 to 3 (18). Cystic periventricular leukomalacia is less common, but a stronger predictor of cerebral palsy and developmental delays for extremely preterm infants (19).

Cerebral ultrasound is less sensitive in the detection of mild to moderate types of brain injury as compared to magnetic resonance imaging (20, 21). This can potentially result in an underestimation of infants at risk of long-term neurodevelopmental disabilities. For example, in one study, among extremely preterm infants with no ultrasound abnormalities in the neonatal period, 23% had delayed mental development and 26% had delayed psychomotor development at two years of age (19). Thus, despite neonatal brain injury diagnosed by ultrasound being a clinically relevant outcome, it is not a definitive surrogate for long-term neurodevelopment, a more patient-relevant outcome. Therefore, assessment later in life is also important.

1.3.2 Neurodevelopmental assessment

While neuropsychological abilities can only fully be assessed in adulthood, very young childrens' abilities to walk, talk, see, hear, and develop at approximately the same pace as peers, is relevant in itself for the full life of a child, as well as being relevant milestones in the overall neurodevelopmental trajectory. Therefore, assessment in early childhood is relevant, optimally by a standardised neurodevelopmental assessment, which measures the child's skills compared to a norm. Assessment at two years of corrected age is commonly used clinically as well as for research purposes (22). The predictive value of such assessments for later function is relatively poor for the individual child, except for the most severe disabilities with score values below minus 3 SD (23, 24). This is likely partly due to the difficulties of assessing young, intermittently cooperating children, but also due to the irregular pace of development in individual infants, as well as the fact that some elements of cognition only develop later, and that childhood and upbringing also influence development. As an example, while measures of intelligence quotient (IQ) track well

from the age of five years and onwards through adult life, measures of cognition at two years do not correlate well with measures of IQ at 5 years (8). However, on a group level, these assessments fairly consistently quantify differences between extremely preterm born children and children born at term (nearly one SD). Therefore, these assessments are likely to be valuable as outcome measures in randomised clinical trials, since assessments will be compared on a group level (25).

Multiple validated methods exist to assess neurodevelopment in early childhood, with the most frequently used being the Bayley Scales of Infant Development (Bayley) and the Griffiths Scale of Childhood Development (Griffiths) (32,33). However, these assessments are lengthy for both the children, parents and clinicians and reliability is compromised if a child is unable to cooperate well. Parental questionnaires, drawing on the knowledge of the child's abilities in daily life, may be a good, pragmatic and cost-effective substitute for a face-to-face neurodevelopmental assessment by a qualified examiner (26). The Parent Report of Children's Abilities-Revised (PARCA-R) is a parental questionnaire, evaluating cognitive and language development at two years of age (27). It has high sensitivity and specificity for identifying neurodevelopmental disability among very preterm infants, supporting its use as an outcome measure in randomised trials (28, 29). For further elaboration please see section 2.6 'Outcome assessment tools' in the Methods section.

1.4 Potential harms

It would be unfortunate if cerebral NIRS monitoring became standard practice without good evidence that patient-relevant benefits outweigh harms. As an example, studies investigating the use of early systemic postnatal corticosteroids for preventing bronchopulmonary dysplasia (BPD) (30) in preterm infants, at first showed significantly improved short-term outcomes, such as earlier extubation and less prevalence of BPD. However, when two-year follow-up assessments were conducted, several adverse neurological effects were reported, in particular cerebral palsy (31). Based on this, the clinical use of corticosteroids was restricted to those infants at very high risk of BPD or death. Studies like these demonstrate the importance of long-term follow-up for randomised clinical trials.

The SafeBoosC-II trial did indeed show a trend towards higher prevalence of BPD and retinopathy of prematurity in the experimental group. Therefore, it is possible that cerebral NIRS monitoring

may cause harm. Such harms could theoretically be caused by inappropriate modifications in cardio-respiratory support based on hypoxic values and/or unnecessary infant disturbance.

1.5 Recruitment and loss to follow-up

Loss to follow-up in studies with long-term outcomes is common and data are rarely missing completely at random. Studies have shown that children lost to follow-up often have a lower socioeconomic status and tend to have more neurodevelopmental impairment than children participating in follow-up programmes (41). Factors affecting loss to follow-up include maternal education, age, substance abuse, poverty level, and marital status, among others (42). As this is a pragmatic study, we do not expect that all eligible children will participate. However, we aim to ascertain follow-up data on as many children as possible by collecting data from all health care records from 12 months' corrected age onward as well as by parental questionnaires (see Methods section 2.6 'outcome assessment tools').

In the Summer of 2020, when recruitment was well-established, a questionnaire was sent out to all active NICUs asking if they were interested in participating in the follow-up study and how their local follow-up programmes were structured. Sixty-two NICUs completed the questionnaire and all expressed interest in taking part in the two-year follow-up study. All NICUs reported having a systematic follow-up programme of extremely preterm infants. Eighty percent reported a routine follow-up rate of 75% or more, and 65% routinely perform a Bayley III/IV assessment between 18 and 30 months' corrected age. Furthermore, several other standardised developmental assessments and screening methods were reported to be utilized from 18 to 30 months' corrected age, including Griffiths, Denver Developmental Screening Test II, Peabody II, Gessell, Ages and Stages Questionnaire, PARCA-R, Alberta Infant Motor Scales, etc. Almost all NICUs conduct a standardised neurodevelopmental assessment around two years' corrected age. Based on these investigator questionnaire responses, we have planned the SafeBoosC-III two-year follow-up study.

Since the summer of 2020, more NICUs have joined the SafeBoosC-III consortium, thus strengthening the chances of obtaining sufficient statistical power for a meaningful test of the intervention's effect on neurodevelopment.

2 Methods

2.1 Objective and hypothesis

The objective of the SafeBoosC III follow-up study is to investigate the benefits and harms of treatment guided by cerebral NIRS monitoring of brain oxygenation in extremely preterm infants during the first 72 hours of life, assessed at two years' corrected age.

The hypothesis is that the intervention will decrease a composite of death or moderate-or-severe neurodevelopmental disability at two years' corrected age, and/or increase cognitive function in survivors assessed by the Bayley III/IV test, with insignificant harms.

2.2 Inclusion criteria

Participation in the SafeBoosC III trial, enrollment in a NICU taking part in the follow-up study, and parental consent according to local regulations.

2.3 Exclusion criteria

Parental objection to use of their child's data in the study.

2.5 Outcomes

2.4.1 Primary outcomes

The two co-primary outcomes are:

1) Death or moderate-or-severe neurodevelopmental disability (NDD).

A child will be classified with moderate-or-severe NDD if any of the four following conditions are present:

- cerebral palsy with functional impairment corresponding to Gross Motor Function Classification Score (GMFCS) ≥ 2;
- a score below -2 standard deviations from the norm of a standardised developmental assessment (if using the Bayley III/IV test, the cognitive score cut-off will be < 85), or an informal classification of moderate-or-severe NDD;

- vision impairment defined as moderately reduced vision, or only being able to perceive light or light reflecting objects; or blind in one eye with good vision in the contralateral eye.
- hearing impairment defined as hearing loss corrected with aids (usually moderate 40-70dBHL) or some hearing but loss not corrected by aids (usually severe 70-90dBHL).

2) Bayley III/IV mean cognitive score

2.4.2 Exploratory outcomes

The exploratory outcomes are as follows:

- Daily medication the last two months (yes/no)
- Any other chronic illness (defined as any problem which has been diagnosed by a doctor and which 1) is expected to last more than a few months, 2) causes problems in everyday life, or 3) is a risk of early death or disability)
- Mean head circumference
- Mean height
- Mean body weight, with one decimal

All components of the co-primary outcomes will be reported for the two groups separately as well as effect estimates with confidence intervals and will be taken into consideration when interpreting the main results

All outcomes will be assessed from health care records by a blinded assessor at a minimum of 12 months of corrected age.

2.6 Outcome assessment tools

The following outcome assessments tools may be used to collect data on children in the SafeBoosC III follow-up study. We aim to collect data on as many children as possible and will do this by collecting clinical data from health care records as well as answers from parental questionnaires.

2.6.1 The Bayley assessment

The Bayley Scales of Infant Development (Bayley) is a commonly used neurodevelopmental assessment tool for children from 1 to 42 months of age (32). There now exists a second (Bayley II), third (Bayley III) and fourth (Bayley-IV) version. The Bayley II measures a Mental

Developmental Index (MDI), which is based on a composite of early cognitive and language development, as well as a Psychomotor Development Index (PDI), which assesses motor abilities (33). In the revised Bayley III (2008), the two indexes, MDI and PDI, were replaced by five individual subcategories: cognitive, language, motor, social-emotional and adaptive behaviour (34).. The correspondence between the Bayley II and III has been examined in previous studies (12, 34, 35). Bayley III scores are approximately 10 points higher than the Bayley II, thus resulting in less infants being diagnosed with neurodevelopment impairment using conventional cut-offs (-2SD). This has implications for both clinical practice as well as research, where the underestimation of neurodevelopmental impairment may lead to reduced statistical power in randomised trials.
Some NICUs may switch to the Bayley-IV during the SafeBoosC-IIIfu study. In published data from Pearson (52) it is argued that there is evidence to support the accuracy and validity of the Bayley-IV scores. Furthermore, as the Bayley-IV (2019) retains the same subcategories and assessment methods as Bayley III, it predicted yield similar scores to the Bayley III. No randomised clinical trials have used and published results using the Bayley IV as an outcome measure yet, and therefore the scores will be treated equal to the Bayley-III scores in the co-primary outcome Bayley III mean cognitive score. In the event of a possible difference between the Bayley III and IV scores, the nature of a randomised trial and block randomisation will equally distribute the differences between the experimental and control group. Furthermore we will conduct a sensitivity analysis investigating a possible influence on treatment effect caused by differences in Bayley III and IV scores.
For the purpose of the co-primary outcome Bayley III/IV mean cognitive score, the unadjusted mean scores from the experimental and control group will be compared. If the Bayley III/IV cognitive score is used to evaluate cognitive function in co-primary outcome death or moderate-or-severe NDD, the cognitive function cut-off will be <85, as done in previous trials and recommended by experts (35).

2.6.2 Medical examination

A medical examination will ideally include measurement of head circumference, height and weight, and the child's vision and hearing will be tested, either formally or informally. Signs of cerebral palsy is usually be documented as well as motor abilities (40). Furthermore, the presence of chronic illnesses and/or medication usage will be obtained from medical records.

2.6.3 Parental questionnaires

The parental questionnaires will be comprised of the PARCA-R and a health and development questionnaire.

2.6.3.1 PARCA-R

The Parent Report of Children's Abilities – Revised (PARCA-R) is a parental questionnaire that can be used to assess children's non-verbal cognitive and language development at 24 months' corrected age. The PARCA-R provides a high test-retest reliability and correlates well with the Mental Development Index (MDI) of the Bayley II, and with the cognitive score of the Bayley III (28, 36, 37). The questionnaire has been used as a two-year outcome measure in multiple clinical trials (29) and has furthermore been translated into several languages. For this follow-up study, an online platform will be developed, where parents of the participating children will be invited to complete the non-verbal cognitive (NVC) part of the PARCA-R questionnaire, in their respective languages. This typically takes less than 15 minutes to complete (38). The full PARCA-R will not be used, since a validated language component is not available in all countries; PARCA-R has only been standardised for use in the UK. The PARCA-R must be assessed on children between 23.5 and 27.5 months of corrected age. Children will be classified as having moderate-or-severe NDI when scores correspond to a scale score of -2SD from the norm (39).

The NVC scale consists of 34 forced-choice items, scored zero or one, from which a total NVC score is derived, which is adjusted for the child's corrected age. In cases where no physical standardised assessment (i.e. Bayley, Griffiths etc) of the child is available, NVC cut-off scores will be used as the measure of cognitive function for the co-primary outcome death or moderate-or-severe NDD. When more than four items are missing, the NVCs will not be calculated, but will be categorised as "unknown."

2.6.3.2 Health and development questionnaire

The PARCA-R questionnaire will be supplemented with a number of health and developmental questions to be answered by the parents as well. The questions focus mainly on the individual components of the moderate-or-severe NDD outcome definition, as well as quality of life questions. It will be available on the online platform as well. The coding of the health and development questionnaire for the co-primary outcome moderate-or-severe NDD can be found in Appendix B.

2.7 Data extraction

All data extraction from health care records and data entry into the e-CRF will be done by an assessor who is blinded to group allocation (see section '2.8 Blinding'). The co-primary outcome Bayley III/IV mean cognitive score will be extracted from the records of the Bayley III/IV test. The co-primary outcome death or moderate-or-severe NDD will primarily be based on an assessment of available health care records, from when the child was between 18 and 30 months' corrected age. If more than one formal neurodevelopmental assessment has been conducted, the score of the latest should be used. If formal assessment of all four components is not available, the central trial unit will substitute the missing components with answers from parental questionnaires (PARCA-R and health and development questionnaire). The answers from the questionnaires will be reported directly by the parents to the central trial unit. If answers from parental questionnaires are not available, either the PI will be contacted by the central trial unit and requested to provide an informal assessment of the presence or absence of moderate-or-severe NDD. The assessment should be based on all available information from the child from at least 12 months' corrected age. For additional details on this approach, see appendix A.

2.8 Blinding

Due to the nature of the trial intervention, children and their parents will not be blinded to treatment allocation. A co-investigator who is blinded to group allocation will conduct outcome assessments. To ensure that outcome assessment is blinded, the principal investigator and co-investigator from each NICU must develop a local blinding procedure describing the workflow. This process will be approved by the central trial unit before study commencement. Data managers, statisticians and those writing the abstracts as well as drawing conclusions will also be blinded.

2.9 Participation criteria

All NICUs participating in the SafeBoosC-III trial will be invited to participate in the follow-up study. In the spring of 2021, NICUs will receive an official invitation to participate, along with this protocol, which has been approved by the Steering Committee. NICUs will be asked to state explicitly what data they will provide in the follow-up and as well as their expected follow-up percentage. NICUs will be encouraged to provide as much data as possible since this will strengthen the quality of the study.

The tasks for NICUs are as follows:

1. Obtain addendum ethical approval to conduct the follow-up study in their NICU, if necessary.
2. Obtain addendum contract and legal approval, if necessary
3. Delegate the review of health care records and outcome assessment to a co-investigator, who can be blinded to the group allocation in the SafeBoosC-III trial.
4. Develop local work flow and blinding procedure
5. Ensure that the contact information, ideally e-mail addresses of the parents, is maintained and that links to the web-based platform with the PARCA-R questionnaire and the health and development questionnaire are forwarded to the parents as necessary

Parents of all infants included in the SafeBoosC III trial, and alive at the time point where routine follow-up assessments are initiated, should be invited to participate in the follow-up study.

Since the first randomised infant in SafeBoosC III will reach two years of corrected age in September 2021, we expect that follow-up assessments will begin around this time point. The last follow-up assessment is expected to be completed before the end of 2024 since this is the time where we expect the last randomised infant to reach two years of corrected age plus six months of reserve.

2.10 Ethical considerations

2.10.1 Ethical review

The need for supplementary approval of the follow-up study by a Research Ethics Committee (REC) may differ among countries. In some countries, an REC approval may be an addition to the approval of the randomised clinical trial.

As the SafeBoosC-III follow-up study does not include exposures to any additional interventions, there is no safety risk for children. Thus, an interim analysis is not necessary.

2.10.2 Consent

When infants were randomised in the SafeBoosC-III trial, the parents were made aware of the possibility of a potential follow-up study and permission to contact families as foreseen in the information sheet the parents were given at inclusion. The study-related interventions consist of extracting data from health care records, and parental questionnaires. The need for explicit consent for using clinical data from health care records for the SafeBoosC-III follow-up study may differ between countries. In some countries, turning up for examination or answering questionnaires will be considered sufficient, implicit consent.

2.10.3 Transfer of data

Permissions for data collection and transfer to the centrial trial unit in Copenhagen must be obtained according to local/national regulations. If necessary, the contract between the partners must be extended.

2.11 Resources

The cost of the central trial unit is covered by the sponsor, while the costs of the NICUs is covered locally or nationally.

2.12 Data management plan

All the participating children's data are protected in accordance with the Danish Act on the processing of personal data and the Danish Health Act. Data entry will be managed through a web-based clinical trial software, OpenClinica, provided by Copenhagen Trial Unit. The electronic case report form has already been used as the platform for data entries in the SafeBoosC-III trial, using end-to-end encryption. For this follow-up study, an additional data entry module will be added to the SafeBoosC-III OpenClinica setup. Data will be stored in accordance with guidelines issued by the Danish Data Protection Agency, where the follow-up study will also seek approval from. Data are pseudonymised, only NICU numbers and study numbers are used to identify children. Personal identifying information linking to study numbers will be kept in the trial master file at the local NICU.

Six months after the acceptance of the main publication presenting the primary outcome, the dataset will be transferred to the Danish data archive, after re-coding of variables (birth weight, gestational age, sex, NICU, study number) that may be used for reidentification. This data set can be made

available for others as decided by the trial steering committee. Due to the residual risk of re-identification, the dataset will not be placed in public space.

3 Statistical analysis plan

Full details regarding statistical considerations and data analysis will be outlined in a separate report, which will be published before the analysis of the SafeBoosC-III trial data, expected late spring 2022.

Power estimations for the co-primary outcomes

The sample size of 1600 infants for the SafeBoosC-III trial is based on mortality and the prevalence of survivors with severe brain injury in the SafeBoosC II trial, i.e. a 22% relative risk reduction in the composite primary outcome from 34% to 26.5% (absolute risk reduction of 7,5%), at an alpha level of 5%, and a power of 90%. The latest Data Monitoring Report from March 2021 revealed a tentative mortality rate of 21% in the SafeBoosC-III trial, based on first 580 randomised infants. Assuming the mortality rate will stay constant, there will be 1264 infants alive at two years of corrected age, and thus, be available for follow-up assessment.

Death or moderate-or-severe neurodevelopmental disability power calculation

Based on answers from the questionnaire on systematic routine follow-up, as well as implementing parental questionnaires and informal assessments to classify neurodevelopment, we estimate the total sample size for the outcome death or moderate-or-severe NDD to 1600 children.
Based on results from two randomised controlled clinical trials (49,50) investigating neuroprotection in preterm infants, it is estimated that the proportion of children with the outcome death or moderate-or-severe NDD will be 50% in the control group. An indicative power calculation shows that if we want to test an absolute risk difference of eight percentage, between the experimental and control group, at an alfa of 2.5% and a sample size of 800 infants in each group, i.e. a total of 1600, we will reach a power of 80%.

Bayley III/IV mean cognitive score power calculation

Based on answers to a questionnaire on systematic routine follow-up from participating NICUs in the SafeBoosC-III consortium (see section 1.5 'recruitment and loss to follow up), it is expected that 65% of the NICUs will be able to provide data from a Bayley III/IV assessment around two years of corrected age for each child. Assuming that these NICUs enrol their proportion of the 1600 infants to the SafeBoosC-III trial, and a mortality rate of 21% (12), approximately 850 infants will be available for Bayley III/IV assessments at two years of age.

An indicative power estimation shows, that if we want to test a mean difference of five points on the Bayley cognitive score, with a standard deviation of 20 (Cohens d' 0.25) between the experimental and the control group, at a 2.5% alfa-level, with a sample size of 425 infants from each group in the follow-up study i.e. a total of 850, we will reach a power of 90%.

3.1 Primary analyses

The primary analysis of all outcomes will be based on the intention-to-treat population. Mixed-effect linear regression and mixed-effect logistic regression will be used to analyse the dichotomous and continuous co-primary outcomes, respectively. Data from all participating sites will be used in the primary analysis of both co-primary outcomes. In the regression models, 'site' will be included as a random effect, while 'gestational age below or above 26 weeks of postmenstrual age' and 'group allocation' will be included as fixed effects. To correct for multiple testing, the threshold for statistical significance will undergo Bonferroni adjustment, and thus a p-value of 0.025 for each primary outcome is chosen. Superiority of the intervention will only be claimed if at least one of the two co-primary outcomes is statistically significant. All other outcome results will be considered hypothesis-generating only. In addition, we will perform a range of pre-defined sensitivity analyses to inform the interpretation of the results of the primary analysis. A full statistical analysis plan will be developed and submitted for publication before the analysis of the SafeBoosC-III trial data.

3.2 Blinding of statisticians

All data managers, statisticians, and those drawing conclusions will be blinded to group allocation. Two blinded statisticians connected to The Copenhagen Trial Unit, will independently perform all statistical analyses and the two statistical reports will be published as supplemental material.

3.3 Publication plan

Once all data from follow-up assessments has been analysed, the results will be published in a peer-reviewed international journal. Members of the steering group will be offered authorship. Furthermore, one investigator per participating NICU can obtain co-authorship. All authors must fulfil the Vancouver Criteria. The blinded assessor completing the data entries will obtain non-byline co-authorship (51). Ancillary studies with results potentially affecting the main study or compromise its publication, shall not be published before the main results of the follow-up study have been published, as decided by the trial steering committee.

Appendix A: Prioritisation of data for the co-primary outcome moderate-or-severe NDD

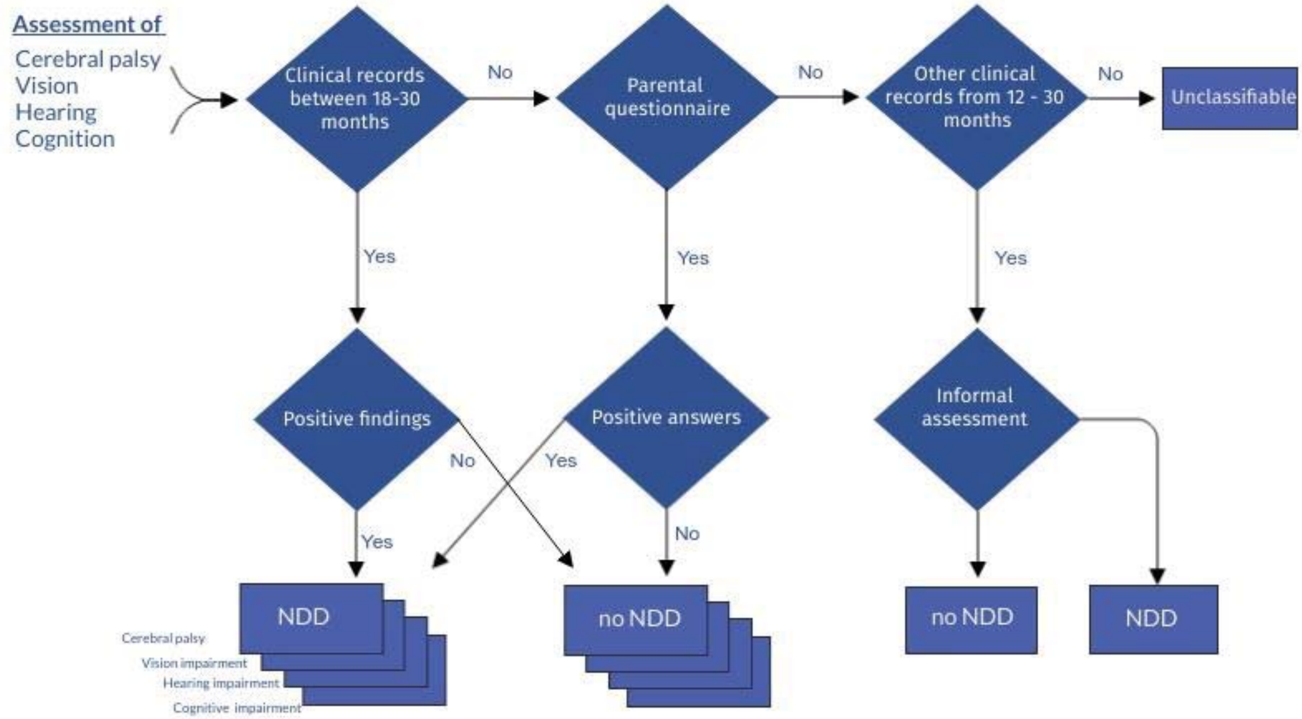

NDD = moderate-or-severe neurodevelopmental disability = positive findings for at least one of the four components <u>or</u> NDD based on an informal blinded assessment of all available information
No NDD = no moderate-or-severe neurodevelopmental disability = no positive findings in any of the four components and no NDD based on an informal blinded assessment of all available information
Unclassifiable = if one or more components are unknown <u>and</u> none are positive <u>and</u> there is no informal assessment.

Moderate-or-severe neurodevelopmental disability defined as:

Diagnosis of motor impairment: cerebral palsy with functional impairment corresponding to a GMFCS >=2, but it is not necessary that a GMFCS grading is available from the health care records)

Diagnosis of visual impairment: defined as moderate reduced vision or worse (blind in one eye with good vision in the contralateral eye or blind or can only perceive light or light reflecting objects).

Diagnosis of hearing impairment: defined as hearing loss corrected with aids or some hearing but loss not corrected by aids or no useful hearing even with aids.

Cognitive impairment:

- Bayley-III/IV cognitive score < 85 (1st priority)
- Any developmental assessment < -2SD (including the PARCA-R) (2nd priority)
- If none of the above are available, blinded assessment of health care records from 12 months of corrected age and onwards concluding that the child has a cognitive impairment equivalent to moderate or-severe nerudevelopmental disability (3rd priority).

Informal assessment of moderate-or-severe NDD

When there are no relevant health care records from corrected age 18-30 months the records will be accessed by a the blinded assessor and all health care records from corrected age 12 months and onwards will be informally assessed to conclude whether or not the child has moderate-or-severe neurodevelopmental disability.

Prioritisation and possible substitution of data will be done centrally.

Appendix B: Classification of NDD based on parental questionnaire (in cases where components of the co-primary outcomes are unknown or if it is not possible for the blinded assessor to assess NDD based on the health care records):

YES to cerebral palsy: A doctor has said the child has cerebral palsy
The child CANNOT walk independently at two years of age

NO to cerebral palsy: A doctor has said the child has cerebral palsy
The child CAN walk independently at two years of age

NO to cerebral palsy: A doctor has NOT said the child has cerebral palsy
The child CANNOT walk independently at two years of age

NO to cerebral palsy: A doctor has NOT said the child has cerebral palsy
The child CAN walk independently at two years of age

YES to visual impairment: The child is blind in one or two eyes OR has poor vision even with glasses

NO to visual impairment: The child has visual problems
The child wears glasses
The child has a squint (the eyes look in different directions)

YES to hearing impairment: The child wears aids or cochlear implants

The child will be categorised as no moderate-or-severe NDD if all components are ”no”.
The child will be categorised as “not able to classify”, if one or more components are unknown and none are positive. Thereby all four components of the primary outcome must be “no”, to be classified as no moderate-or-severe NDD.
Example:
From health care records: no to cerebral palsy
From parental questionnaire: no vision impairment,

No clinical record or parental information of hearing impairment or standardised developmental assessment or PARCA-R score.

The child will be "not able to classify"

Appendix C: ancillary studies

The SafeBoosC-III two-year follow-up study will provide important data on the long-term consequences of extreme prematurity. Therefore, we are therefore drafting multiple secondary analyses on data from the SafeBoosC-III trial and follow-up study.

Predictive value of neonatal cerebral ultrasound at 36 weeks postmenstrual age in relation to neurodevelopmental impairment at 24 months of corrected age in extremely preterm infants

In the SafeBoosC-III trial, the primary outcome is a composite of death or severe brain injury. One of the co-primary outcomes of the SafeBoosC-III follow-up study is death or moderate-to-severe neurodevelopmental disability at two years of corrected age. For the purpose of this secondary analysis, the correlation between the brain injury diagnoses included in the 'severe brain injury' criteria at 36 weeks PMA and the diagnosis of moderate-to-severe neurodevelopmental impairment at two years of age will be investigated. For the brain injuries cerebral haemorrhage grade III or IV, cystic periventricular leukomalacia, cerebellar haemorrhage, post-haemorrhagic ventricular dilatation or cerebral atrophy, each injury will be computed for the proportion of children with 1) CP (GMFCS ≥ 2) a Bayley III/IV cognitive score below 85, and 3) moderate-or-severe neurodevelopmental disability. To describe the correlation between brain injury diagnosed during the neonatal period and the outcomes, risk ratios will be calculated adjusted for gestational age as well as 95% confidence intervals.

Two-year outcomes of extremely preterm infants: mechanically ventilated vs non-ventilated

In neonates with respiratory failure, mechanical ventilation is a life-saving intervention. It is often the sickest neonates that are requiring mechanical ventilation, whom are frequently suffering from circulatory failure as well. The combination of circulatory and respiratory failure, on top of an increased risk of in advert hyperventilation due to the mechanical ventilation, puts this group of neonates in increased risk of cerebral hypoxia (49). Thus, we hypothesise that these neonates would have the largest effect of clinical care with access to cerebral NIRS monitoring. In order to test this hypothesis, we plan to conduct a secondary analysis, comparing children in the experimental group that underwent mechanical ventilation, to children in the experimental group that did not undergo mechanical ventilation and children in the control group, in regard to the two co-primary outcomes of the SafeBoosC-III trial follow-up, as well as the interactions with the group allocation. The

hypothesis is that the effect of the SafeBoosC-III intervention would greater in ventilated compared to non-ventilated infants.

Correlation between Bayley III/IV and PARCA-R

The PARCA-R questionnaire has previously shown a good correlation to Bayley II and Bayley III scores in national studies (36). However, the literature is sparse when investigating the correlation in an international trial setting using results from routine Bayley tests. The SafeBoosC-III follow-up study plans to make PARCA-R available to the parents of all infants included in the SafeBoosC-III trial. Furthermore, at least 36 NICU will use the Bayley III/IV assessment as an outcome measure. We therefore plan to compare and calculate the correlation between Bayley III/IV scores and PARCA-R scores. The Bayley III/IV cognitive score will be used as a gold standard to assess concurrent validity of the NVC of the PARCA-R. The Pearson correlation coefficient (Pearson's r) will be calculated to assess the relationship between the two continuous measures. Two receiver operating characteristic curves (ROC) will be plotted to investigate the diagnostic accuracy of NVC scores to predict Bayley III/IV cognitive scores below 85, which will be defined as moderate-to-severe neurodevelopmental impairment. The accuracy of the NVC in detecting values below 85 and 70 on the Bayley cognitive scale will be computed by the area under curve measurements.

Standardised mean differences of all assessments used in the SafeBoosC-III follow-up

Since the SafeBoosC-III follow-up study utilises many different assessments, a comparison may be complex. The Bayley III/IV measures differently from the Griffiths, however, the standardised mean difference (SMD) allows for outcomes of similar nature but measured on different scales, to be combined. The SMD will express the possible intervention effect relative to the variability between the developmental tests. This is a commonly used method in meta-analyses. The SMD is used interchangeable with the term "effect size", and is furthermore often used in relation to Cohen's d. The standardisation is obtained by dividing the difference in mean outcome between two groups, with the pooled standard deviation of the measurements. We believe this will provide a valuable point estimate of the effect of the intervention. For the purpose of this analysis, all quantitative scores derived from standardised developmental assessments at two years of corrected age, will be pooled for the statistical analyses and test values will be converted to SMD and will thus allow for comparison between the control and experimental group. All analyses will be intention to treat analyses.

References

1. Blencowe, H., S. Cousens, M.Z. Oestergaard, et al., National, regional, and worldwide estimates of preterm birth rates in the year 2010 with time trends since 1990 for selected countries: a systematic analysis and implications, *The Lancet (British edition)*, 2012. **379**(9832): p. 2162-2172.

2. Cheong, J.L.Y., P.J. Anderson, A.C. Burnett, et al., Changing Neurodevelopment at 8 Years in Children Born Extremely Preterm Since the 1990s, *Pediatrics*, 2017. **139**(6).

3. Norman, M., B. Hallberg, T. Abrahamsson, et al., Association Between Year of Birth and 1-Year Survival Among Extremely Preterm Infants in Sweden During 2004-2007 and 2014-2016, *JAMA*, 2019. **321**(12): p. 1188-1199.

4. Stoll, B.J., N.I. Hansen, E.F. Bell, et al., Trends in Care Practices, Morbidity, and Mortality of Extremely Preterm Neonates, 1993-2012, *JAMA*, 2015. **314**(10): p. 1039-51.

5. Costeloe, K.L., E.M. Hennessy, S. Haider, et al., Short term outcomes after extreme preterm birth in England: comparison of two birth cohorts in 1995 and 2006 (the EPICure studies), *BMJ*, 2012. **345**: p. e7976.

6. Stoll, B.J., N.I. Hansen, E.F. Bell, et al., Neonatal outcomes of extremely preterm infants from the NICHD Neonatal Research Network, *Pediatrics*, 2010. **126**(3): p. 443-56.

7. Volpe, J.J., Brain injury in the premature infant. Neuropathology, clinical aspects, pathogenesis, and prevention, *Clin Perinatol*, 1997. **24**(3): p. 567-87.

8. Marret, S., L. Marchand-Martin, J.C. Picaud, et al., Brain injury in very preterm children and neurosensory and cognitive disabilities during childhood: the EPIPAGE cohort study, *PLoS One*, 2013. **8**(5): p. e62683.

9. Volpe, J.J., Brain injury in premature infants: a complex amalgam of destructive and developmental disturbances, *Lancet Neurol*, 2009. **8**(1): p. 110-24.

10. Saigal, S., K. Morrison, and L.A. Schmidt, "Health, wealth and achievements of former very premature infants in adult life", *Semin Fetal Neonatal Med*, 2020. **25**(3): p. 101107.

11. Pellicer, A., G. Greisen, M. Benders, et al., The SafeBoosC phase II randomised clinical trial: a treatment guideline for targeted near-infrared-derived cerebral tissue oxygenation versus standard treatment in extremely preterm infants, *Neonatology*, 2013. **104**(3): p. 171-8.

12. Plomgaard, A.M., T. Alderliesten, F. van Bel, et al., No neurodevelopmental benefit of cerebral oximetry in the first randomised trial (SafeBoosC II) in preterm infants during the first days of life, *Acta Paediatr*, 2019. **108**(2): p. 275-281.

13. Hyttel-Sorensen, S., A. Pellicer, T. Alderliesten, et al., Cerebral near infrared spectroscopy oximetry in extremely preterm infants: phase II randomised clinical trial, *BMJ*, 2015. **350**: p. g7635.

14. Akobeng, A.K., Understanding type I and type II errors, statistical power and sample size, *Acta Paediatr*, 2016. **105**(6): p. 605-9.

15. Hansen, M.L., A. Pellicer, C. Gluud, et al., Cerebral near-infrared spectroscopy monitoring versus treatment as usual for extremely preterm infants: a protocol for the SafeBoosC randomised clinical phase III trial, *Trials*, 2019. **20**(1): p. 811.

16. Hansen, M.L., A. Pellicer, C. Gluud, et al., Detailed statistical analysis plan for the SafeBoosC III trial: a multinational randomised clinical trial assessing treatment guided by cerebral oxygenation monitoring versus treatment as usual in extremely preterm infants, *Trials*, 2019. **20**(1): p. 746.

17. Dudink, J., S. Jeanne Steggerda, S. Horsch, et al., State-of-the-art neonatal cerebral ultrasound: technique and reporting, *Pediatr Res*, 2020. **87**(Suppl 1): p. 3-12.

18. Patel, R.M., Short- and Long-Term Outcomes for Extremely Preterm Infants, *Am J Perinatol*, 2016. **33**(3): p. 318-28.

19. O'Shea, T.M., K.C. Kuban, E.N. Allred, et al., Neonatal cranial ultrasound lesions and developmental delays at 2 years of age among extremely low gestational age children, *Pediatrics*, 2008. **122**(3): p. e662-9.

20. Inder, T.E., N.J. Anderson, C. Spencer, et al., White matter injury in the premature infant: a comparison between serial cranial sonographic and MR findings at term, *AJNR Am J Neuroradiol*, 2003. **24**(5): p. 805-9.

21. Leijser, L.M., F.T. de Bruine, J. van der Grond, et al., Is sequential cranial ultrasound reliable for detection of white matter injury in very preterm infants?, *Neuroradiology*, 2010. **52**(5): p. 397-406.

22. Marlow, N., Is survival and neurodevelopmental impairment at 2 years of age the gold standard outcome for neonatal studies?, *Arch Dis Child Fetal Neonatal Ed*, 2015. **100**(1): p. F82-4.

23. Hack, M., H.G. Taylor, D. Drotar, et al., Poor predictive validity of the Bayley Scales of Infant Development for cognitive function of extremely low birth weight children at school age, *Pediatrics*, 2005. **116**(2): p. 333-41.

24. Sutcliffe, A.G., A. Soo, and J. Barnes, Predictive value of developmental testing in the second year for cognitive development at five years of age, *Pediatr Rep*, 2010. **2**(2): p. e15.

25. Marlow, N., Measuring neurodevelopmental outcome in neonatal trials: a continuing and increasing challenge, *Arch Dis Child Fetal Neonatal Ed*, 2013. **98**(6): p. F554-8.

26. Johnson, S., D. Wolke, N. Marlow, et al., Developmental assessment of preterm infants at 2 years: validity of parent reports, *Dev Med Child Neurol*, 2008. **50**(1): p. 58-62.

27. Johnson, S., V. Bountziouka, P. Brocklehurst, et al., Standardisation of the Parent Report of Children's Abilities-Revised (PARCA-R): a norm-referenced assessment of cognitive and language development at age 2 years, *Lancet Child Adolesc Health*, 2019. **3**(10): p. 705-712.

28. Blaggan, S., A. Guy, E.M. Boyle, et al., A parent questionnaire for developmental screening in infants born late and moderately preterm, *Pediatrics*, 2014. **134**(1): p. e55-62.

29. Group, I.S.C., The INIS Study. International Neonatal Immunotherapy Study: non-specific intravenous immunoglobulin therapy for suspected or proven neonatal sepsis: an international, placebo controlled, multicentre randomised trial, *BMC Pregnancy Childbirth*, 2008. **8**: p. 52.

30. Onland, W., M.P. Merkus, D.H. Nuytemans, et al., Systemic Hydrocortisone To Prevent Bronchopulmonary Dysplasia in preterm infants (the SToP-BPD study): statistical analysis plan, *Trials*, 2018. **19**(1): p. 178.

31. Doyle, L.W., J.L. Cheong, R.A. Ehrenkranz, et al., Early (< 8 days) systemic postnatal corticosteroids for prevention of bronchopulmonary dysplasia in preterm infants, *Cochrane Database Syst Rev*, 2017. **10**: p. CD001146.

32. Lindsey, J.C. and P. Brouwers, Intrapolation and extrapolation of age-equivalent scores for the Bayley II: a comparison of two methods of estimation, *Clin Neuropharmacol*, 1999. **22**(1): p. 44-53.

33. Lowe, J.R., S.J. Erickson, R. Schrader, et al., Comparison of the Bayley II Mental Developmental Index and the Bayley III Cognitive Scale: are we measuring the same thing?, *Acta Paediatr*, 2012. **101**(2): p. e55-8.

34. Moore, T., S. Johnson, S. Haider, et al., Relationship between test scores using the second and third editions of the Bayley Scales in extremely preterm children, *J Pediatr*, 2012. **160**(4): p. 553-8.

35. Johnson, S., T. Moore, and N. Marlow, Using the Bayley-III to assess neurodevelopmental delay: which cut-off should be used?, *Pediatr Res*, 2014. **75**(5): p. 670-4.

36. Martin, A.J., B.A. Darlow, A. Salt, et al., Performance of the Parent Report of Children's Abilities-Revised (PARCA-R) versus the Bayley Scales of Infant Development III, *Arch Dis Child*, 2013. **98**(12): p. 955-8.

37. Johnson, S., N. Marlow, D. Wolke, et al., Validation of a parent report measure of cognitive development in very preterm infants, *Dev Med Child Neurol*, 2004. **46**(6): p. 389-97.

38. Picotti, E., N. Bechtel, B. Latal, et al., Performance of the German version of the PARCA-R questionnaire as a developmental screening tool in two-year-old very preterm infants, *PLoS One*, 2020. **15**(9): p. e0236289.

39. Johnson, S., T.A. Evans, E.S. Draper, et al., Neurodevelopmental outcomes following late and moderate prematurity: a population-based cohort study, *Arch Dis Child Fetal Neonatal Ed*, 2015. **100**(4): p. F301-8.

40. Paulson, A. and J. Vargus-Adams, Overview of Four Functional Classification Systems Commonly Used in Cerebral Palsy, *Children (Basel)*, 2017. **4**(4).

41. Marlow, N., L.W. Doyle, P. Anderson, et al., Assessment of long-term neurodevelopmental outcome following trials of medicinal products in newborn infants, *Pediatr Res*, 2019. **86**(5): p. 567-572.

42. Folsom, R.C., J.E. Widen, B.R. Vohr, et al., Identification of neonatal hearing impairment: recruitment and follow-up, *Ear Hear*, 2000. **21**(5): p. 462-70.

43. Howe, C.J., S.R. Cole, B. Lau, et al., Selection Bias Due to Loss to Follow Up in Cohort Studies, *Epidemiology*, 2016. **27**(1): p. 91-7.

44. Jakobsen, J.C., J. Wetterslev, P. Winkel, et al., Thresholds for statistical and clinical significance in systematic reviews with meta-analytic methods, *BMC Med Res Methodol*, 2014. **14**: p. 120.

45. Goodman, S.N., Introduction to Bayesian methods I: measuring the strength of evidence, *Clin Trials*, 2005. **2**(4): p. 282-90; discussion 301-4, 364-78.

46. Jakobsen, J.C., C. Gluud, J. Wetterslev, et al., When and how should multiple imputation be used for handling missing data in randomised clinical trials - a practical guide with flowcharts, *BMC Med Res Methodol*, 2017. **17**(1): p. 162.

47. Hyttel-Sorensen, S., T. Austin, F. van Bel, et al., A phase II randomized clinical trial on cerebral near-infrared spectroscopy plus a treatment guideline versus treatment as usual for extremely preterm infants during the first three days of life (SafeBoosC): study protocol for a randomized controlled trial, *Trials*, 2013. **14**: p. 120.

48. Gates, S. and P. Brocklehurst, How should randomised trials including multiple pregnancies be analysed?, *BJOG*, 2004. **111**(3): p. 213-9.

49. Natalucci G, Latal B, Koller B, et al. Effect of Early Prophylactic High-Dose Recombinant Human Erythropoietin in Very Preterm Infants on Neurodevelopmental Outcome at 2 Years: A Randomized Clinical Trial. *JAMA.* 2016;315(19):2079–2085.

50. Juul SE, Comstock BA, Wadhawan R, et al. A Randomized Trial of Erythropoietin for Neuroprotection in Preterm Infants. *N Engl J Med*. 2020;382(3):233-243.

51. Fontanarosa P, Bauchner H, Flanagin A. Authorship and Team Science. *JAMA.* 2017;318(24):2433–2437.

52. Aylward, G., & Zhu, J. (2019). The Bayley Scales: Clarification for Clinicians and Researchers.
