# Supplementary material for: Cerebral Oximetry in Extremely Preterm Infants: 2-Year Follow-Up of the SafeBoosC-III Randomized Clinical Trial
Source: JAMA Pediatr. 2026 Apr 20;180(6):619–27. doi: 10.1001/jamapediatrics.2026.1066 (PMC13097032; doi:10.1001/jamapediatrics.2026.1066)
Supplement: Supplement 2. — Statistical Analysis Plan. [file jamapediatr-e261066-s002.pdf]

Statistical report for an SafeBoosC-III Follow-up study

Markus Harboe Olsen

2025-01-28

Table of contents

Table 1 3

Table 2 - Distribution of data sources 4

Table 3 - Outcomes at 2 years of corrected age 5

Supplemental 7

- Follow up times 7
- Alive participants for dichotomous outcomes 8
- Without missingness 9
  - Table 1 without missingness 9
  - Table 2 without missingness 10
  - Table 3 without missingness 11
  - Alive participants 12
- Sensitivity analysis 13
  - Per-protocol 13
  - Not filtered for death 14
  - Without informal assessment 14
  - Best-Worst Worst-Best - Death or moderate-or-severe NDD 15
  - Multiple imputations - Death or moderate-or-severe NDD 16
  - High and low follow-up rates 18
  - Random-effects meta-analysis 19
  - Generalised estimating equation 23
  - Skewness of missingness 26
  - All PARCAR scores and non-parametric for PARCAR scores 26

Added after statistical report 27
Histograms of continuous outcomes . . . 27
Per-protocol . . . 29
ROP . . . 29
SD for continuous outcomes . . . 29
Additional follow-up times . . . 29
Treatment specific variables . . . 30
ICC . . . 31
Death or moderate-or-severe NDD . . . 31
Bayley cognitive III/IV score . . . 31
PARCAR additional analyses . . . 32
Primary outcome with extrapolated data . . . 32
Grouping . . . 32
Who saw (only alive) . . . 33
Death or moderate-to-severe NDD - Tier 3 outcome . . . 33
Cognitive impairment - Where Cognitive impairment other than bailey is responsible for change primary outcome from no to yes . . . 34
Lost to follow-up . . . 35

Table 1

	**Control group** *n = 741*	**Experimental group** *n = 697*
**Baseline characteristics data**		
Birth weight (g)		
median [IQR]	800.00 [660.00;950.00]	800 [660.00;958.00]
mean (95%CI)	812.58 (797.61;827.54)	816.20 (801.39;831.01)
missing	1 (0.1%)	0 (0%)
Gestational age >26 weeks	409 (55.2%)	381 (54.7%)
Gestational age (weeks)		
median [IQR]	26.14 [25.00;27.14]	26.14 [25.00;27.14]
mean (95%CI)	25.98 (25.88;26.07)	26.01 (25.91;26.10)
Twins or triplets	219 (29.6%)	168 (24.1%)
missing	0 (0.0%)	3 (0.4%)
Male	383 (51.7%)	375 (53.8%)
Apgar at 5 min.		
median [IQR]	7.00 [6.00;8.00]	7.00 [6.00;8.00]
mean (95%CI)	5.92 (5.11;6.72)	6.37 (5.69;7.06)
missing	1 (0.1%)	3 (0.4%)
**Clinical neonatal characteristics**		
Major congenital anomaly	17 (2.3%)	15 (2.2%)
Cardiovascular support within 72 hours	227 (30.6%)	256 (36.7%)
Mechanical ventilation	577 (77.9%)	551 (79.1%)
Mechanical ventilation (days)		
median [IQR]	9 [3.00;25.00]	9 [3.00;23.00]
mean (95%CI)	17.04 (15.53;18.55)	16.45 (14.95;17.95)
missing	164 (22.1%)	146 (20.9%)
Retinopathy of prematurity	88 (11.9%)	97 (13.9%)
Necrotising enterocolitis	82 (11.1%)	87 (12.5%)
Late onset sepsis	470 (63.4%)	425 (61.0%)
Bronchopulmonal dysplasia	340 (45.9%)	287 (41.2%)
missing	169 (22.8%)	173 (24.8%)
Severe brain injury at 36 weeks PMA	170 (22.9%)	171 (24.5%)
missing	9 (1.2%)	9 (1.3%)
Death before 36 weeks PMA	147 (19.8%)	150 (21.5%)

Table 2 - Distribution of data sources

	**Overall** *n = 1438*
Death or moderate-or-severe NDD (Tier)	
Tier 1	1002 (69.7%)
Tier 2	154 (10.7%)
Tier 3	133 (9.2%)
missing	149 (10.4%)
Cerebral palsy (Tier)	
Tier 1	1186 (82.5%)
Tier 2	89 (6.2%)
missing	163 (11.3%)
Visual impairment (Tier)	
Tier 1	1185 (82.4%)
Tier 2	88 (6.1%)
missing	165 (11.5%)
Hearing impairment (Tier)	
Tier 1	1184 (82.3%)
Tier 2	91 (6.3%)
missing	163 (11.3%)
Cognitive impairment (Tier)	
Tier 1	967 (67.2%)
Tier 2	165 (11.5%)
Tier 3	124 (8.6%)
missing	182 (12.7%)

Table 3 - Outcomes at 2 years of corrected age

	**Control group** *n = 741*	**Experimental group** *n = 697*	**Logistic regression** *RR (97.5%CI)*	*p*
**Co-primary outcomes**				
Death or moderate-or-severe NDD	321 (43.3%)	292 (41.9%)	0.96 (0.85;1.07)	0.449
missing	72 (9.7%)	77 (11.0%)		

	**Control group** *n = 741*	**Experimental group** *n = 697*	**Mixed effects linear regression** *estimate (97.5%CI)*	*p*
**Co-primary outcomes**				
Bayley cognitive III/IV score			-0.14 (-3.24;2.96)	0.922
median [IQR]	95.00 [80.00;105.00]	95.00 [85.00;100.00]		
mean (97.5%CI)	93.18 (90.87;95.49)	92.83 (90.38;95.27)		
missing	457 (61.7%)	451 (64.7%)		

	**Control group** *n = 741*	**Experimental group** *n = 697*	**Logistic regression** *RR (95%CI)*	*p*
**Components of the dichotomous outcome**				
Death	165 (22.3%)	162 (23.2%)	1.01 (0.83;1.21)	0.954
missing	70 (9.4%)	72 (10.3%)		
Death or Cerebral palsy	187 (25.2%)	187 (26.8%)	1.03 (0.87;1.22)	0.740
missing	81 (10.9%)	82 (11.8%)		
Death or Visual impairment	208 (28.1%)	187 (26.8%)	0.91 (0.77;1.08)	0.288
missing	83 (11.2%)	82 (11.8%)		
Death or Hearing impairment	179 (24.2%)	178 (25.5%)	1.03 (0.86;1.23)	0.759
missing	79 (10.7%)	84 (12.1%)		
Death or Cognitive impairment	281 (37.9%)	268 (38.5%)	0.99 (0.87;1.13)	0.910
missing	91 (12.3%)	91 (13.1%)		
**Exploratory outcomes**				
Death or Any other chronic illness	319 (43.0%)	279 (40.0%)	0.91 (0.81;1.03)	0.127
missing	83 (11.2%)	84 (12.1%)		
Death or Any daily medication for the last two months	273 (36.8%)	255 (36.6%)	0.97 (0.85;1.11)	0.657
missing	88 (11.9%)	84 (12.1%)		
Death or Hospitalisation since discharge from index hospitalisation	351 (47.4%)	343 (49.2%)	1.03 (0.94;1.13)	0.485
missing	195 (26.3%)	186 (26.7%)		
Death or Parental report of thriving child	533 (71.9%)	499 (71.6%)	1.00 (0.98;1.02)	0.939
missing	195 (26.3%)	187 (26.8%)		
Death or Parental report of worries regarding the child	309 (41.7%)	286 (41.0%)	0.98 (0.88;1.09)	0.701
missing	202 (27.3%)	194 (27.8%)		

	**Control group** *n = 741*	**Experimental group** *n = 697*	**Mixed effects linear regression** *estimate (95%CI)*	*p*
**Exploratory outcomes**				
Head circumference (cm)			0.03 (-0.27;0.34)	0.822

	**Control group** *n = 741*	**Experimental group** *n = 697*	**Mixed effects linear regression** *estimate (95%CI)*	*p*
median [IQR]	47.00 [46.00;49.00]	47.50 [46.00;49.00]		
mean (95%CI)	47.31 (47.11;47.51)	47.32 (47.07;47.57)		
missing	320 (43.2%)	324 (46.5%)		
Height (cm)			-0.20 (-0.87;0.46)	0.554
median [IQR]	85.00 [82.00;88.00]	85.00 [82.00;88.00]		
mean (95%CI)	84.90 (84.45;85.34)	84.57 (84.01;85.12)		
missing	318 (42.9%)	313 (44.9%)		
Body weight (kg)			-0.10 (-0.35;0.14)	0.398
median [IQR]	11.10 [10.00;12.30]	11.10 [10.00;12.00]		
mean (95%CI)	11.28 (11.11;11.45)	11.17 (11.00;11.35)		
missing	302 (40.8%)	302 (43.3%)		
PARCAR score			-0.40 (-3.81;2.98)	0.817
median [IQR]	89.00 [76.00;99.50]	88.00 [76.25;99.00]		
mean (95%CI)	87.42 (84.86;89.97)	86.44 (83.84;89.04)		
missing	473 (63.8%)	439 (63%)		

Supplemental

Follow up times

	**Control group** *n = 741*	**Experimental group** *n = 697*
PARCAR follow-up (corrected, months)		
median [IQR]	25.69 [24.42;27.77]	25.53 [24.47;27.36]
mean (95%CI)	26.67 (26.35;26.99)	26.46 (26.13;26.79)
missing	354 (47.8%)	343 (49.2%)
Bayley follow-up (corrected, months)		
median [IQR]	24.47 [23.83;25.26]	24.52 [23.69;25.54]
mean (95%CI)	24.47 (24.16;24.79)	24.57 (24.23;24.92)
missing	457 (61.7%)	451 (64.7%)
Neuropediatrician follow-up (corrected, months)		
median [IQR]	24.35 [23.44;25.32]	24.17 [22.96;25.33]
mean (95%CI)	24.39 (23.82;24.97)	23.78 (23.04;24.53)
missing	613 (82.7%)	581 (83.4%)
Pediatrician/neonatologist follow-up (corrected, months)		
median [IQR]	24.33 [23.23;25.58]	24.35 [22.74;25.58]
mean (95%CI)	24.19 (23.91;24.46)	23.90 (23.58;24.23)
missing	351 (47.4%)	356 (51.1%)
Physiotherapist (corrected, months)		
median [IQR]	24.24 [23.59;25.35]	24.04 [20.83;24.83]
mean (95%CI)	23.71 (22.92;24.50)	22.51 (21.64;23.37)
missing	639 (86.2%)	604 (86.7%)
Psychologist (corrected, months)		
median [IQR]	24.55 [23.84;25.71]	24.48 [23.84;25.20]
mean (95%CI)	24.78 (24.47;25.10)	24.44 (24.05;24.83)
missing	549 (74.1%)	532 (76.3%)

Alive participants for dichotomous outcomes

	Control group *n = 506*	Experimental group *n = 463*
**Co-primary outcomes**		
Death or moderate-or-severe NDD	156 (30.8%)	130 (28.1%)
missing	2 (0.4%)	5 (1.1%)
**Components of the dichotomous outcome**		
Death - No	506 (100.0%)	463 (100.0%)
Death or Cerebral palsy	22 (4.3%)	25 (5.4%)
missing	11 (2.2%)	10 (2.2%)
Death or Visual impairment	43 (8.5%)	25 (5.4%)
missing	13 (2.6%)	10 (2.2%)
Death or Hearing impairment	14 (2.8%)	16 (3.5%)
missing	9 (1.8%)	12 (2.6%)
Death or Cognitive impairment	116 (22.9%)	106 (22.9%)
missing	21 (4.2%)	19 (4.1%)
**Exploratory outcomes**		
Death or Any other chronic illness	154 (30.4%)	117 (25.3%)
missing	13 (2.6%)	12 (2.6%)
Death or Any daily medication for the last two months	108 (21.3%)	93 (20.1%)
missing	18 (3.6%)	12 (2.6%)
Death or Hospitalisation since discharge from index hospitalisation	186 (36.8%)	181 (39.1%)
missing	125 (24.7%)	114 (24.6%)
Death or Parental report of thriving child	368 (72.7%)	337 (72.8%)
missing	125 (24.7%)	115 (24.8%)
Death or Parental report of worries regarding the child	144 (28.5%)	124 (26.8%)
missing	132 (26.1%)	122 (26.3%)

Without missingness

Table 1 without missingness

	**Control group** *n = 741*	**Experimental group** *n = 697*
**Baseline characteristics data**		
Birth weight (g)		
median [IQR]	800.00 [660.00;950.00]	800 [660.00;958.00]
mean (95%CI)	812.58 (797.61;827.54)	816.20 (801.39;831.01)
Gestational age >26 weeks	409 (55.2%)	381 (54.7%)
Gestational age (weeks)		
median [IQR]	26.14 [25.00;27.14]	26.14 [25.00;27.14]
mean (95%CI)	25.98 (25.88;26.07)	26.01 (25.91;26.10)
Twins or triplets	219 (29.6%)	168 (24.2%)
Male	383 (51.7%)	375 (53.8%)
Apgar at 5 min.		
median [IQR]	7.00 [6.00;8.00]	7.00 [6.00;8.00]
mean (95%CI)	5.92 (5.11;6.72)	6.37 (5.69;7.06)
**Clinical neonatal characteristics**		
Major congenital anomaly	17 (2.3%)	15 (2.2%)
Cardiovascular support within 72 hours	227 (30.6%)	256 (36.7%)
Mechanical ventilation	577 (77.9%)	551 (79.1%)
Mechanical ventilation (days)		
median [IQR]	9 [3.00;25.00]	9 [3.00;23.00]
mean (95%CI)	17.04 (15.53;18.55)	16.45 (14.95;17.95)
Retinopathy of prematurity	88 (11.9%)	97 (13.9%)
Necrotising enterocolitis	82 (11.1%)	87 (12.5%)
Late onset sepsis	470 (63.4%)	425 (61.0%)
Bronchopulmonal dysplasia	340 (59.4%)	287 (54.8%)
Severe brain injury at 36 weeks PMA	170 (23.2%)	171 (24.9%)
Death before 36 weeks PMA	147 (19.8%)	150 (21.5%)

Table 2 without missingness

	**Overall**<br>*n = 1438*
Death or moderate-or-severe NDD (Tier)	
Tier 1	1002 (77.7%)
Tier 2	154 (11.9%)
Tier 3	133 (10.3%)
Cerebral palsy (Tier)	
Tier 1	1186 (93.0%)
Tier 2	89 (7.0%)
Visual impairment (Tier)	
Tier 1	1185 (93.1%)
Tier 2	88 (6.9%)
Hearing impairment (Tier)	
Tier 1	1184 (92.9%)
Tier 2	91 (7.1%)
Cognitive impairment (Tier)	
Tier 1	967 (77.0%)
Tier 2	165 (13.1%)
Tier 3	124 (9.9%)

Table 3 without missingness

	Control group<br>*n = 741*	Experimental group<br>*n = 697*
**Co-primary outcomes**		
Death or moderate-or-severe NDD		
Yes	321 (48.0%)	292 (47.1%)
Bayley cognitive III/IV score		
median [IQR]	95.00 [80.00;105.00]	95.00 [85.00;100.00]
mean (97.5%CI)	93.18 (90.87;95.49)	92.83 (90.38;95.27)

	Control group<br>*n = 741*	Experimental group<br>*n = 697*
**Components of the dichotomous outcome**		
Death	165 (24.6%)	162 (25.9%)
Death or Cerebral palsy	187 (28.3%)	187 (30.4%)
Death or Visual impairment	208 (31.6%)	187 (30.4%)
Death or Hearing impairment	179 (27.0%)	178 (29.0%)
Death or Cognitive impairment	281 (43.2%)	268 (44.2%)
**Exploratory outcomes**		
Death or Any other chronic illness	319 (48.5%)	279 (45.5%)
Death or Any daily medication for the last two months	273 (41.8%)	255 (41.6%)
Death or Hospitalisation since discharge from index hospitalisation	351 (64.3%)	343 (67.1%)
Death or Parental report of thriving child	533 (97.6%)	499 (97.8%)
Death or Parental report of worries regarding the child		
Yes	309 (57.3%)	286 (56.9%)
Head circumference (cm)		
median [IQR]	47.00 [46.00;49.00]	47.50 [46.00;49.00]
mean (95%CI)	47.31 (47.11;47.51)	47.32 (47.07;47.57)
Height (cm)		
median [IQR]	85.00 [82.00;88.00]	85.00 [82.00;88.00]
mean (95%CI)	84.90 (84.45;85.34)	84.57 (84.01;85.12)
Body weight (kg)		
median [IQR]	11.10 [10.00;12.30]	11.10 [10.00;12.00]
mean (95%CI)	11.28 (11.11;11.45)	11.17 (11.00;11.35)
PARCAR score		
median [IQR]	89.00 [76.00;99.50]	88.00 [76.25;99.00]
mean (95%CI)	87.42 (84.86;89.97)	86.44 (83.84;89.04)

Alive participants

	Control group<br>*n = 506*	Experimental group<br>*n = 463*
**Co-primary outcomes**		
Death or moderate-or-severe NDD		
Yes	156 (31.0%)	130 (28.4%)
Bayley cognitive III/IV score		
median [IQR]	95.00 [80.00;105.00]	95.00 [85.00;100.00]
mean (97.5%CI)	93.18 (90.87;95.49)	92.83 (90.38;95.27)

	Control group<br>*n = 506*	Experimental group<br>*n = 463*
**Components of the dichotomous outcome**		
Death - No	506 (100.0%)	463 (100.0%)
Death or Cerebral palsy	22 (4.4%)	25 (5.5%)
Death or Visual impairment	43 (8.7%)	25 (5.5%)
Death or Hearing impairment	14 (2.8%)	16 (3.5%)
Death or Cognitive impairment	116 (23.9%)	106 (23.9%)
**Exploratory outcomes**		
Death or Any other chronic illness	154 (31.2%)	117 (25.9%)
Death or Any daily medication for the last two months	108 (22.1%)	93 (20.6%)
Death or Hospitalisation since discharge from index hospitalisation	186 (48.8%)	181 (51.9%)
Death or Parental report of thriving child	368 (96.6%)	337 (96.8%)
Death or Parental report of worries regarding the child		
Yes	144 (38.5%)	124 (36.4%)
Head circumference (cm)		
median [IQR]	47.00 [46.00;49.00]	47.50 [46.00;49.00]
mean (95%CI)	47.31 (47.11;47.51)	47.32 (47.07;47.57)
Height (cm)		
median [IQR]	85.00 [82.00;88.00]	85.00 [82.00;88.00]
mean (95%CI)	84.90 (84.45;85.34)	84.57 (84.01;85.12)
Body weight (kg)		
median [IQR]	11.10 [10.00;12.30]	11.10 [10.00;12.00]
mean (95%CI)	11.28 (11.11;11.45)	11.17 (11.00;11.35)
PARCAR score		
median [IQR]	89.00 [76.00;99.50]	88.00 [76.25;99.00]
mean (95%CI)	87.42 (84.86;89.97)	86.44 (83.84;89.04)

Sensitivity analysis

Per-protocol

	Control group *n = 727*	Experimental group *n = 665*	Logistic regression *RR (97.5%CI)*	*p*
Death or moderate-or-severe NDD	313 (43.1%)	284 (42.7%)	0.97 (0.86;1.10)	0.645
missing	72 (9.9%)	75 (11.3%)		

	Control group *n = 727*	Experimental group *n = 665*	Mixed effects linear regression *estimate (97.5%CI)*	*p*
Bayley cognitive III/IV score			-0.50 (-3.65;2.63)	0.721
median [IQR]	95.00 [80.00;105.00]	95.00 [83.75;100.00]		
mean (97.5%CI)	93.36 (91.04;95.68)	92.46 (89.96;94.96)		
missing	447 (61.5%)	429 (64.5%)		

Not filtered for death

	Control group *n = 741*	Experimental group *n = 697*	Logistic regression *RR (95%CI)*	*p*
**Components of the dichotomous outcome**				
Cerebral palsy	22 (3.0%)	25 (3.6%)	1.30 (0.79;2.14)	0.310
missing	246 (33.2%)	244 (35.0%)		
Visual impairment	43 (5.8%)	25 (3.6%)	0.56 (0.38;0.80)	0.002
missing	248 (33.5%)	244 (35.0%)		
Hearing impairment	14 (1.9%)	16 (2.3%)	1.15 (0.69;1.92)	0.582
missing	244 (32.9%)	246 (35.3%)		
Cognitive impairment	116 (15.7%)	106 (15.2%)	0.97 (0.77;1.22)	0.814
missing	256 (34.5%)	253 (36.3%)		
**Exploratory outcomes**				
Any other chronic illness	154 (20.8%)	117 (16.8%)	0.80 (0.65;0.98)	0.032
missing	248 (33.5%)	246 (35.3%)		
Any daily medication for the last two months	108 (14.6%)	93 (13.3%)	0.91 (0.71;1.17)	0.477
missing	253 (34.1%)	246 (35.3%)		
Hospitalisation since discharge from index hospitalisation	186 (25.1%)	181 (26.0%)	1.06 (0.91;1.23)	0.446
missing	360 (48.6%)	348 (49.9%)		
Parental report of thriving child	368 (49.7%)	337 (48.4%)	1.00 (0.97;1.03)	0.955
missing	360 (48.6%)	349 (50.1%)		
Parental report of worries regarding the child	144 (19.4%)	124 (17.8%)	0.96 (0.79;1.17)	0.688
missing	367 (49.5%)	356 (51.1%)		

Without informal assessment

	Control group *n = 741*	Experimental group *n = 697*	Logistic regression *RR (97.5%CI)*	*p*
Death or moderate-or-severe NDD	321 (43.3%)	292 (41.9%)	0.96 (0.85;1.07)	0.449
missing	72 (9.7%)	77 (11.0%)		

Best-Worst Worst-Best - Death or moderate-or-severe NDD

	**Control group** *n = 741*	**Experimental group** *n = 697*	**Logistic regression** *RR (97.5%CI)*	*p*
Death or moderate-or-severe NDD	393 (53.0%)	292 (41.9%)	0.78 (0.70;0.87)	0.000

	**Control group** *n = 741*	**Experimental group** *n = 697*	**Logistic regression** *RR (97.5%CI)*	*p*
Death or moderate-or-severe NDD	321 (43.3%)	369 (52.9%)	1.21 (1.08;1.35)	0.001

Multiple imputations - Death or moderate-or-severe NDD

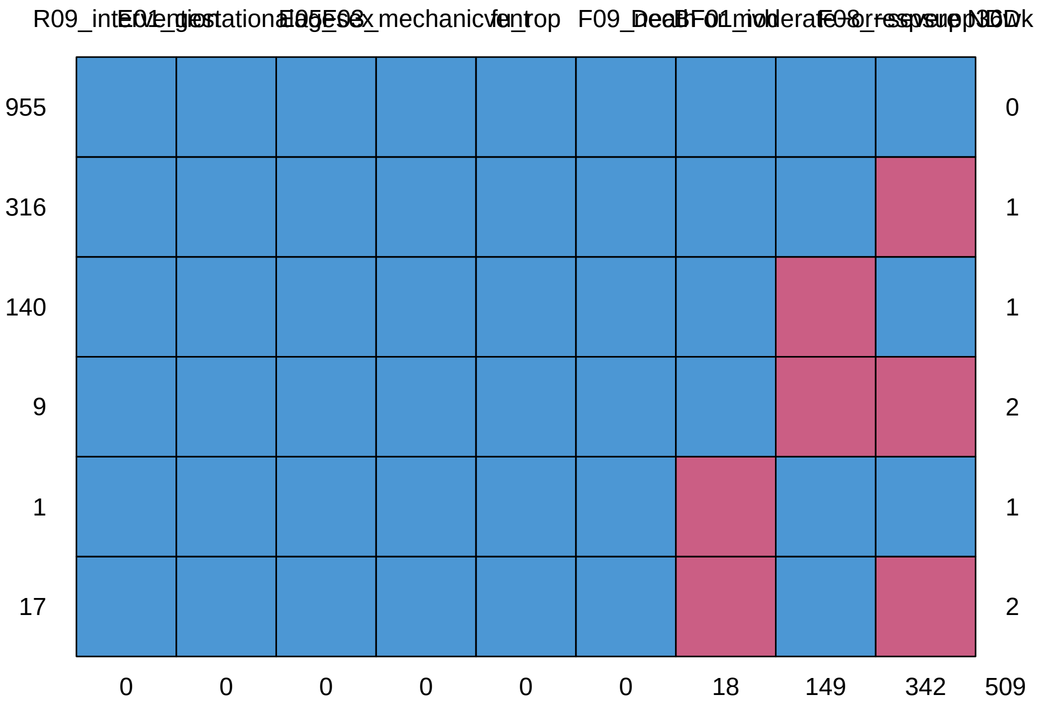


```
Complete data summary


  No  Yes NA's
 676  613  149


Imputed data summary
```

```
 No Yes
756 682
```

	**Control group** *n = 741*	**Experimental group** *n = 697*	**Logistic regression** *RR (97.5%CI)*	*p*
Death or moderate-or-severe NDD	352 (47.5%)	330 (47.3%)	0.98 (0.88;1.10)	0.769

High and low follow-up rates

High follow-up rate

	Control group *n = 441*	Experimental group *n = 396*	Logistic regression *RR (97.5%CI)*	*p*
Death or moderate-or-severe NDD	206 (46.7%)	182 (46.0%)	0.96 (0.83;1.11)	0.595
missing	18 (4.1%)	15 (3.8%)		

	Control group *n = 165*	Experimental group *n = 155*	Mixed effects linear regression *estimate (97.5%CI)*	*p*
Bayley cognitive III/IV score			-1.75 (-6.19;2.61)	0.371
median [IQR]	95.00 [85.00;105.00]	95 [80.00;100.00]		
mean (97.5%CI)	94.33 (91.27;97.40)	91.63 (88.02;95.25)		
missing	45 (27.3%)	54 (34.8%)		

Low follow-up rate

	Control group *n = 300*	Experimental group *n = 301*	Logistic regression *RR (97.5%CI)*	*p*
Death or moderate-or-severe NDD	115 (38.3%)	110 (36.5%)	0.95 (0.76;1.19)	0.603
missing	54 (18.0%)	62 (20.6%)		

	Control group *n = 576*	Experimental group *n = 542*	Mixed effects linear regression *estimate (97.5%CI)*	*p*
Bayley cognitive III/IV score			1.06 (-3.25;5.36)	0.582
median [IQR]	95.00 [80.00;105.00]	95 [85.00;105.00]		
mean (97.5%CI)	92.33 (88.99;95.67)	93.66 (90.33;96.99)		
missing	412 (71.5%)	397 (73.2%)		

Random-effects meta-analysis

Death or moderate-or-severe NDD

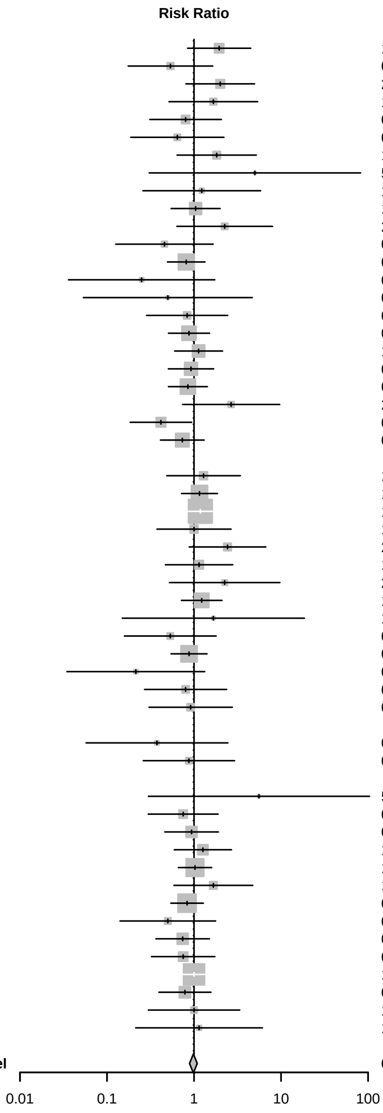

Study	RR	95%−CI
22	1.94	[0.84; 4.48]
60	0.54	[0.17; 1.64]
39	2.00	[0.81; 4.96]
86	1.67	[0.52; 5.39]
62	0.80	[0.31; 2.06]
97	0.64	[0.19; 2.20]
96	1.82	[0.64; 5.20]
10	5.00	[0.30; 81.97]
52	1.23	[0.26; 5.82]
54	1.04	[0.55; 1.99]
50	2.25	[0.63; 7.97]
59	0.46	[0.13; 1.66]
76	0.81	[0.49; 1.34]
29	0.25	[0.04; 1.73]
45	0.50	[0.05; 4.67]
65	0.83	[0.28; 2.44]
81	0.88	[0.51; 1.51]
83	1.13	[0.60; 2.13]
68	0.92	[0.51; 1.68]
92	0.85	[0.51; 1.42]
48	2.67	[0.74; 9.65]
44	0.42	[0.18; 0.94]
82	0.73	[0.41; 1.31]
16		
80	1.29	[0.49; 3.40]
27	1.16	[0.72; 1.86]
98	1.18	[0.85; 1.64]
69	1.00	[0.38; 2.66]
41	2.42	[0.88; 6.68]
61	1.14	[0.47; 2.79]
87	2.25	[0.52; 9.70]
90	1.22	[0.72; 2.10]
20	1.67	[0.15; 18.54]
78	0.53	[0.16; 1.79]
40	0.88	[0.54; 1.41]
70	0.21	[0.03; 1.33]
46	0.80	[0.27; 2.37]
37	0.91	[0.30; 2.75]
23		
26	0.38	[0.06; 2.45]
21	0.87	[0.26; 2.92]
24		
25	5.57	[0.30; 103.81]
28	0.75	[0.30; 1.89]
35	0.94	[0.46; 1.90]
43	1.26	[0.59; 2.70]
64	1.03	[0.66; 1.59]
85	1.67	[0.59; 4.73]
31	0.83	[0.54; 1.28]
66	0.50	[0.14; 1.77]
47	0.74	[0.36; 1.50]
38	0.75	[0.32; 1.74]
79	1.00	[0.69; 1.45]
71	0.79	[0.39; 1.56]
15	1.00	[0.30; 3.35]
77	1.14	[0.21; 6.11]
**Random effects model**	**0.98**	**[0.89; 1.09]**

```
Number of studies: k = 53
Number of observations: o = 1289 (o.e = 620, o.c = 669)
Number of events: e = 613

                         RR           95%-CI     z p-value
Random effects model 0.9827 [0.8870; 1.0888] -0.33  0.7390

Quantifying heterogeneity (with 95%-CIs):
 tau^2 = 0 [0.0000; 0.0635]; tau = 0 [0.0000; 0.2519]
 I^2 = 0.0% [0.0%; 32.2%]; H = 1.00 [1.00; 1.21]

Test of heterogeneity:
     Q d.f. p-value
 42.95   52  0.8102

Details of meta-analysis methods:
- Inverse variance method
- Restricted maximum-likelihood estimator for tau^2
- Q-Profile method for confidence interval of tau^2 and tau
- Calculation of I^2 based on Q
- Continuity correction of 0.5 in studies with zero cell frequencies
```

Bayley cognitive III/IV score

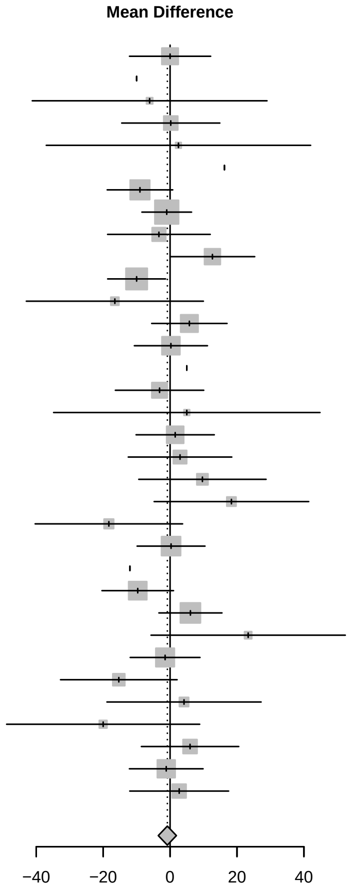

Study	MD	95%−CI
10	0.00	[−12.13; 12.13]
20	−10.00	
22	−6.11	[−41.20; 28.97]
27	0.21	[−14.46; 14.87]
31	2.50	[−37.00; 42.00]
35	16.25	
39	−9.00	[−18.80; 0.80]
40	−1.00	[−8.43; 6.43]
41	−3.33	[−18.67; 12.01]
44	12.66	[0.02; 25.30]
48	−10.00	[−18.65; −1.35]
50	−16.50	[−42.96; 9.96]
52	5.76	[−5.50; 17.03]
54	0.25	[−10.68; 11.18]
59	5.00	
60	−3.15	[−16.36; 10.06]
61	5.00	[−34.81; 44.81]
62	1.53	[−10.16; 13.22]
64	2.98	[−12.50; 18.45]
68	9.67	[−9.36; 28.70]
69	18.33	[−4.77; 41.43]
71	−18.29	[−40.32; 3.74]
76	0.29	[−9.86; 10.44]
79	−12.00	
80	−9.67	[−20.36; 1.03]
81	6.08	[−3.35; 15.51]
82	23.33	[−5.70; 52.37]
83	−1.49	[−11.92; 8.95]
86	−15.32	[−32.75; 2.11]
87	4.17	[−18.87; 27.21]
90	−20.00	[−48.85; 8.85]
92	5.97	[−8.58; 20.53]
96	−1.15	[−12.16; 9.87]
97	2.72	[−12.06; 17.50]
**Random effects model**	**−0.82**	**[−3.53; 1.89]**

```
Number of studies: k = 30
Number of observations: o = 525 (o.e = 243, o.c = 282)

                          MD            95%-CI     z p-value
Random effects model -0.8160 [-3.5266; 1.8946] -0.59  0.5552

Quantifying heterogeneity (with 95%-CIs):
 tau^2 = 8.1419 [0.0000; 66.6408]; tau = 2.8534 [0.0000; 8.1634]
 I^2 = 15.0% [0.0%; 45.7%]; H = 1.08 [1.00; 1.36]

Test of heterogeneity:
     Q d.f. p-value
 34.12   29  0.2347

Details of meta-analysis methods:
- Inverse variance method
- Restricted maximum-likelihood estimator for tau^2
- Q-Profile method for confidence interval of tau^2 and tau
- Calculation of I^2 based on Q
```

Generalised estimating equation

Death or moderate-or-severe NDD

```
Relative risk: 0.8792214

 97.5% Confidence interval: 0.7092358 ; 1.089948

 P-value: 0.1792
```

Predictors	Odds Ratios	CI	p
(Intercept)	0.06	0.00 – 1710.79	0.541
arm [Experimental group]	0.70	0.40 – 1.23	0.160
twins	1.00	0.99 – 1.02	0.427
site id [BE01]	11.41	0.00 – 49109.83	0.514
site id [BE02]	39.00	0.01 – 145717.12	0.318
site id [BE03]	11.07	0.00 – 35250.07	0.504
site id [BE06]	14.48	0.01 – 36360.28	0.444
site id [CH01]	1.03	0.02 – 49.02	0.987
site id [CH03]	1.46	0.04 – 49.77	0.809
site id [CH04]	0.67	0.01 – 32.07	0.815
site id [CH05]	0.79	0.03 – 21.26	0.875
site id [CZ01]	0.20	0.01 – 4.37	0.240
site id [CZ02]	0.34	0.01 – 13.40	0.512
site id [DE01]	32.14	0.00 – 880740.51	0.447
site id [DK01]	18.24	0.00 – 245984.42	0.494
site id [DK04]	12.78	0.00 – 99498.61	0.524
site id [DK18]	2.64	0.00 – 16926.08	0.804
site id [DK30]	20.12	0.00 – 114942.19	0.437
site id [ES01]	9.02	0.01 – 12849.54	0.497
site id [ES02]	6.45	0.01 – 4676.57	0.526
site id [ES03]	5.80	0.01 – 2284.66	0.510
site id [ES05]	13.32	0.05 – 3504.41	0.298
site id [ES06]	5.67	0.03 – 1248.19	0.471
site id [ES08]	5.44	0.04 – 810.99	0.448
site id [ES09]	12.81	0.11 – 1523.01	0.232
site id [ES12]	0.00	0.00 – 0.00	<0.001
site id [GR01]	3.84	0.04 – 381.61	0.512
site id [GR02]	10.11	0.14 – 727.64	0.225
site id [GR03]	14.54	0.14 – 1527.88	0.197
site id [GR04]	8.94	0.14 – 587.51	0.241
site id [IE01]	2.16	0.10 – 44.65	0.569
site id [IE02]	2.12	0.12 – 38.61	0.563
site id [IE03]	0.80	0.05 – 12.86	0.860
site id [IE04]	9.52	0.57 – 158.52	0.072
site id [IN01]	1.34	0.08 – 21.47	0.811
site id [IT01]	1.77	0.10 – 30.20	0.651
site id [IT07]	3.42	0.42 – 27.65	0.188
site id [IT09]	1.72	0.19 – 15.35	0.578
site id [NY10]	0.43	0.07 – 2.66	0.296
site id [PL01]	0.61	0.12 – 3.20	0.504
site id [PL02]	0.00	0.00 – 0.00	<0.001
site id [PL03]	1.56	0.19 – 12.98	0.641
site id [PL04]	0.38	0.08 – 1.75	0.157
site id [PL06]	0.97	0.07 – 13.78	0.977
site id [PL07]	0.11	0.01 – 1.92	0.083
site id [PL08]	1.75	0.38 – 8.14	0.412

Predictors	Odds Ratios	CI	p
site id [TR01]	1.72	0.36 – 8.23	0.435
site id [TR02]	0.65	0.16 – 2.73	0.504
site id [TR03]	1.61	0.36 – 7.28	0.481
site id [TR04]	1.28	0.18 – 9.05	0.776
site id [TR05]	1.32	0.21 – 8.34	0.735
site id [TR06]	0.48	0.05 – 4.68	0.472
site id [UK08]	0.78	0.08 – 7.72	0.809
site id [UK09]	1.10	0.09 – 13.59	0.933
site id [US02]	1.91	0.13 – 27.15	0.584
site id [US03]	0.52	0.04 – 7.09	0.576
site id [US04]	58274631980531360.00	3563537514361635.00 – 952966741273301504.00	<0.001
site id [US05]	0.12	0.01 – 2.19	0.101
R07 galessthan26wks [2]	0.28	0.21 – 0.37	<0.001
arm [Experimental group]× twins	1.00	1.00 – 1.00	0.281
N ssid	1289	1289	1289
Observations	1289		

Bayley cognitive III/IV score

```
Mean difference: 1.689709

 97.5% Confidence interval: -4.330156 ; 7.709575

 P-value: 0.5291
```

	estimate	san.se	wald	p
(Intercept)	81.0791706	42.0904334	3.7106654	0.0540658
armExperimental group	1.6897094	2.6850426	0.3960241	0.5291497
twins	0.0349896	0.1100420	0.1011025	0.7505106
site_idBE01	3.0343588	35.4847641	0.0073122	0.9318547
site_idBE02	-2.4719873	33.4258662	0.0054692	0.9410467
site_idBE03	2.9894448	32.4563213	0.0084836	0.9266133
site_idBE06	3.1827814	30.8367167	0.0106531	0.9177931
site_idCH01	7.5682764	14.9583927	0.2559907	0.6128881
site_idCH03	-0.1524382	12.4947379	0.0001488	0.9902659
site_idCH04	5.4598175	11.3717792	0.2305151	0.6311421
site_idCH05	5.7251139	10.3163547	0.3079752	0.5789254
site_idCZ01	-2.2811403	10.1806165	0.0502060	0.8227052
site_idCZ02	-10.3372682	13.7667397	0.5638315	0.4527206
site_idDE01	-3.7679651	41.6732436	0.0081752	0.9279559
site_idDK01	14.5647646	38.4774456	0.1432829	0.7050389
site_idDK04	17.0663795	36.3309395	0.2206630	0.6385352
site_idES01	9.5137903	28.9472378	0.1080172	0.7424129
site_idES02	-4.0873468	26.5107610	0.0237705	0.8774703
site_idES03	-0.3949923	24.2928322	0.0002644	0.9870273
site_idES05	-9.8850437	22.6980440	0.1896620	0.6631981
site_idES06	3.9125496	20.9370156	0.0349213	0.8517607
site_idES08	-1.9321439	19.4193742	0.0098994	0.9207447
site_idES09	2.9525435	19.6530032	0.0225702	0.8805802
site_idGR01	4.9099678	17.1962782	0.0815245	0.7752419
site_idGR02	-12.2590695	16.9282485	0.5244342	0.4689563
site_idGR04	-16.6011322	16.4568640	1.0176097	0.3130867
site_idIE01	6.7543962	9.4802359	0.5076154	0.4761730
site_idIE02	18.3292417	9.3038807	3.8811527	0.0488310
site_idIE03	-1.1670857	8.7111514	0.0179496	0.8934215
site_idIE04	-13.2443839	10.4115374	1.6182060	0.2033421
site_idIN01	-9.4361009	7.7800944	1.4710093	0.2251865
site_idIT07	-14.3529407	6.6687375	4.6322772	0.0313759
site_idIT09	-29.0953096	6.1958927	22.0515028	0.0000027
site_idTR01	-1.1073230	9.7736199	0.0128362	0.9097950
site_idTR03	-4.5046568	7.6151222	0.3499207	0.5541580
site_idTR05	-20.1601541	9.2054147	4.7962408	0.0285219
site_idUK08	-2.6026293	7.5155710	0.1199225	0.7291186
site_idUS02	-15.7225779	10.2198236	2.3667954	0.1239412
site_idUS03	-18.3427757	9.2426005	3.9385988	0.0471901
site_idUS04	-46.4159326	8.3584005	30.8381141	0.0000000
R07_galessthan26wks2	6.5245619	1.4553690	20.0981678	0.0000074
armExperimental group:twins	-0.0067321	0.0103694	0.4214885	0.5161953

Skewness of missingness

	Control group<br>*n = 741*	Experimental group<br>*n = 697*	Fisher's exact test<br>*OR (95%CI)*	*p*
Death or moderate-or-severe NDD is missing	72 (9.7%)	77 (11.0%)	1.15 (0.81;1.65)	0.436
Bayley cognitive III/IV score is missing	457 (61.7%)	451 (64.7%)	1.14 (0.91;1.42)	0.251

All PARCAR scores and non-parametric for PARCAR scores

	Control group<br>*n = 741*	Experimental group<br>*n = 697*	Mixed effects linear regression<br>*estimate (95%CI)*	*p*
PARCAR score (extrapolated)			-0.88 (-3.99;2.23)	0.581
median [IQR]	89.00 [76.00;101.00]	88.00 [76.00;100.00]		
mean (95%CI)	87.75 (85.38;90.12)	86.49 (84.15;88.83)		
missing	404 (54.5%)	377 (54.1%)		

	Wilcoxon rank sum test<br>*median diff. (95%HLCI)*	*p*
PARCAR score	0.00 (-4.00;3.00)	0.830
median [IQR]		
mean (95%CI)		
missing		
PARCAR score (extrapolated)	-1.00 (-4.00;2.00)	0.575
median [IQR]		
mean (95%CI)		
missing		

`PARCAR score - VanElteren`

	Estimate	Lower	Upper	Pvalue
outcome	0.4982523	0.4512536	0.545251	0.9418992

`PARCAR score (extrapolated) - VanElteren`

	Estimate	Lower	Upper	Pvalue
outcome	0.4938438	0.4520063	0.5356812	0.7730386

Added after statistical report

Histograms of continuous outcomes

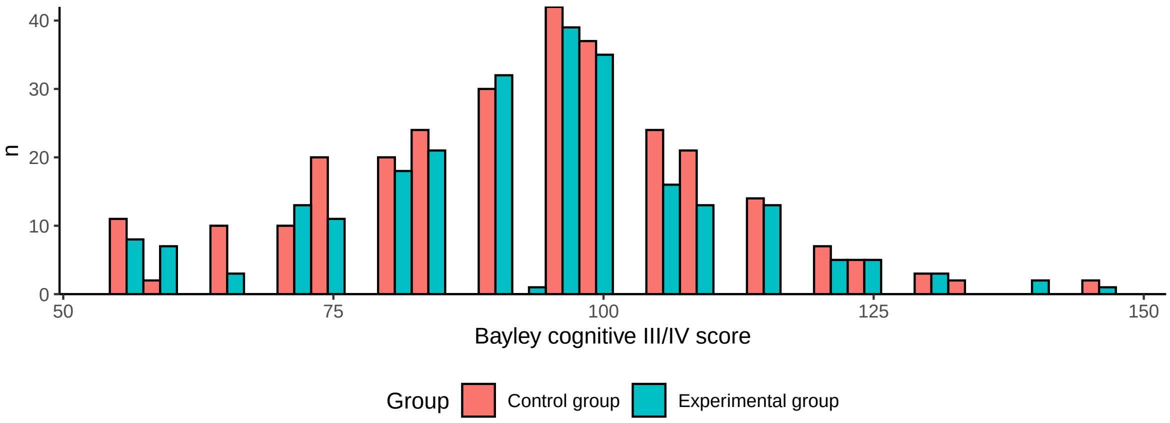

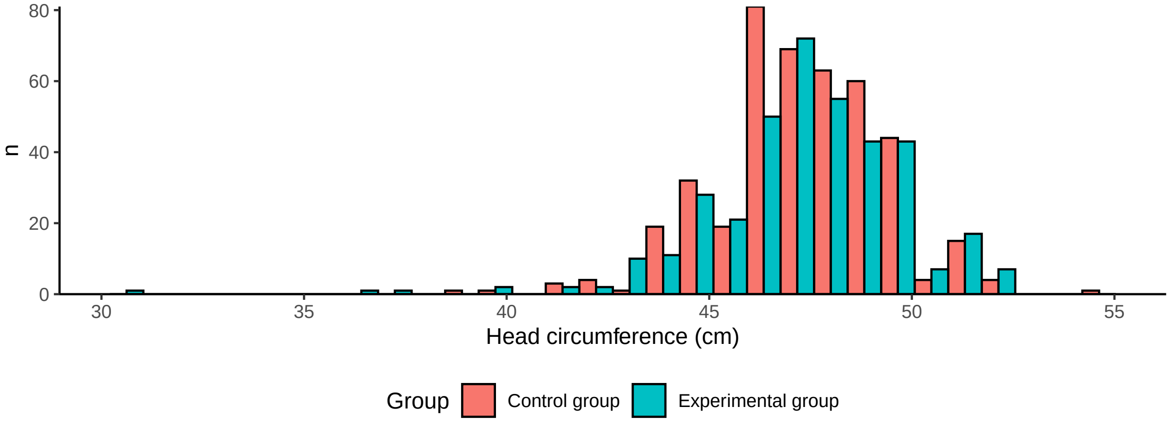

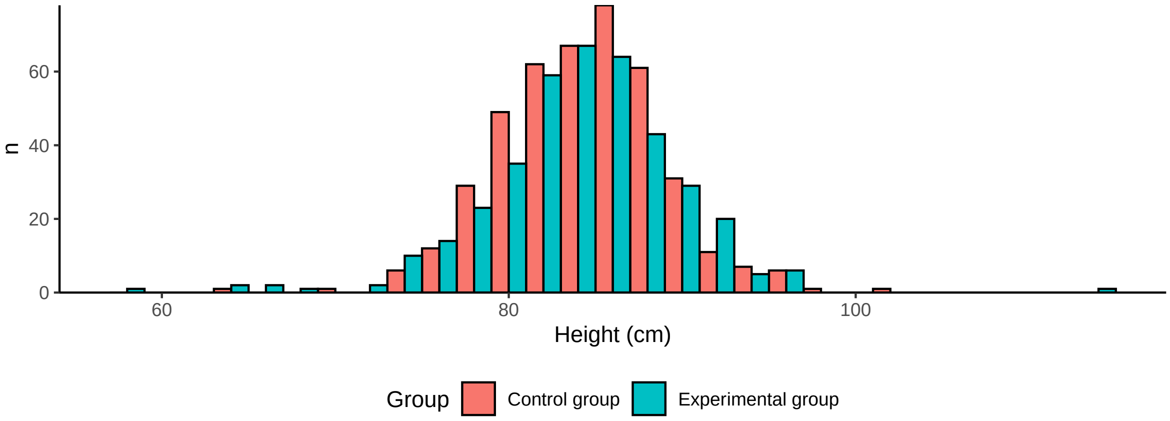
n
0
20
40
60
60
80
100
Height (cm)
Group
Control group
Experimental group

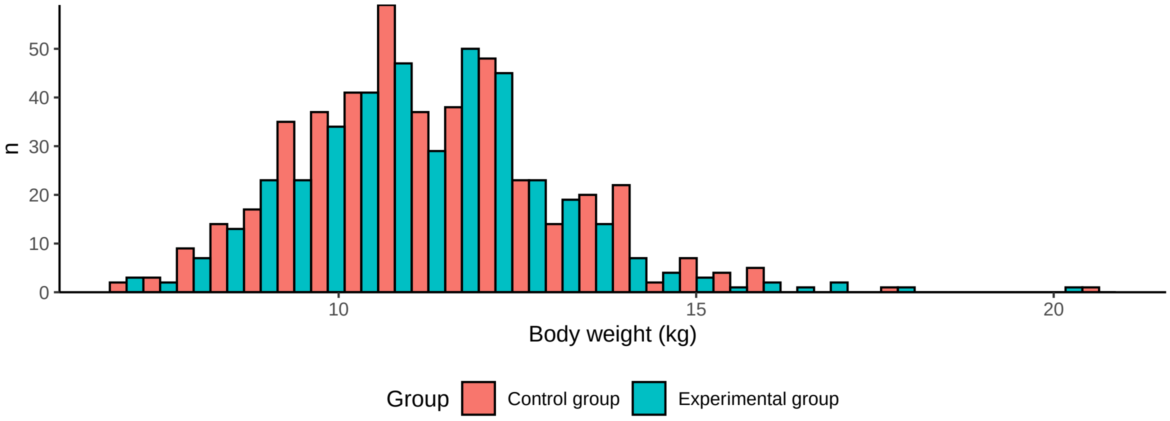
n
0
10
20
30
40
50
10
15
20
Body weight (kg)
Group
Control group
Experimental group

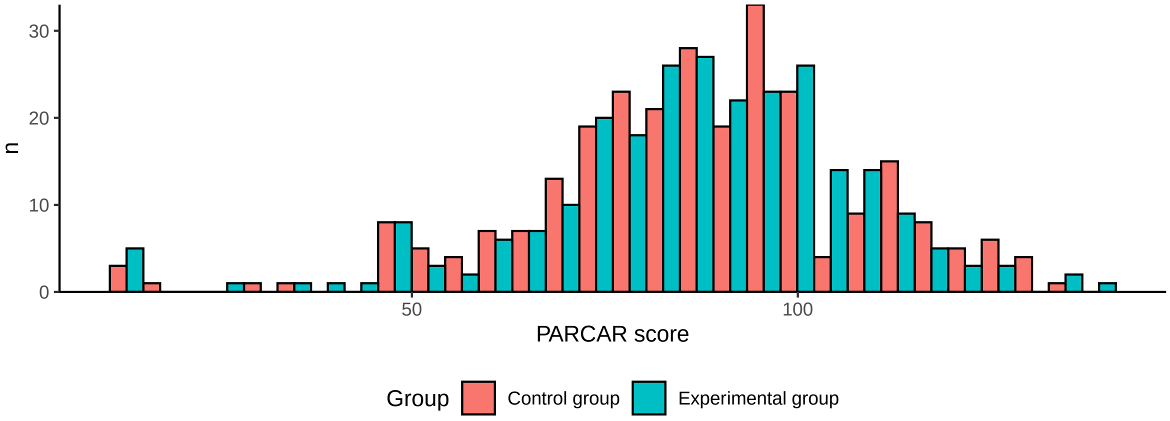
n
0
10
20
30
50
100
PARCAR score
Group
Control group
Experimental group

Per-protocol

	Control group<br>*n = 741*	Experimental group<br>*n = 697*
perprotocol	727 (98.1%)	665 (95.4%)

ROP

ROP er enten ROP v. 36 wks eller ROP v. 2 y

	Control group<br>*n = 741*	Experimental group<br>*n = 697*
Retinopathy of prematurity	88 (11.9%)	97 (13.9%)
ROP_36wk	61 (8.2%)	66 (9.5%)
ROP_2y	48 (6.5%)	54 (7.7%)
missing	291 (39.3%)	293 (42.0%)

SD for continuous outcomes

	Control group<br>*n = 741*	Experimental group<br>*n = 697*
Bayley cognitive III/IV score - mean (SD)	93.18 (17.27)	92.83 (17.00)
missing	457 (61.7%)	451 (64.7%)
Head circumference (cm) - mean (SD)	47.31 (2.08)	47.32 (2.43)
missing	320 (43.2%)	324 (46.5%)
Height (cm) - mean (SD)	84.90 (4.64)	84.57 (5.54)
missing	318 (42.9%)	313 (44.9%)
Body weight (kg) - mean (SD)	11.28 (1.82)	11.17 (1.74)
missing	302 (40.8%)	302 (43.3%)
PARCAR score - mean (SD)	87.42 (21.26)	86.44 (21.18)
missing	473 (63.8%)	439 (63%)
PARCAR score (extrapolated) - mean (SD)	87.75 (22.12)	86.49 (21.26)
missing	404 (54.5%)	377 (54.1%)

Additional follow-up times

	Control group<br>*n = 741*	Experimental group<br>*n = 697*
Weight follow-up (corrected, months)		
median [IQR]	24.33 [23.13;25.45]	24.35 [22.40;25.55]
mean (95%CI)	24.05 (23.77;24.34)	23.80 (23.44;24.15)
missing	296 (39.9%)	295 (42.3%)
Height follow-up (corrected, months)		
median [IQR]	24.33 [23.15;25.44]	24.31 [22.09;25.53]
mean (95%CI)	23.88 (23.56;24.21)	23.53 (23.14;23.93)
missing	306 (41.3%)	305 (43.8%)
Head circumference follow-up (corrected, months)		
median [IQR]	24.17 [21.80;25.32]	24.15 [21.29;25.30]
mean (95%CI)	23.25 (22.86;23.64)	23.09 (22.66;23.52)

	Control group *n = 741*	Experimental group *n = 697*
missing	310 (41.8%)	315 (45.2%)

Treatment specific variables

	Control group *n = 741*	Experimental group *n = 697*
**Cerebral oximetry**		
Age at initiation of cerebral oximetry monitoring (hours)		
median [IQR]	NA	3 [2.00;4.00]
mean (95%CI)	NA	3.19 (2.88;3.49)
missing	741 (100%)	0 (0%)
Cerebral oximetry monitoring discontinued >14 hours	0 (0.0%)	32 (4.6%)
missing	741 (100.0%)	0 (0.0%)
Change of medical management due to cerebral hypoxia	0 (0.0%)	199 (28.6%)
missing	741 (100.0%)	0 (0.0%)
Cerebral oximetry monitoring in control group	45 (6.1%)	0 (0.0%)
missing	0 (0.0%)	695 (99.7%)

ICC

Death or moderate-or-severe NDD

```
Triplets: 15
Twins: 302
Singleton: 972
```

Table 38: ICC estimates

Methods	ICC
ANOVA Estimate	0.413961664912899
Modified ANOVA Estimate	0.411311157327777
Moment Estimate with Equal Weights	-
Moment Estimate with Weights Proportional to Cluster Size	-
Modified Moment Estimate with Equal Weights	-
Modified Moment Estimate with Weights Proportional to Cluster Size	-
Stabilized Moment Estimate	0.856310788067325
Moment Estimate from Unbiased Estimating Equation	0.413974852656603
Fleiss-Cuzick Kappa Type Estimate	0.411299420204042
Mak's Unweighted Average Estimate	0.403698716715671
Correlation Estimate with Equal Weight to Every Pair of Observations	0.420930232558139
Correlation Estimate with Equal Weight to Each Cluster Irrespective of Size	0.401009288378556
Correlation Estimate with Weighting Each Pair According to Number of Pairs individuals Appear	0.406225091662681
Resampling Estimate	0.425564558949941
First-order Model Linearized Estimate	0.399651294609857
Monte Carlo Simulation Estimate	0.310325863254754

Table 39: ICC confidence intervals

Type	LowerCI	UpperCI
Smith's Large Sample Confidence Interval	0.2854902	0.5424331
Zou and Donner's Modified Wald Confidence Interval	0.0000000	1.0000000
Fleiss-Cuzick Confidence Interval	0.2702005	0.5523983
Pearson Correlation Type Confidence Interval	0.2792075	0.5626529
Resampling Based Confidence Interval	0.2602062	0.5909230

Bayley cognitive III/IV score

```
Triplets: 9
Twins: 96
Singleton: 425
```

```
ICC for kontinuert outcome: 0.563
```

Table 40: Variance Components and ICC Estimate

Component	Estimate
Group Variance	112.7630599
Residual Variance	87.5013603
ICC	0.5630709

PARCAR additional analyses

Primary outcome with extrapolated data

	Control group *n = 741*	Experimental group *n = 697*	Logistic regression *RR (97.5%CI)*	*p*
Death or moderate-or-severe NDD e	323 (43.6%)	294 (42.2%)	0.95 (0.85;1.07)	0.409
missing	72 (9.7%)	74 (10.6%)		

Grouping

	Control group *n = 741*	Experimental group *n = 697*
PARCAR is		
extrapolated	69 (9.3%)	62 (8.9%)
standard score	268 (36.2%)	258 (37.0%)
missing	404 (54.5%)	377 (54.1%)

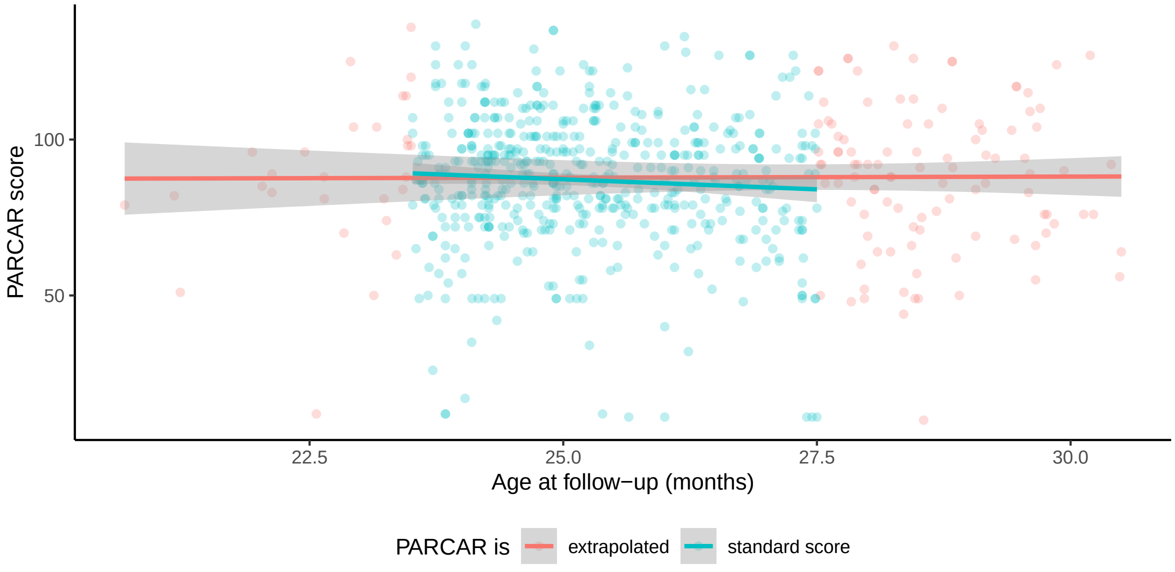

Who saw (only alive)

Table 43: Stratified by group

	Control group *n = 576*	Experimental group *n = 535*
specialist_combination		
Neuropediatrician, Pediatrician/neonatologist, Physiotherapist, Psychologist	24 (4.2%)	25 (4.7%)
Neuropediatrician, Pediatrician/neonatologist, Physiotherapist	13 (2.3%)	11 (2.1%)
Neuropediatrician, Pediatrician/neonatologist, Psychologist	24 (4.2%)	17 (3.2%)
Pediatrician/neonatologist, Physiotherapist, Psychologist	28 (4.9%)	21 (3.9%)
Neuropediatrician, Physiotherapist, Psychologist	4 (0.7%)	4 (0.7%)
Neuropediatrician, Pediatrician/neonatologist	15 (2.6%)	12 (2.2%)
Pediatrician/neonatologist, Physiotherapist	29 (5.0%)	29 (5.4%)
Pediatrician/neonatologist, Psychologist	87 (15.1%)	66 (12.3%)
Neuropediatrician, Physiotherapist	2 (0.3%)	2 (0.4%)
Neuropediatrician, Psychologist	17 (3.0%)	18 (3.4%)
Pediatrician/neonatologist	170 (29.5%)	160 (29.9%)
Neuropediatrician	29 (5.0%)	27 (5.0%)
Physiotherapist	2 (0.3%)	1 (0.2%)
Psychologist	8 (1.4%)	14 (2.6%)
None	124 (21.5%)	128 (23.9%)

Table 44: Stratified by Death or moderate-to-severe NDD

	No *n = 676*	Yes *n = 286*
specialist_combination		
Neuropediatrician, Pediatrician/neonatologist, Physiotherapist, Psychologist	24 (3.6%)	25 (8.7%)
Neuropediatrician, Pediatrician/neonatologist, Physiotherapist	11 (1.6%)	13 (4.5%)
Neuropediatrician, Pediatrician/neonatologist, Psychologist	27 (4.0%)	14 (4.9%)
Pediatrician/neonatologist, Physiotherapist, Psychologist	31 (4.6%)	18 (6.3%)
Neuropediatrician, Physiotherapist, Psychologist	5 (0.7%)	3 (1.0%)
Neuropediatrician, Pediatrician/neonatologist	15 (2.2%)	12 (4.2%)
Pediatrician/neonatologist, Physiotherapist	43 (6.4%)	15 (5.2%)
Pediatrician/neonatologist, Psychologist	108 (16.0%)	45 (15.7%)
Neuropediatrician, Physiotherapist	2 (0.3%)	2 (0.7%)
Neuropediatrician, Psychologist	23 (3.4%)	12 (4.2%)
Pediatrician/neonatologist	254 (37.6%)	76 (26.6%)
Neuropediatrician	30 (4.4%)	26 (9.1%)
Physiotherapist	2 (0.3%)	1 (0.3%)
Psychologist	16 (2.4%)	6 (2.1%)
None	85 (12.6%)	18 (6.3%)

Death or moderate-to-severe NDD - Tier 3 outcome

	Control group *n = 73*	Experimental group *n = 60*	Logistic regression *RR (97.5%CI)*	*p*
Death or moderate-or-severe NDD	14 (19.2%)	5 (8.3%)	0.58 (0.25;1.37)	0.218

Cognitive impairment - Where Cognitive impairment other than bailey is responsible for change primary outcome from no to yes

	Control group *n = 25*	Experimental group *n = 20*
F15a_nameoftest		
ASQ	1 (4.0%)	1 (5.0%)
ASQ-3	1 (4.0%)	0 (0.0%)
ASQ 24 m Q	0 (0.0%)	2 (10.0%)
ASQ 24 mdr	1 (4.0%)	1 (5.0%)
asq24	3 (12.0%)	6 (30.0%)
ASQ24	1 (4.0%)	0 (0.0%)
ASQ3	1 (4.0%)	1 (5.0%)
Battelle developmental inventary: cognitive	1 (4.0%)	0 (0.0%)
Battelle developmental inventory screening: cognitive	1 (4.0%)	0 (0.0%)
Bayley	1 (4.0%)	0 (0.0%)
Brunet-Lezzine-R	0 (0.0%)	1 (5.0%)
Denver II	0 (0.0%)	1 (5.0%)
Developmental profile 3	1 (4.0%)	0 (0.0%)
Developmental Profile 3	0 (0.0%)	1 (5.0%)
DP3	1 (4.0%)	0 (0.0%)
griffiths-3	1 (4.0%)	0 (0.0%)
Griffiths-3	1 (4.0%)	0 (0.0%)
Griffiths-III	2 (8.0%)	0 (0.0%)
Griffiths III	6 (24.0%)	4 (20.0%)
Griffiths III, score corrected for prematurity	0 (0.0%)	1 (5.0%)
Haizea-Llevant	1 (4.0%)	0 (0.0%)
Schedule of Growing Skills Assessment	1 (4.0%)	0 (0.0%)
Schedule of Growing skills assment	0 (0.0%)	1 (5.0%)

Lost to follow-up

	Control group *n = 741*	Experimental group *n = 697*
Lost to follow-up	65 (8.8%)	70 (10.0%)
missing	7 (0.9%)	7 (1.0%)

	Control group *n = 65*	Experimental group *n = 70*
F00a_lostfollowup_reason		
Clinical follow-up in hospital with no access to data	1 (1.5%)	6 (8.6%)
No consent to use data	21 (32.3%)	17 (24.3%)
Other	17 (26.2%)	17 (24.3%)
The family moved away	14 (21.5%)	17 (24.3%)
Unknown	12 (18.5%)	13 (18.6%)
