# Supplementary material for: Cerebral Oximetry in Extremely Preterm Infants: 2-Year Follow-Up of the SafeBoosC-III Randomized Clinical Trial
Source: JAMA Pediatr. 2026 Apr 20;180(6):619–27. doi: 10.1001/jamapediatrics.2026.1066 (PMC13097032; doi:10.1001/jamapediatrics.2026.1066)
Supplement: Supplement 3. — eAppendix. List of Investigators eMethods. eTable 1. Death or Severe Brain Injury at 36 Weeks’ Postmenstrual Age and at 2-Year Follow-Up in Included and Excluded Sites eTable 2. Causes of Death Beyond 36 Weeks’ Postmenstrual Age eTable 3. Per-Protocol Analysis eTable 4. Multiple Imputation Analysis on Death or Moderate or Severe Neurodevelopmental Disability eTable 5. Best-Worst and Worst-Best Case Analysis on Death or Moderate or Severe Neurodevelopmental Disability eTable 6. Exclusion of Informal Assessment (Tier 3) for the Coprimary Outcome Death or Moderate or Severe Neurodevelopmental Disability eTable 7. Number of Events in Coprimary Outcome Death or Moderate or Severe Neurodevelopmental Disability for Informal Assessments (Tier 3) eTable 8. Comparison of Sites With High Follow-Up Rates (≥90%) vs Low Follow-Up Rates (<90%) for the Coprimary Outcome Death or Moderate or Severe Neurodevelopmental Disability eTable 9. Comparison of Sites With High Follow-Up Rates (≥90 vs Low Follow-Up Rates (<90%) for the Coprimary Outcome Bayley III/IV Cognitive Score eTable 10. Generalized Estimation Equation (Twin Sensitivity Analysis) eTable 11. Proportion of Missingness Between Cerebral Oximetry Group and Experimental Group for the 2 Coprimary Outcomes eTable 12. Analysis of all PARCA-R Nonverbal Cognitive Scores Including Extrapolated Scores eTable 13. Death or Moderate or Severe Neurodevelopmental Disability With PARCA-R Nonverbal Cognitive Extrapolated Scores eTable 14. Follow-Up Times of Randomized Infants eTable 15. Clinical Data Based on Information From Health Care Professional eTable 16. Parental Education Level Based on ISCED Classification eTable 17. Number of Randomizations per Site eFigure 1. Prioritization of Data for the Coprimary Outcome Moderate or Severe Neurodevelopmental Disability eFigure 2. Random Effect Meta-Analysis for Coprimary Outcome Moderate or Severe Neurodevelopmental Disability and Bayley III/IV Cognitive Score eFigure 3. Extract From the Elect [file jamapediatr-e261066-s003.pdf]

Supplementary Online Content

Rasmussen MIS, Hansen ML, Pellicer A, et al; SafeBoosC-III Follow-Up Writing Group for the SafeBoosC-III Follow-Up Collaborator Group. Cerebral oximetry in extremely preterm infants: 2-year follow-up of the SafeBoosC-III randomized clinical trial. *JAMA Pediatr*. Published online April 20, 2026. doi:10.1001/jamapediatrics.2026.1066

eAppendix. List of Investigators

eMethods.

eTable 1. Death or Severe Brain Injury at 36 Weeks' Postmenstrual Age and at 2-Year Follow-Up in Included and Excluded Sites

eTable 2. Causes of Death Beyond 36 Weeks' Postmenstrual Age

eTable 3. Per-Protocol Analysis

eTable 4. Multiple Imputation Analysis on Death or Moderate or Severe Neurodevelopmental Disability

eTable 5. Best-Worst and Worst-Best Case Analysis on Death or Moderate or Severe Neurodevelopmental Disability

eTable 6. Exclusion of Informal Assessment (Tier 3) for the Coprimary Outcome Death or Moderate or Severe Neurodevelopmental Disability

eTable 7. Number of Events in Coprimary Outcome Death or Moderate or Severe Neurodevelopmental Disability for Informal Assessments (Tier 3)

eTable 8. Comparison of Sites With High Follow-Up Rates (≥90%) vs Low Follow-Up Rates (<90%) for the Coprimary Outcome Death or Moderate or Severe Neurodevelopmental Disability

eTable 9. Comparison of Sites With High Follow-Up Rates (≥90 vs Low Follow-Up Rates (<90%) for the Coprimary Outcome Bayley III/IV Cognitive Score

eTable 10. Generalized Estimation Equation (Twin Sensitivity Analysis)

eTable 11. Proportion of Missingness Between Cerebral Oximetry Group and Experimental Group for the 2 Coprimary Outcomes

eTable 12. Analysis of all PARCA-R Nonverbal Cognitive Scores Including Extrapolated Scores

© 2026 Rasmussen MIS et al. *JAMA Pediatrics*.

eTable 13. Death or Moderate or Severe Neurodevelopmental Disability With PARCA-R Nonverbal Cognitive Extrapolated Scores

eTable 14. Follow-Up Times of Randomized Infants

eTable 15. Clinical Data Based on Information From Health Care Professional

eTable 16. Parental Education Level Based on ISCED Classification

eTable 17. Number of Randomizations per Site

eFigure 1. Prioritization of Data for the Coprimary Outcome Moderate or Severe Neurodevelopmental Disability

eFigure 2. Random Effect Meta-Analysis for Coprimary Outcome Moderate or Severe Neurodevelopmental Disability and Bayley III/IV Cognitive Score

eFigure 3. Extract From the Electronic Case Report Form for 2-Year Clinical Data

eFigure 4. Parental Questionnaire Used for the SafeBoosC-III Follow-Up Study

eReferences.

This supplementary material has been provided by the authors to give readers additional information about their work.

List of investigators

eSection 1: Corresponding author and trial manager

Marie Isabel Skov Rasmussen, MD, Department of Neonatology, Copenhagen University Hospital – Rigshospitalet, Blegdamsvej 9, 2100 Copenhagen Ø, Denmark, marie.isabel.skov.rasmussen@regionh.dk

eSection 2: Coordinating investigator

Gorm Greisen, MD, DMSc, Emeritus Professor, Department of Neonatology, Copenhagen University Hospital – Rigshospitalet, Blegdamsvej 9, 2100 Copenhagen Ø, Denmark, gorm.greisen@regionh.dk

eSection 3: Executive committee

Adelina Pellicer, Department of Neonatology, La Paz University Hospital, Madrid, Spain (national coordinator and principal investigator); **Christian Gluud**, Copenhagen Trial Unit, Centre for Clinical Intervention Research, The Capital Region, Copenhagen University Hospital – Rigshospitalet, Copenhagen, Denmark and Department of Regional Health Research, The Faculty of Health Sciences, University of Southern Denmark, Odense, Denmark (trialist); **Eugene Dempsey**, Infant Centre and Department of Paediatrics and Child Health, University College Cork, Cork, Ireland (national coordinator and principal investigator); **Gorm Greisen**, Department of Neonatology, Copenhagen University Hospital – Rigshospitalet, Copenhagen, Denmark (coordinating investigator); **Janus Christian Jakobsen**, Copenhagen Trial Unit, Centre for Clinical Intervention Research, The Capital Region, Copenhagen University Hospital – Rigshospitalet, Copenhagen, Denmark and Department of Regional Health Research, The Faculty of Health Sciences, University of Southern Denmark, Odense, Denmark (trialist); **Jonathan Mintzer,** Department of Pediatrics, Division of Newborn Medicine, Mountainside Medical Center, Montclair, NJ, USA (national coordinator); **Marie Isabel Skov Rasmussen**, Department of Neonatology, Copenhagen University Hospital – Rigshospitalet, Copenhagen, Denmark (trial manager); **Mathias Lühr Hansen**, Department of

Neonatology, Copenhagen University Hospital – Rigshospitalet, Copenhagen, Denmark and Copenhagen Trial Unit, Centre for Clinical Intervention Research, The Capital Region, Copenhagen University Hospital – Rigshospitalet, Copenhagen, Denmark (trialist); **Simon Hyttel-Sørensen**, Department of Intensive Care, Copenhagen University Hospital – Rigshospitalet, Copenhagen, Denmark (national coordinator).

eSection 4: Steering committee

Adelina Pellicer, Department of Neonatology, La Paz University Hospital, Madrid, Spain (national coordinator and principal investigator); **Anne Marie Heuchan**, Department of Neonatal Medicine, Royal Hospital for Children, Glasgow, United Kingdom (national coordinator and principal investigator); **Christian Gluud**, Copenhagen Trial Unit, Centre for Clinical Intervention Research, The Capital Region, Copenhagen University Hospital – Rigshospitalet, Copenhagen, Denmark and Department of Regional Health Research, The Faculty of Health Sciences, University of Southern Denmark, Odense, Denmark (trialist); **Cornelia Hagmann**, Pediatric Intensive Care and Neonatology, Children's University Hospital of Zurich, Zurich, Switzerland; (national coordinator); **Ebru Ergenekon**, Division of Newborn Medicine, Gazi University Hospital, Ankara, Turkey (national coordinator and principal investigator); **Eugene Dempsey**, Infant Centre and Department of Paediatrics and Child Health, University College Cork, Cork, Ireland (national coordinator and principal investigator); **Gabriel Dimitriou**, NICU, Department of Pediatrics, Patras Medical School, Patras, Greece (national coordinator and principal investigator); **Gerhard Pichler**, Department of Pediatrics, Medical University of Graz, Graz, Austria (national coordinator and principal investigator); **Gorm Greisen**, Department of Neonatology, Copenhagen University Hospital – Rigshospitalet, Copenhagen, Denmark (coordinating investigator); **Gunnar Naulaers**, Department of Development and Regeneration KU Leuven, Leuven, Belgium (national coordinator); **Hans Fuchs**, Center for Pediatrics, Department of Neonatology, Medical Center, University of Freiburg, Germany (national coordinator and principal investigator); **Jakub Tkaczyk**, Department of Neonatology, University Hospital Motol, Prague, Czech Republic (national coordinator and principal investigator); **Janus Christian Jakobsen**, Copenhagen Trial Unit, Centrefor Clinical Intervention Research, The Capital Region,

Copenhagen University Hospital –Rigshospitalet, Copenhagen, Denmark and Department of Regional Health Research, The Faculty of Health Sciences, University of Southern Denmark, Odense, Denmark (trialist); **Jonathan Mintzer**, Department of Pediatrics, Division of Newborn Medicine, Mountainside Medical Center, Montclair, NJ, USA (national coordinator); **Marie Isabel Skov Rasmussen**, Department of Neonatology, Copenhagen University Hospital – Rigshospitalet, Copenhagen, Denmark (trial manager); **Mathias Lühr Hansen**, Department of Neonatology, Copenhagen University Hospital – Rigshospitalet, Copenhagen, Denmark and Copenhagen Trial Unit, Centre for Clinical Intervention Research, The Capital Region, Copenhagen University Hospital –Rigshospitalet, Copenhagen, Denmark (trialist); **Monica Fumagalli**, Fondazione IRCCS Ca' Granda Ospedale Maggiore Policlinico, Milan, Italy (national coordinator and principal investigator); **Saudamini Nesargi**, Department of Neonatology, St.John's Medical College Hospital, Karnataka, India (national coordinator); **Simon Hyttel-Sørensen**, Department of Intensive Care, Copenhagen University Hospital – Rigshospitalet, Copenhagen, Denmark (national coordinator); **Siv Fredly**, Department of Neonatology, Oslo University Hospital Oslo, Norway (national coordinator and principal investigator); **Tomasz Szczapa**, 2nd Department of Neonatology, Neonatal Biophysical Monitoring and Cardiopulmonary Therapies Research Unit, Chair of Neonatology, Poznan University of Medical Sciences, Poznan, Poland (national coordinator and principal investigator).

eSection 5: Copenhagen Trial Unit

Copenhagen Trial Unit, Centre for Clinical Intervention Research, The Capital Region, Copenhagen University Hospital – Rigshospitalet, Blegdamsvej 9, 2100 Copenhagen, Denmark.

Trialists: Christian Gluud, MD, DMSc, Janus Christian Jakobsen, MD, DMSc and Mathias Lühr Hansen, MD, PhD

Data manager: Janus Engstrøm, BCompSc

Statisticians: Markus Harboe Olsen, MD, PhD and Janus Christian Jakobsen, MD, DMSc

eSection 6: List of principal investigators and blinded assessors

Austria

University Hospital Graz, Graz: Gerhard Pichler (national coordinator and principal investigator), Elisabeth Pichler-Stachl (blinded assessor).

Belgium

University Hospital Leuven, Leuven: Gunnar Naulaers (national coordinator), Liesbeth Thewissen (principal investigator), Els Ortibus (blinded assessor); **Clinique CHC Montlegia**, Liege: Pierre Maton (principal investigator), Nancy Laval (blinded assessor); **AZ St. Jan Bruges**, Bruges: Luc Cornette (principal investigator), Anna Oostra (blinded assessor); **Grand Hospital de Charleroi,** Charleroi: Chantal Lecart (principal investigator), Marie-Julie Debuf (blinded assessor).

Czech Republic

University Hospital Motol, Prague: Jakub Tkaczyk (national coordinator and principal investigator), Katarina Ticha (blinded assessor); **The Institute for the Care of Mother and Child**, Prague: Jan Sirc (principal investigator), Zuzana Matějková (blinded assessor).

Denmark

Copenhagen University Hospital – Rigshospitalet, Copenhagen: Gitte Hahn (principal investigator), Gorm Greisen (blinded assessor); **Odense University Hospital,** Odense: Anja Klamer (principal investigator), Gorm Greisen (blinded assessor); **Aarhus University Hospital,** Aarhus: Sofie Sommer Hedegaard (principal investigator and blinded assessor); **Aalborg University Hospital,** Aalborg; Lars Bender (principal investigator), Alexander Scheid (blinded assessor).

Germany

Medical Center, University of Freiburg, Freiburg: Hans Fuchs (national coordinator and principal investigator)

Greece

University of Patras General Hospital, Patras: Gabriel Dimitriou (national coordinator and principal investigator), Aikaterini Nourloglou (blinded assessor); **Ippokrateion General Hospital of Thessaloniki**, Thessaloniki: Kosmas Sarafidis (principal investigator), Vasiliki Kourou (blinded assessor); **"Alexandra" University and State Maternity Hospital,** Athens: Evangelia Papathoma (principal investigator and blinded assessor); **University Hospital of Heraklion,** Heraklion; Eleftheria Hatzidaki (principal investigator), Nicole Hilda Anagnostatou (blinded assessor).

India

St. John's Medical College Hospital, Karnataka: Saudamini Nesargi (national coordinator), Shashidhar Appaji Rao (principal investigator), Anjali Raj (blinded assessor).

Ireland

INFANT Research Centre, University College Cork, Cork: Eugene Dempsey (national coordinator and principal investigator), Iyshwarya Stapleton (blinded assessor); **The Rotunda Hospital,** Dublin: Afif EL Khuffash (principal investigator), Iyshwarya Stapleton (blinded assessor); **Coombe Woman and Infant University Hospital,** Dublin; Jan Miletin (principal investigator), Jsun Wong (blinded assessor); **National Maternity Hospital, Holles Street,** Dublin: Caitríona Ní Chathasaigh (principal investigator), Jyothsna Purna (blinded assessor).

Italy

Fondazione IRCCS Ca' Granda Ospedale Maggiore Policlinico, Milan: Monica Fumagalli (national coordinator and principal investigator), Camilla Fontana (blinded assessor); **Fondazione Policlinico Universitario A. Gemelli IRCCS,** Roma: Francesca Serrao (principal investigator and blinded

assessor**); Struttura Complessa di Neonatologia, Ospedale Sant'Anna, Città della Salute e della Scienza di Torino,** Turin: Tatiana Boetti (principal investigator), Anna Zoraide Patria (blinded assessor).

Norway

Oslo University Hospital, Oslo: Siv Fredly (national coordinator and principal investigator), Tone Nordvik (blinded assessor).

Poland

Poznan University of Medical Sciences, Poznan: Tomasz Szczapa (national coordinator and principal investigator), Roksana Malak (blinded assessor); **Centrum Medyczne "Ujastek" Sp. z o.o,** Krakow: Beata Rzepecka (principal investigator), Elzbieta Rafinska-Wazny (blinded assessor); **Jagiellonian University Medical College,** Krakow: Agnieszka Ochoda-Mazur (principal investigator and blinded assessor); **Medical University of Warsaw,** Warsaw: Renata Bokiniec (principal investigator), Justyna Fiałkowska (blinded assessor); **Specialist Hospital No. 2,** Bytom: Sylwia Marciniak (principal investigator), Kasia Szczepanska (blinded assessor); **Wroclaw Medical University,** Wroclaw: Barbara Krolak-Olejnik (principal investigator), Paulina Gaweł (blinded assessor); **Collegium Medicum in Bydgoszcz Nicolaus Copernicus University in Torun**, Bydgoszcz: Iwona Sadowska-Krawczenko (principal investigator), Iga Rupniak (blinded assessor).

Spain

La Paz University Hospital, Madrid: Adelina Pellicer (national coordinator and principal investigator), Malaika Cordeiro Alcaine (blinded assessor); **Hospital Clinic Barcelona,** Barcelona: Miguel Alsina-Casanova (principal investigator), Marta Teresa-Palacio (blinded assessor); **12 de Octubre University Hospital,** Madrid: Salvador Piris-Borregas (principal investigator), Maria Palomares Eraso (blinded assessor); **Hospital Sant Joan De Deu,** Barcelona: Ruth del Rio Florentino (principal investigator), Thais Agut Quijano (blinded assessor); **Puerta del Mar University Hospital,** Cadiz: Pamela Zafra (principal investigator), Isabel Benavente Fernández (blinded assessor); **Hospital Clinico San Carlos,**

Madrid: Luis Arruza (principal investigator), Isabel Cuellar Flores (blinded assessor); **Marques de Valdecilla University Hospital,** Santander: Isabel de las Cuevas (principal investigator), Rosa Ayesa Arriola (blinded assessor); **Miguel Servet University Hospital**, Zaragoza: Itziar Serrano-Vinuales (principal investigator), Pilar Abenia (blinded assessor).

Switzerland

University Hospital Zürich, Zürich: Tanja Karen (principal investigator), Claudia Knöpfli (blinded assessor); **Children's Hospital Lucerne**, Lucerne: Martin Stocker (principal investigator), Barbara Imboden (blinded assessor); **Children's University Hospital of Geneva,** Geneva: Francisca Barcos-Munoz (principal investigator), Cristina Borradori Tolsa (blinded assessor); **University Hospital Center of Lausanne,** Lausanne: Juliane Schneider (principal investigator), Myriam Bickle Graz (blinded assessor).

Turkey

Gazi University Hospital, Ankara: Ebru Ergenekon (national coordinator and principal investigator), Sebnem Soysal (blinded assessor); **Marmara University Research and Educational Hospital,** Istanbul: Asli Cinar Memisoglu (principal investigator), Sinem Gulcan Kersin (blinded assessor); **Bursa Uludag University Hospital,** Bursa: Hilal Ozkan (principal investigator), Mustafa Bostanci (blinded assessor); **Kanuni Sultan Süleyman Training and Research Hospital,** Istanbul: Merih Cetinkaya (principal investigator), Halime Sema Can Buker (blinded assessor); **Ankara City Hospital,** Ankara: Serife Suna Oguz (principal investigator), Gülsüm Kadıoğlu (blinded assessor); **Basaksehir Cam and Sakura City Hospital,** Istanbul: Beril Yasa (principal investigator), Halime Sema Can Buker (blinded assessor).

United Kingdom

Royal Hospital for Children, Glasgow: Anne Marie Heuchan (national coordinator) Sarah Farquharson (principal investigator and blinded assessor); **University Hospital Wishaw,** Wishaw: Mahmoud Montasser (principal investigator), Ani Majeed (blinded assessor).

United States of America

University of Utah Hospital, Salt Lake City: Mariana Baserga (principal investigator), Trisha Marchant (blinded assessor); **Loma Linda University Children's Hospital,** Los Angeles: Nicole Kraus (principal investigator and blinded assessor); **UT Southwestern Medical Center,** Dallas: Lina Chalak (principal investigator), Katelyn Hoffer (blinded assessor); **St. Louis Children's Hospital,** St Louis: Zachary Vesoulis (principal investigator), Amanda Duncan (blinded assessor).

Supplementary Methods

eMethods 1: List of excluded sites and reason for exclusion

In December 2022, the steering committee voted on criteria for site eligibility in the SafeBoosC-III follow-up study. This was prompted by two sites that had exceeded six months since their first participant reached 24 months of corrected age and had made no progress on preparations. The committee emphasized that if a site does not participate, infants from such a site should not be included in the follow-up study and therefore, not count as missing data. If a site initiates follow-up, unassessed infants from such sites should count as missing data. With 15 out of 20 votes in favor, it was decided that if six months passed since the first participant reached 24 months the trial manager and coordinating investigator would inform the national coordinator and a decision was taken on whether to exclude the site. One site was excluded after having started the follow-up study. The principal investigator and blinded assessor from the site would not be co-authors on final publications.

**Sites withdrawn/excluded**	**Reason**
CHU Tivoli, La Louviere, Belgium	Due to non-compliance with Belgian law and continuous lack of communication, it was decided to exclude the site in November 2024.
Children's Hospital affiliated to Fudan Univesity, Shanghai, China	Chinese children are not routinely followed up, and the loss to follow-up rate is generally high. As all Chinese centers had surpassed the established threshold of 6 months and communication remained limited, the steering committee unanimously approved the exclusion of all Chinese centers in January 2024.
Guanxi Maternal and Child Healthcare Hospital, Nanning, China	Idem
Hainan Women and Children’s Medical Center, Hainan, China	Idem
Guangzhou Women and Children's Medical Center, Guangzhou, China	Idem

The People's Hospital of Dehong autonomous prefecture, Dehong, China	Idem
Xiamen Children's Hospital, Xiamen, China	Idem
The Children’s Hospital Zheijang, Hangzhou, China	Idem
Longgang District Central Hospital of Shenzhen, Shenzhen, China	Idem
Ospedale Fillipo del Ponte, Varese, Italy	Withdrew due to no dedicated researchers who could apply to ethics committee and follow the study protocol. The researchers involved in SafeBoosC-III moved to other hospitals.
Centre of Medical Postgraduate Education Warsaw, Warsaw, Poland	Excluded due to closure of NICU and no possibility to follow up participants.
H. Universitario Juan XXIII De Tarragona, Tarragona, Spain	Due to continuous lack of communication and no progress in study preparations, it was decided to exclude the site in February 2023.
H. Universitario Virgen de Las Nieves, Granada, Spain	Due to continuous lack of communication and no progress in study preparations, it was decided to exclude the site in February 2023.
Hospital de Cruces, Bilbao, Spain	Due to continuous lack of communication and no progress in study preparations, it was decided to exclude the site in July 2024.

eMethods 2: Blinding procedures

Due to the nature of the trial intervention, both children and their parents were aware of their treatment allocation. However, a blinded assessor who remained blinded to group allocation, reviewed all relevant healthcare records, performed classifications, and entered data into the electronic case report forms. To maintain blinding during the outcome assessment, the principal investigator and blinded assessor at each site developed a local blinding procedure outlining the workflow. This procedure was reviewed and approved by Marie Rasmussen and Gorm Greisen, prior to the study's commencement. As a result, all outcome data, except for the parental-reported questionnaires, were assessed in a blinded manner.

eMethods 3: Description of the 3-tier model for data collection

A 3-tier model for prioritization was applied using formal clinical data, parental questionnaires and informal assessments. This approach was chosen to minimize loss to follow-up, assuming that little data was better than no data at all. For an overview of the 3-tier model please see Figure S1.

Moderate-or-severe neurodevelopmental disability was defined as:

- **Diagnosis of motor impairment**: cerebral palsy with functional impairment corresponding to a GMFCS ≥2
- **Diagnosis of visual impairment**: defined as moderately reduced vision or worse (e.g., blindness in one eye with good vision in the other eye, or blindness, or the ability to only perceive light or light-reflecting objects).
- **Diagnosis of hearing impairment**: defined as hearing loss corrected with aids, partial hearing loss uncorrected by aids, or no useful hearing even with aids.
- **Cognitive impairment**:
  - Bayley-III/IV cognitive score <85 (1st priority)
  - Any developmental assessment < -2SD (including the PARCA-R) (2nd priority)
  - If none of the above were available, blinded assessment of health care records from 12 months corrected age and onward, concluding the child had cognitive impairment equivalent to moderate-or-severe neurodevelopmental disability (3rd priority).

Informal assessment of moderate-or-severe neurodevelopmental disability

When there were no relevant health care records from corrected age 18-30 months, the blinded assessor accessed all available health care records from corrected age 12 months onward and informally assessed them to determine whether the child had moderate-or-severe neurodevelopmental disability.

The full electronic case report form can be found in figure 3.

Classification of moderate-or severe neurodevelopmental disability based on parental questionnaire (in cases where components of the dichotomous co-primary outcome are unknown or if it is not possible for the blinded assessor to assess neurodevelopment based on the health care records):

YES to cerebral palsy: A doctor has said the child has cerebral palsy

The child CANNOT walk independently at two years of age

NO to cerebral palsy: A doctor has said the child has cerebral palsy

The child CAN walk independently at two years of age

NO to cerebral palsy: A doctor has NOT said the child has cerebral palsy

The child CANNOT walk independently at two years of age

NO to cerebral palsy: A doctor has NOT said the child has cerebral palsy

The child CAN walk independently at two years of age

YES to visual impairment: The child is blind in one or two eyes OR has poor vision even with glasses

NO to visual impairment: The child has visual problems

The child wears glasses

The child has a squint (the eyes look in different directions)

YES to hearing impairment: The child wears aids or cochlear implants

The child will be categorised as no moderate-or-severe NDD if all components are ”NO”.

The child will be categorised as “not able to classify”, if one or more components are unknown and none are positive. Thereby all four components of the primary outcome must be “no”, to be classified as no moderate-or-severe NDD.

The full parental questionnaire can be found in figure 4.

Example 1:

- Tier 1: From health care records: no to cerebral palsy, hearing impairment, visual impairment, Bayley III score 90
- Tier 2: From parental questionnaire: no to cerebral palsy, hearing impairment, PARCA-R score of 85, yes to vision impairment.

= The child will be classified as NO in “moderate-or-severe neurodevelopmental disability”

Example 2:

- Tier 1: From health care records: yes to cerebral palsy, no to hearing impairment and visual impairment, no Bayley assessment or other formal test of neurodevelopment
- Tier 2: none available
- Tier 3: cognitive informal assessment classified as no in “moderate-or-severe neurodevelopmental disability”

  = The child will be classified as YES in “moderate-or-severe neurodevelopmental disability”

Example 3:

- Tier 1: From health care records: no to cerebral palsy, unknown to visual impairment, hearing impairment and no formal test of neurodevelopment
- Tier 2: From parental questionnaire: no vision impairment, unknown to hearing, cerebral palsy and no PARCA-R NVC score
- Tier 3: cognitive informal assessment classified as no in “moderate-or-severe neurodevelopmental disability”

= The child will be “not able to classify” due to missing hearing component = lost to follow up

eMethods 4: Central monitoring procedures

A central monitoring plan was designed based on the monitoring plan from the SafeBoosC-III trial.[1] The monitoring plan was divided into two parts 1) missing data monitoring and 2) monitoring of quality deficiencies and deviations.

Part 1 – missing data monitoring: For each site, missing data for both clinical data and parental questionnaires were reported. Data were considered missing if the “blinded 2-year follow-up” data entry was not completed by 24 months of corrected age plus one month. Although data were not technically missing until after 30 months of corrected age, this threshold served as a reminder to investigators to complete the data entry in a timely manner. Parental questionnaires, hosted on REDCap, were standardised to be filled out between 23.3-27.5 months of corrected age. The completion rates were reported for each site in the monthly

newsletter circulated to all investigators and uploaded to ‘safeboosc.eu’. Identified study IDs with missing data were sent directly to the relevant primary investigators by the study manager.

Part 2 – quality deficiencies and deviations: To identify sites with noteworthy data deviations, monitoring of data quality deficiencies was conducted when the last participant turned 24 months of corrected age and again at 30 months of corrected age. The focus was on identifying noteworthy data deviations such as outliers, systematic deviations due to misunderstandings, and suspected fabricated data. Quantitative measures were analyzed for outliers. Systematic deviations were reviewed, such as above-average numbers of participants lost to follow-up or inconsistencies in cognitive assessment scores. Inputted dates for time of assessments were performed as well. Any identified issues prompted direct communication with investigators for verification or correction. Additionally, data were examined for unexpected distributions or variances in binary data. The results from this monitoring were logged in a ‘central monitoring log’ by the monitoring group, ensuring that any issues were documented and addressed appropriately.

eMethods 5: Extrapolation of PARCA-R NVC scores

To extrapolate the PARCA-R Non-Verbal Cognitive (NVC) scores outside the normative timeframe (23.5-27.5 months corrected age), we used simple linear regression between age and NVC score within the norm timeframe. The regression analysis was applied to generate extrapolated values. The extrapolated scores were not used for the primary analysis of moderate-or-severe neurodevelopmental disability, but included in a sensitivity analysis (Table S11).

eMethods 6: Types of cognitive informal assessments and how many used

Formal neurodevelopmental tests assess various aspects of child development and varies greatly across participating sites in the SafeBoosC-III follow up study. These differences include the types of assessments, their timing, and result interpretation methods. However, cognitive assessments are central to identifying developmental disability, but can measure different things. To make sure we only include tests that measure a cognitive domain when comparing outcomes, a predefined list of tests were determined to be used for

classification of the co-primary outcome of death or moderate-to-severe neurodevelopmental disability.[2-11] The selection of tests, and their priority were done by Professor Gorm Greisen and MD, PhD student Marie Isabel Skov Rasmussen in August 2024, in a blinded manner and prior to data analysis. An analysis excluding informal cognitive assessments in the analysis of the co-primary outcome moderate-or-severe neurodevelopmental disability can be found in Table S5.

Test Name	Description	Scoring	Cognitive Assessment?	Number of children classified as YES to moderate-to-severe neurodevelopmental disability using another cognitive test
**ASQ-24**	Parental questionnaire. The Ages & Stages Questionnaires, 24-Month Version, screens developmental milestones in young children at 24 months of age.	Scores are based on caregiver responses and compared to developmental milestones; identifies areas of concern.	Yes, it is a developmental screening tool.	Cerebral oximetry group: 8<br>Usual care group: 11
**Bayley-III Cognitive Score**	The Cognitive Scale assesses cognitive abilities such as problem-solving, memory, and learning in infants and toddlers.	Raw scores are converted to standard scores (mean = 100, SD = 15) and percentiles; part of a comprehensive developmental assessment.	Yes, it focuses specifically on cognitive abilities.	Cerebral oximetry group: 0<br>Usual care group: 1
**Developmental Profile 3 (DP-3)**	Parental questionnaire. A comprehensive assessment tool evaluating physical, adaptive, social-emotional, cognitive, and communication development in children from birth to age 12.	The DP-3 assesses five key developmental domains: Physical, Adaptive, Social-Emotional, Cognitive, and Communication, which are gathered to a DQ with a mean of 100 (SD 15).	Yes, assesses multiple developmental domains, not just cognitive skills.	Cerebral oximetry group: 1<br>Usual care group: 2

**Denver Developmental Screening Test II**	Primarily clinician. A screening tool used to identify developmental delays by assessing milestones in personal-social, fine motor-adaptive, gross motor, and language areas.	No numeric score. Domains: Personal-Social, Fine Motor-Adaptive, Gross Motor, Language. Tasks are scored as pass, fail, or not attempted; results are compared to normative data to identify potential delays.	Yes, but it is primarily a screening tool.	Cerebral oximetry group: 1<br>Usual care group: 0
**Battelle Developmental Inventory: Cognitive**	A part of the Battelle Developmental Inventory, it evaluates cognitive skills in young children, focusing on problem-solving and reasoning abilities.	Provides standard scores (mean = 100, SD = 15) and age equivalents, part of a broader developmental assessment.	Yes, it focuses specifically on cognitive abilities.	Cerebral oximetry group: 0<br>Usual care group: 2
**Brunet-Lézine**	A developmental assessment tool that evaluates cognitive, motor, language, and social development in children from birth to 6 years.	Provides developmental quotients and age equivalents based on direct testing and caregiver reports. DQ with mean score of 100 and SD 15.	Yes, it includes cognitive development assessments.	Cerebral oximetry group: 1<br>Usual care group: 0
**Haizea-Llevant**	A developmental scale used to assess motor, cognitive, and language skills in children from birth to 6 years old.	Results are used to generate developmental profiles and identify areas needing intervention.	Yes, it includes cognitive development assessments.	Cerebral oximetry group: 0<br>Usual care group: 1
**Griffiths-3**	The Griffiths III is a comprehensive developmental assessment for children from birth to 8 years, covering multiple developmental domains.	Provides scores for various developmental domains and an overall developmental quotient (mean 100, SD 15)	Yes, it assesses multiple domains, including cognitive skills.	Cerebral oximetry group: 5<br>Usual care group: 10
**SOGs (Schedule of**	Clinician screening. A developmental	Provides scores for domains: Passive	It assesses multiple	Cerebral oximetry group: 1

**Growing Skills)**	assessment tool for children from birth to 6 years, evaluating skills across various domains including communication and motor skills.	Posture, Active Posture, Locomotor, Manipulative, Visual, Hearing and Language, Speech and Language, Interactive Social and Self-Care Social. No DQ.	domains, not just cognitive skills.	Usual care group: 1

eMethods 7: Overview of sensitivity analyses

The **per-protocol analysis** (Table S3) only included infants that adhered to the interventions. In the cerebral oximetry group, adherence was defined as less than 14 hours of missing cerebral oximetry monitoring in the intervention period, for infants surviving beyond 72 hours after birth. For infants in the usual care group, adherence was defined as no cerebral oximetry monitoring or blinded cerebral oximetry monitoring (infants in the usual care group could undergo blinded cerebral oximetry monitoring if they were enrolled in other clinical studies where recording of cerebral oxygenation was necessary).

The **random-effects meta-analysis** (Figure 2a and 2b) included the intention-to-treat population and used the Mantel-Haenszel method.

The **generalized estimation equation analysis** (Table S10) also included the intention-to-treat population and relative risk was derived using G-computation. This analysis was conducted to consider non-independence of the primary outcome for multiple births.

The **high/low follow up rate analysis** (Table S8) compared sites with high follow-up rates (>90%) to those with lower follow-up rates (<90%) to examine potential differences in effect estimates.

The **informal assessment (Tier 3) exclusion analysis** of moderate-or-severe neurodevelopmental disability (Table S6) was conducted to assess impact on the overall results.

The **PARCA-R extrapolated analysis** (Table S12 and Table S13) included all PARCA-R scores, extrapolating data from questionnaires completed outside the standardized timeframe.

eMethods 8: Protocol deviations

Primary analysis

The primary analyses of all outcomes were based on the intention-to-treat population, using complete case mixed-effect linear regression for continuous outcomes and mixed-effect logistic regression for dichotomous outcomes. Initially, a random effects model was specified, including ‘site’ as a random effect and ‘gestational age’ and ‘group allocation’ as fixed effects. However, due to collinearity issues, a fixed effect model was chosen to simplify the analysis, thus constituting a protocol deviation.

Blinding issue in one site

Following the completion of the primary analysis, one Greek site reported that the principal investigator, rather than the blinded assessor, had conducted the outcome reporting for the follow-up study. The investigator reported that whereas she had initially not been blinded to group allocation during the execution of the trial, she had forgotten group allocation in all children over time. The site included 15 eligible infants in the follow-up study.

eMethods 9: Changes to the electronic case report form

A full extract of the electronic case report form can be found in figure 3.

On November 29, 2022, the electronic case report form was updated adding the field F14b, "Was the Bayley corrected," and F14a was clarified as "composite score", instead of ”score”.

On December 15, 2022, a new info text box was added to F14b with the following text: "The composite score has a mean of 100 and a standard deviation of 15".

On January 15th, 2023, in F15d, the text box was added "*As this test is used in your clinical practice, would the child's score be indicative of neurodevelopmental disability (we usually use below -2 standard deviations of the norm to classify disability*)?"

eMethods 10: Overview of central funding

The sponsor of this project is The Capital Region of Denmark and the sponsor representative and coordinating investigator, professor of neonatology Gorm Greisen, is the initiator of the SafeBoosC-III project. The Capital Region of Denmark and professor Gorm Greisen has no financial interest in the results of the trial, nor in the oximetry devices. The SafeBoosC-III trial center was supported by unconditional and unrestricted grants from the Elsass Foundation (DKK 3,300,000); and Aage and Johanne Louis-Hansen Foundation (DKK 1,950,000.); the Svend Andersen Foundation (DKK 1,000,000) which covered the costs of

the execution of the trial as well as the 36 week and 2-year follow-up. The funding sources had no role in the design, execution, analyses, interpretation of data or decision to the results of this trial.

eMethods 11: Overview of local financial funding

Local expenses for the 36 week follow up of the SafeBoosC-III trial are previously outlined.[12] Local expenses specifically for the SafeBoosC-III follow up study are outlined here:

**Site**	**Funding**
Copenhagen University Hospital – Rigshospitalet	Received funding to pay MD, PhD student Marie Isabel Skov Rasmussen to conduct Bayley assessments, as this is not clinical routine. Funding covered materials and supervision.
All Belgian sites	KCE (Belgian Health Care Knowledge Centre) provides equipment, Good Clinical Practice resources and money per patient, through the KCE Trials Programme (study ID KCE18-1144).
La Paz University Hospital Madrid, Spain	FUNDACION MUTUA MADRILEÑA supported funding for a research fellow.

Supplementary Tables

eTable 1: Death or severe brain injury at 36 weeks' postmenstrual age and at 2-year follow-up in included and excluded sites.

	**Participants included in 2-year follow-up study** *n=1438*	**Participants not included in 2-year follow-up study** *n=163*
Death at 36 weeks' postmenstural age, no/total no. (%)	297/1438 (20.2)	27/141 (19.1)
Severe brain injury at 36 weeks' postmenstural age, no/total no. (%)	344/1420 (24.2)	29/142 (20.4)

eTable 2: Causes of death beyond 36 weeks postmenstrual age

	**Cerebral oximetry** *n=12*	**Usual care** *n=18*
Perinatal brain damage related, no.	1	1
Malformation/congenital disease, no.	1	1
Other prematurity related, no.	8	14
Unknown, no.	2	2

Reasons for death were reported in a free-text entries in the electronic case report form and were thereafter categorized in a blinded manner

eTable 3: Per protocol analysis

	**Cerebral oximetry** *n = 590*	**Usual care** *n = 655*	**Logistic regression** *RR (97.5%CI)*
Death or moderate-or-severe neurodevelopmental disability, n (%)	284 (48.1)	313 (47.8)	1.03 (0.90-1.18)

	**Cerebral oximetry** *n = 236*	**Usual care** *n = 280*	**Mixed effects linear regression** *Mean difference (97.5%CI)*
Bayley III/IV cognitive score, mean (97.5% CI)	92.5 (89.96-94.96)	93.4 (91.04-95.68)	0.50 (-2.63-3.65)

eTable 4: Multiple imputation analysis on death or moderate-or-severe neurodevelopmental disability

	**Cerebral oximetry** *n = 697*	**Usual care** *n = 741*	**Logistic regression** *RR (97.5%CI)*
Death or moderate-or-severe neurodevelopmental disability, n (%)*	330 (47.3)	352 (47.5)	0.98 (0.88-1.10)

*Multiple imputation assumed data was missing at random, and chained equation multiple imputation was used to predict missing values based on observed data. Variables for imputation include gestational age, sex, respiratory treatment, intraventricular hemorrhage, retinopathy of prematurity, bronchopulmonary dysplasia, and necrotizing enterocolitis.

eTable 5: Best-worst and worst-best case analysis on death or moderate-or-severe neurodevelopmental disability

	**Cerebral oximetry** *n = 697*	**Usual care** *n = 741*	**Logistic regression** *RR (97.5%CI)*
Death or moderate-or-severe neurodevelopmental disability, n (%)	292 (41.9)	393 (53.0)[a]	0.78 (0.70-0.87)
	**Cerebral oximetry** *n = 697*	**Usual care** *n = 741*	**Logistic regression** *RR (97.5%CI)*
Death or moderate-or-severe neurodevelopmental disability, n (%)	369 (52.9)	321 (43.3)[b]	1.21 (1.08-1.35)

[a] Death or moderate-or-severe neurodevelopmental disability/poor outcome for all infants with missing data in the cerebral oximetry group, and alive without moderate-or-severe neurodevelopmental disability /good outcome for all infants with missing data in the usual care group

[b]Alive without moderate-or-severe neurodevelopmental disability/good outcome for all infants with missing data in the cerebral oximetry group, and death or moderate-or-severe neurodevelopmental disability/poor outcome for all infants with missing data in the usual care group.

eTable e6: Exclusion of informal assessment (Tier 3) for the co-primary outcome death or moderate-or-severe neurodevelopmental disability

	**Cerebral oximetry** *n = 620*	**Usual care** *n = 669*	**Logistic regression** *RR (97.5%CI)*
Death or moderate-or-severe neurodevelopmental disability, n (%)	292 (47.1)	321 (48.0)	0.96 (0.85-1.07)

eTable 7: Number of events in co-primary outcome death or moderate-or-severe neurodevelopmental disability for informal assessments (Tier 3)

	**Cerebral oximetry** *n = 60*	**Usual care** *n = 73*	**Logistic regression** *RR (97.5%CI)*
Death or moderate-or-severe neurodevelopmental disability, n (%)	5 (8.3)	14 (19.2)	0.58 (0.25-1.37)

eTable e8: Comparison of sites with high follow up rates (≥90%) vs low follow up rates (<90%) for the co-primary outcome death or moderate-or-severe neurodevelopmental disability

Sites with high follow up rate (≥90%)	**Cerebral oximetry** *n = 396*	**Usual care** *n = 441*	**Logistic regression** *RR (97.5%CI)*
Death or moderate-or-severe neurodevelopmental disability, n (%)	182 (47.7)	206 (48.7)	0.96 (0.83-1.11)
- missing, n (%)	15 (3.8)	18 (4.1)	
Sites with low follow up rate (<90%)	**Cerebral oximetry** *n = 301*	**Usual care** *n = 300*	**Logistic regression** *RR (97.5%CI)*
Death or moderate-or-severe neurodevelopmental disability, n (%)	110 (46.0)	115 (46.7)	0.95 (0.76-1.19)
- missing, n (%)	62 (20.6)	54 (18.0)	

eTable 9: Comparison of sites with high follow up rates (≥90 vs low follow up rates (<90%) for the co-primary outcome Bayley III/IV cognitive score

Sites with high follow up rate (≥90%)	**Cerebral oximetry** *n = 155*	**Usual care** *n = 165*	**Mixed effects linear regression** *estimate (97.5%CI)*
Bayley III/IV cognitive score, mean (97.5%CI)	91.6 (88.02-95.25)	94.3 (91.27-97.40)	-1.75 (-6.19;2.61)
- missing, n (%)	54 (34.8)	45 (27.3)	
Sites with low follow up rate (<90%)	**Cerebral oximetry** *n = 542*	**Usual care** *n = 576*	**Mixed effects linear regression** *estimate (97.5%CI)*
Bayley III/IV cognitive score, mean (97.5%CI)	93.7 (90.33-96.99)	92.3 (88.99-95.67)	1.06 (-3.25-5.36)
- missing, n (%)	397 (73.2)	412 (71.5)	

eTable 10: Generalized estimation equation (twin sensitivity analysis)

	**Logistic regression** *RR (97.5%CI)*
Death or moderate-or-severe neurodevelopmental disability, n (%)	0.88 (0.71-1.09)
	**Mixed effects linear regression** *Mean difference (97.5%CI)*
Bayley III/IV cognitive score, mean (SD)	1.69 (-4.44 – 7.71)

eTable 11: Proportion of missingness between cerebral oximetry group and experimental group for the two co-primary outcomes

	**Cerebral oximetry** *n = 697*	**Usual care** *n = 741*	**Fisher's exact test** *OR (95%CI)*
Death or moderate-or-severe neurodevelopmental disability is missing, n (%)	77 (11.0)	72 (9.7)	1.15 (0.81-1.65)
Bayley III/IV cognitive mean score is missing, n (%)	451 (64.7)	457 (61.7)	1.14 (0.91-1.42)

eTable 12: Analysis of all PARCA-R non-verbal cognitive scores including extrapolated scores

	**Cerebral oximetry** *n = 623*	**Usual care** *n = 669*	**Logistic regression** *RR (97.5%CI)*
Death or moderate-or-severe neurodevelopmental disability with extrapolated scores, n (%)	294 (47.2%)	323 (48.3%)	0.95 (0.85-1.07)
Extra infants classified, n	3	0	

	**Cerebral oximetry** *n = 697*	**Usual care** *n = 741*	**Wilcoxon rank sum test** *MD (95%HLCI)*
Mean of PARCA-R NVC within time frame, n (mean, SD)	258 (86.4, 21.18)	268 (87.4, 21.26)	0.00 (-4.00-3.00)
Mean of all PARCA-R NVC scores, n (mean, SD)	320 (86.5, 21.26)	337 (87.8, 22.12)	-1.00 (-4.00-2.00)

eTable 13: Death or moderate-or-severe neurodevelopmental disability with PARCA-R non-verbal cognitive extrapolated scores

eTable 14: Follow up times of randomized infants

	**Cerebral oximetry** *n = 697*	**Usual care** *n = 741*
Weight, median corrected age in months (IQR)	24.4 (22.4-25.6)	24.3 (23.1-25.5)
- missing	295 (42.3%)	296 (39.9%)
Height, median corrected age in months (IQR)	24.3 (22.1-25.5)	24.3 (23.2-25.4)
- missing	305 (43.8%)	306 (41.3%)
Head circumference, median corrected age in months (IQR)	24.2 (21.3-25.3)	24.2 (21.8-25.3)
- missing	315 (45.2%)	310 (41.8%)
PARCA-R non-verbal cognitive score, median corrected age (IQR)	25.5 (24.5-27.4)	25.7 (24.4-27.8)
- missing	343 (49.2%)	354 (47.8%)
Bayley III/IV assessment, median corrected age in months (IQR)	24.5 (23.7-25.5)	24.5 (23.8-25.3)
- missing	451 (64.7%)	457 (61.7%)

	**Cerebral oximetry** *n = 697*	**Usual care** *n = 741*
Neuropediatric assessment, median corrected age in months (IQR)	24.17 (23.0-25.3)	24.4 (23.4-25.3)
- missing	581 (83.4%)	613 (82.7%)
Pediatrician/neonatologist assessment, median corrected age in months (IQR)	24.4 (22.7-25.6)	24.3 (23.2-25.6)
- missing	356 (51.1%)	351 (47.4%)
Physiotherapist assessment, median corrected age in months (IQR)	24.0 (20.8-24.8)	24.2 (23.6-25.4)
- missing	604 (86.7%)	639 (86.2%)
Psychologist assessment, median corrected age in months (IQR)	24.5 (23.8-25.2)	24.6 (23.8-25.7)
missing	532 (76.3%)	549 (74.1%)

eTabel 15: Clinical data based on information from health care professional

Seen by	**Cerebral oximetry** *n = 535*	**Usual care** *n = 576*
Neuropediatrician, Pediatrician/neonatologist, Physiotherapist and Psychologist, no. (%)	25 (4.7)	24 (4.2)
Neuropediatrician, Pediatrician/neonatologist, and Physiotherapist, no. (%)	11 (2.1)	13 (2.3)
Neuropediatrician, Pediatrician/neonatologist and Psychologist, no. (%)	17 (3.2)	24 (4.2)
Pediatrician/neonatologist, Physiotherapist and Psychologist, no. (%)	21 (3.9)	28 (4.9)
Neuropediatrician, Physiotherapist and Psychologist, no. (%)	4 (0.7)	4 (0.7)
Neuropediatrician and Pediatrician/neonatologist, no. (%)	12 (2.2)	15 (2.6)
Pediatrician/neonatologist and Physiotherapist, no. (%)	29 (5.4)	29 (5.0)
Pediatrician/neonatologist and Psychologist, no. (%)	66 (12.3)	87 (15.1)
Neuropediatrician and Physiotherapist, no. (%)	2 (0.4)	2 (0.3)
Neuropediatrician and Psychologist, no. (%)	18 (3.4)	17 (3.0)
Pediatrician/neonatologist, no. (%)	160 (29.9)	170 (29.5)
Neuropediatrician, no. (%)	27 (5.0)	29 (5.0)
Physiotherapist, no. (%)	1 (0.2)	2 (0.3)
Psychologist, no. (%)	14 (2.6)	8 (1.4)
None of above reported, no. (%)	128 (23.9)	124 (21.5)

eTable 16: Parental education level based on ISCED classification

	**Control group** *n = 741*	**Experimental group** *n = 697*
Maternal education – (ISCED levels*, n (mean, 95%CI)	373 (4.39, 4.17-4.60)	342 (4.56, 4.35-4.78)
Paternal education – (ISCED levels, n (mean, 95%CI)	362 (4.20, 3.99-4.41)	333 (3.99, 3.78-4.20)

*Distribution of participants according to their highest level of education, as reported in the parental questionnaires and classified using the International Standard Classification of Education (ISCED) system. ISCED levels range from Level 0 (pre-primary education) to Level 8 (doctoral or equivalent level).

eTable 17: Number of randomizations per site

**Country**	**Department**	**Randomized**
Denmark	Department of Neonatology, Copenhagen University Hospital - Rigshospitalet, Copenhagen	106
Spain	Department of Neonatology, La Paz University Hospital, Madrid	78
Spain	Neonatology Department, 12 de Octubre University Hospital, Madrid	73
Czech Republic	The Institute for the Care of Mother and Child, Prague	66
Spain	Department of Neonatology, Hospital Clínic Barcelona, Barcelona	59
Italy	Fondazione IRCCS Ca' Granda Ospedale Maggiore Policlinico Milan, Milano	45
Switzerland	Neonatal and Pediatric Intensive Care Unit, Children's Hospital Lucerne, Lucerne	44
Turkey	Department of Neonatology, Bursa Uludag University Faculty of Medicine, Bursa	44
Greece	Department of Neonatology Ippokrateion General Hospital of Thessaloniki, Thessaloniki	40
United States	Division of Neonatology, Loma Linda University, Loma Linda Children's Hospital	38
Switzerland	Department of Neonatology, University Hospital Zurich, Zurich	37
Poland	Department of Neonatology, Centrum Medyczne "Ujastek" Sp. z o.o., Krakow	37
Turkey	Department of Neonatology, NICU, Ankara City Hospital, Ankara	37
Spain	Department of Neonatology. Hospital Clinico San Carlos - IdISSC, Madrid	36
Norway	Department of Neonatology, Oslo University Hospital, Oslo	36

Poland	Department of Neonatology, Poznan	34
Turkey	Division of Neonatology, Department of Pediatrics, Marmara University Research and Education Hospital, Istanbul	33
Belgium	Department of Development and Regeneration KU Leuven, Leuven	32
Spain	Department of Neonatology, Hospital Sant Joan de Deu, Barcelona	32
United States	Division of Neonatology, University of Utah Hospital, Salt Lake City,	28
Belgium	Service de néonatologie, Clinique CHC Montlégia- Liège Belgium, Liege	24
Switzerland	Clinic of Neonatology, Department Women-Mother-Child, University Hospital Center and University of Lausanne, Vaud	23
Spain	Neonatal Unit, Marqués de Valdecilla University Hospital-IDIVAL, Santander, Cantabria	23
Denmark	Neonatal Intensive Care Unit, Aarhus University Hospital, Aarhus	22
Spain	Department of Neonatology, Puerta del Mar University Hospital, Cádiz	22
Ireland	Infant Centre and Department of Paediatrics and Child Health, University College Cork, Cork	22
Austria	Department of Pediatrics, Medical University of Graz, Graz	21
Denmark	Department of Neonatology, Aalborg University Hospital, Aalborg	21
United Kingdom	Department of Neonatal Medicine, Royal Hospital for Children, Glasgow	21
Poland	Department of Neonatology, Jagiellonian University Hospital, Kraków	20
Belgium	Department of Neonatology, GHdC Charleroi, Charleroi	19
Ireland	Department of Neonatology, The Rotunda Hospital, Dublin	19
Ireland	Department of Neonatology, National Maternity Hospital, Dublin	19
Ireland	The Coombe Hospital, Dublin	18
Turkey	Division of Newborn Medicine, Gazi University Hospital, Ankara	17
Germany	Division of Neonatology and Pediatric Intensive Care Medicine, Center for Pediatrics and Adolescent Medicine, Medical Center – University of Freiburg, Freiburg	16
Greece	Neonatal Intensive Care Unit, “Alexandra” University and State Maternity Hospital, Athens	16
United States	Department of Pediatrics, Division of Newborn Medicine, St. Louis University Hospital, Missouri	16
Italy	Unità Operativa Complessa di Neonatologia, Fondazione Policlinico Universitario A. Gemelli IRCCS, Rome	15
Belgium	Department of Neonatology, AZ St-Jan Bruges, Bruges	14
Greece	NICU, Department of Pediatrics, Patras University Hospital, Patras	13
Turkey	Basaksehir Cam and Sakura City Hospital, Istanbul	13
Denmark	Department of Pediatrics, Odense University Hospital, Odense	12

Switzerland	Neonatal Unit, Division of Neonatology and Pediatric Intensive Care, Children's University Hospital of Geneva, Geneva	11
Italy	SC Neonatologia, Osp. S.Anna - Città della Salute e della Scienza di Torino, Turin	11
Turkey	Department of Neonatology, Kanuni Sultan Suleyman Training and Research Hospital, Küçükçekmece/İstanbul, Istanbul	11
United Kingdom	Neonatology Department, University Hospital Wishaw, Wishaw	11
Czech Republic	Department of Neonatology, University Hospital Motol, Prague	10
Greece	Department of Neonatology & NICU, University Hospital of Heraklion, Crete	10
Poland	Department of Neonatology and Neonatal Intensive Care Medical University of Warsaw, Warsaw	9
Poland	Neonatal Unit, Specialist Hospital No.2, Bytom	8
United States	Division of Pediatrics - Neonatal-Perinatal, UT Southwestern, Texas	6
India	Department of Neonatology, St Johns Medical College Hospital, Bangalore	5
Poland	Department of Neonatology, Collegium Medicum in Bydgoszcz Nicolaus Copernicus University, Torun	3
Spain	Neonatology Division, Miguel Servet University Hospital, Zaragoza	1
Poland	Department of Neonatology, Medical University in Wroclaw, Wroclaw	1

Supplementary Figures

eFigure 1: Prioritisation of data for the co-primary outcome moderate-or-severe neurodevelopmental disability*

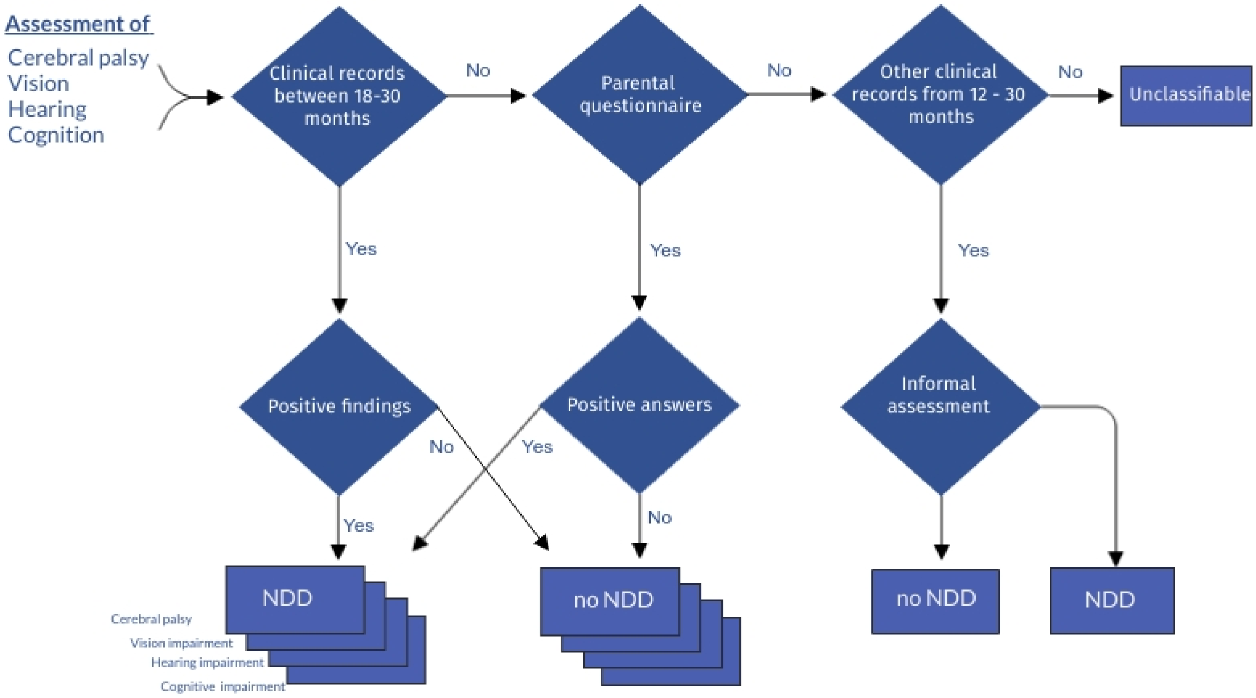

*NDD = moderate-or-severe neurodevelopmental disability = positive findings for at least one of the four components or NDD based on an informal blinded assessment of all available information.

No NDD = no moderate-or-severe neurodevelopmental disability = no positive findings in any of the four components and no NDD based on an informal blinded assessment of all available information.

Unclassifiable = if one or more components were unknown, none were positive, and there was no informal assessment.

eFigure 2a: Random effect meta-analysis for co-primary outcome moderate-or-severe neurodevelopmental disability

The sites are listed in random order. Sites with no primary outcome events in the randomized infants are not represented. The size of the squares represents number of randomized infants and primary outcome events; the larger the square, the more randomized infants and events. The site intra-cluster correlation analysis showed negligible intra-cluster correlation coefficient ~ 0.40 for the dichotomous outcome. However, this is higher than the one seen in the 36-week postmenstrual age analysis.

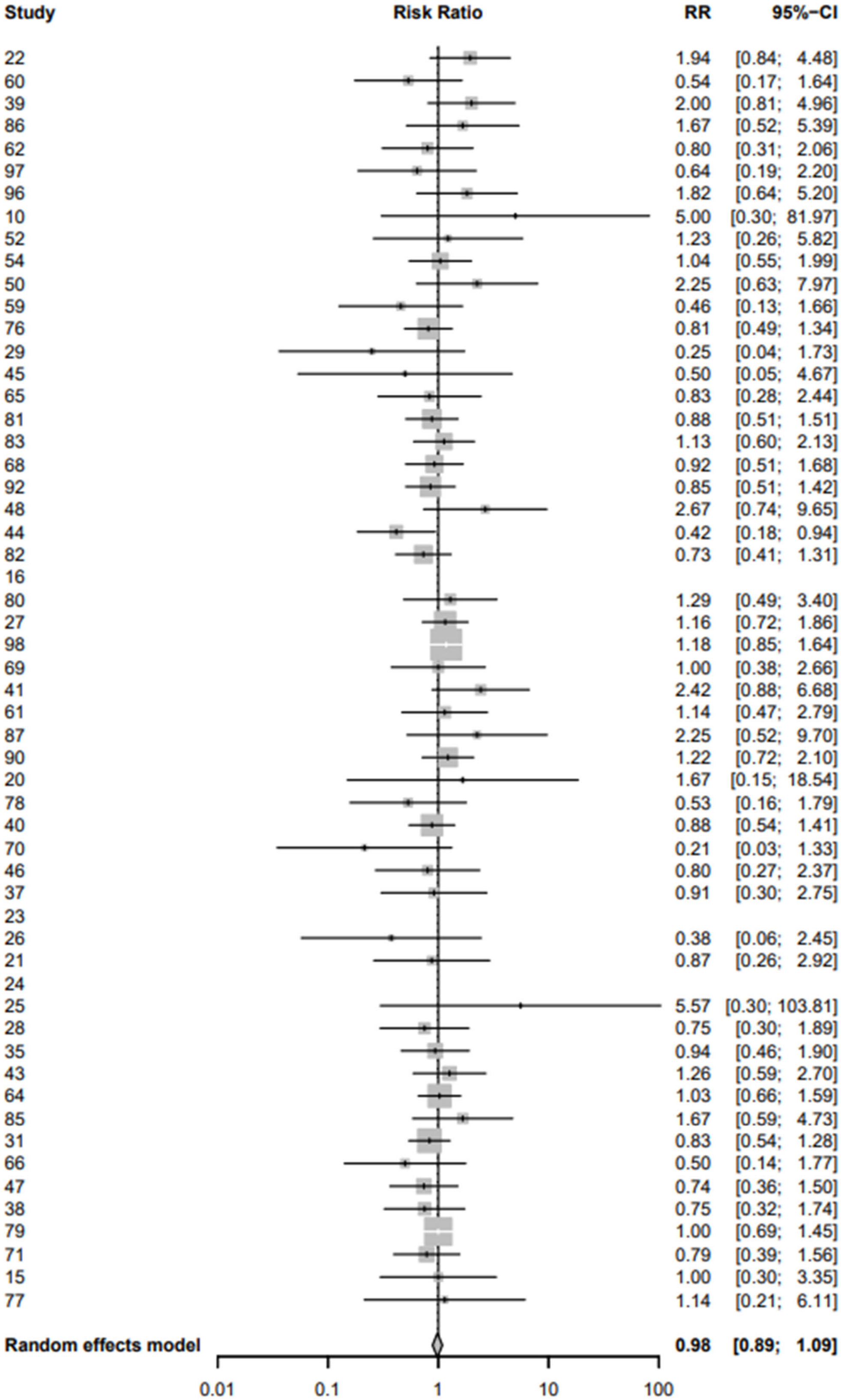
Study
Risk Ratio
RR
95%−CI
22 1.94 [0.84; 4.48]
60 0.54 [0.17; 1.64]
39 2.00 [0.81; 4.96]
86 1.67 [0.52; 5.39]
62 0.80 [0.31; 2.06]
97 0.64 [0.19; 2.20]
96 1.82 [0.64; 5.20]
10 5.00 [0.30; 81.97]
52 1.23 [0.26; 5.82]
54 1.04 [0.55; 1.99]
50 2.25 [0.63; 7.97]
59 0.46 [0.13; 1.66]
76 0.81 [0.49; 1.34]
29 0.25 [0.04; 1.73]
45 0.50 [0.05; 4.67]
65 0.83 [0.28; 2.44]
81 0.88 [0.51; 1.51]
83 1.13 [0.60; 2.13]
68 0.92 [0.51; 1.68]
92 0.85 [0.51; 1.42]
48 2.67 [0.74; 9.65]
44 0.42 [0.18; 0.94]
82 0.73 [0.41; 1.31]
16
80 1.29 [0.49; 3.40]
27 1.16 [0.72; 1.86]
98 1.18 [0.85; 1.64]
69 1.00 [0.38; 2.66]
41 2.42 [0.88; 6.68]
61 1.14 [0.47; 2.79]
87 2.25 [0.52; 9.70]
90 1.22 [0.72; 2.10]
20 1.67 [0.15; 18.54]
78 0.53 [0.16; 1.79]
40 0.88 [0.54; 1.41]
70 0.21 [0.03; 1.33]
46 0.80 [0.27; 2.37]
37 0.91 [0.30; 2.75]
23
26 0.38 [0.06; 2.45]
21 0.87 [0.26; 2.92]
24
25 5.57 [0.30; 103.81]
28 0.75 [0.30; 1.89]
35 0.94 [0.46; 1.90]
43 1.26 [0.59; 2.70]
64 1.03 [0.66; 1.59]
85 1.67 [0.59; 4.73]
31 0.83 [0.54; 1.28]
66 0.50 [0.14; 1.77]
47 0.74 [0.36; 1.50]
38 0.75 [0.32; 1.74]
79 1.00 [0.69; 1.45]
71 0.79 [0.39; 1.56]
15 1.00 [0.30; 3.35]
77 1.14 [0.21; 6.11]
Random effects model 0.98 [0.89; 1.09]
0.01
0.1
1
10
100

eFigure 2b: Random effect meta-analysis for co-primary outcome Bayley III/IV cognitive score

The sites are listed in random order. Sites with no primary outcome events in the randomized infants are not represented. Sites without confidence intervals only have one participant with data per group. The size of the squares represents number of randomized infants and primary outcome events; the larger the square, the more randomized infants and events. The site intra-cluster correlation analysis showed negligible intra-cluster correlation coefficient 0.56 for the continuous outcome.

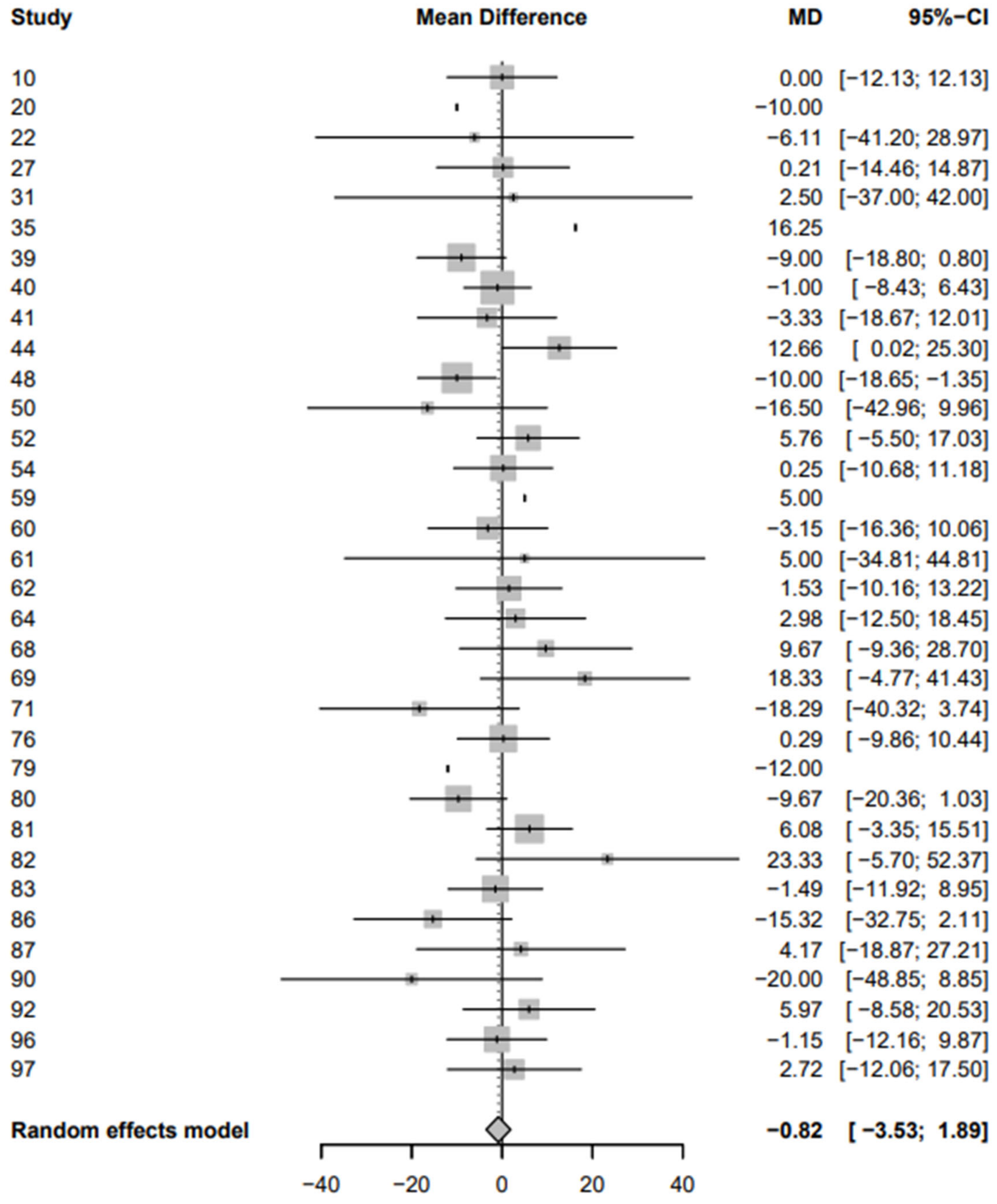

eFigure 3: Extract from the electronic case report form for 2-year clinical data

Followup (0/45)

Title: Followup

Instructions:

2 year follow-up form

Code	Question	Response	
F00	Is the participant lost to follow-up?	○ Yes ○ No	[info]
F00a	If the participant was lost to follow up please state why	○ No consent to use data<br>○ Clinical follow-up in hospital with no access to data<br>○ The family moved away<br>○ Other (please elaborate)<br>○ Unknown	
F00b	Please elaborate	[ ]	
F01	Did the baby die after 36 weeks PMA?	○ Yes ○ No ○ Unknown	
F01a	Date of death	[ ]	[info]
F01b	Cause of death	[ ]	[info]
F02	Are there relevant health care records, such as formal assessments of vison, hearing, psychomotor, neurodevelopment, from corrected age 18-30 months available?	○ Yes ○ No	
F03	Based on the data available what would your informal clinical assessment be of the child's development?	○ No or mild neurodevelopmental disability<br>○ Moderate or severe neurodevelopmental disability	

If the child had a medical assessment between 18-30 months of age, please fill out as much data as possible

Code	Measure	Value	Unit	Code		Date	
F04	Weight	[ ]	(kg)	F04a	Date of measure	[ ]	☐ Not Available
F05	Height	[ ]	(cm)	F05a	Date of measure	[ ]	☐ Not Available
F06	Head circumference	[ ]	(cm)	F06a	Date of measure	[ ]	☐ Not Available

Code	Question	Response	
F07	Has the child been diagnosed with cerebral palsy?	○ Yes ○ No ○ Unknown	[info]
F07a	Please score Global Motor Function Classification System	[ ]	[info]
F07b	Could the child walk independently at two years of age?	○ Yes ○ No ○ Unknown	
F08	Has the child been diagnosed with a visual impairment?	○ Yes ○ No ○ Unknown	[info]
F09	Has the child been diagnosed with a hearing impairment?	○ Yes ○ No ○ Unknown	[info]
F10	Was retinopathy of prematurity grade 3 or more diagnosed after 36 weeks postmenstrual age?	○ Yes ○ No ○ Unknown	
F11	Was the baby treated for retinopathy of prematurity, any grade, after 36 weeks of postmenstrual age?	○ Yes ○ No ○ Unknown	
F12	Has the child been diagnosed with any chronic health problems?	○ Yes ○ No ○ Unknown	[info]

F12a	Please state problems	☐ Upper airways<br>☐ Lungs<br>☐ Heart<br>☐ Gastro-intestinal<br>☐ Urinary<br>☐ Skin<br>☐ Orthopaedic/other	
F13	Has the child received any daily medication for the last two months?	◯ Yes ◯ No ◯ Unknown [info]	
F13a	Please elaborate (antiepilectica, beta 2 agonists etc)		
F14	Has the child been assessed with a Bayley-III or Bayley-IV?	◯ Yes ◯ No ◯ Unknown	
F14a	Which type of Bayley assessment test has been used?	◯ Bayley-III ◯ Bayley-IV	
F14b	Cognitive composite score		
F14c	Date of test		
F14d	Is this score corrected for prematurity?	◯ Yes ◯ No	
F15	Has the child been assessed with another standardised neurodevelopmental test?	◯ Yes ◯ No ◯ Unknown	*If more than one test has been performed please prioritize in this order: Bayley II, Griffiths III, Gessell, ASQ, Denver Developmental Screening, Peabody, any other*
F15a	Name of test		F15b Score of test
F15c	Date of test		
F15d	Is this score below the normal range?	◯ Yes ◯ No	*As this test is used in your clinical practice, would the child's score be categorized as subnormal (for example, below -2 standard deviations of the norm)?*
F16	Based on the data available what would your informal clinical assessment be of the child's development?	◯ No or mild neurodevelopmental disability<br>◯ Moderate or severe neurodevelopmental disability	

The information above is based on assessments from (check one or more) and last date of assessment

F17	☐ Neuropediatrician	F17a	Date of last assessment
F18	☐ Pediatrician/neonatologist	F18a	Date of last assessment
F19	☐ Physiotherapist	F19a	Date of last assessment
F20	☐ Psychologist	F20a	Date of last assessment

eFigure 4: Parental questionnaire used for the SafeBoosC-III follow up study

The SafeBoosC-III two year follow up: parental questionnaire

No one knows your child like you do. That is why we would like you to tell us how your child is doing now that they are 2 years of age. Only a study participant number will be attached to the data. No name or other information which could lead back to your family is documented for the study. We expect that the questionnaire will take approximately 15 minutes to fill out.

Date: ____

Study ID: ____

Childs play

We are interested in finding out about your child's play as this will give us an idea of how his or her problem-solving skills are developing. Please tell us whether your child can do each of the play activities below. If you have seen your child do the activity (or something similar), then tick the box under "Yes". If you know that your child would not be able to do it, then tick the box under "No". If you are not sure, then tick the box for "Don't know." If you don't know, you may like to try out some of the activities with your child. Please answer all the questions as best you can. There are 34 questions in this part of the questionnaire.

Please remember that all children develop differently and there are large differences in what children can do at this age. Some of these activities may be easy for your child and others may be difficult. The activities listed are for children up to 4 years of age, so most children will not be able to do all of them yet.

1. Does your child copy things you do such as cuddling a teddy? (Try it out if not sure by cuddling a teddy and then giving it to your child. Say: Now you cuddle teddy)

☐ Yes

☐ No

☐ Don't know

2. When you hide a toy in full view of your child, will s/he look for it and find it? (Try this out by covering a small toy with a cloth or a cup and seeing if s/he uncovers the toy)

☐ Yes

☐ No

☐ Don't know

3. Can your child put a simple piece, such as a square or an animal, into the correct place in a puzzle board?

☐ Yes

☐ No

☐ Don't know

4. Some toys have several holes or openings with different shapes, such as a circle, triangle, and star. Could your child put the shapes into the right openings?

☐ Yes

☐ No

☐ Don't know

5. Can your child stack two small blocks or toys on top of each other?

☐ Yes

☐ No

☐ Don't know

6. Can your child put together, by him/herself, a puzzle or something similar where the pieces fit together?

☐ Yes

☐ No

☐ Don't know

7. If so, can s/he do this for a puzzle with ten or more pieces?

☐ Yes

☐ No

☐ Don't know

8. Can your child mark on a piece of paper using the tip of a crayon, pencil, or chalk?

- ☐ Yes
- ☐ No
- ☐ Don't know

9. Can your child draw a more or less straight line on paper?

- ☐ Yes
- ☐ No
- ☐ Don't know

10. Does your child turn, or try to turn, pages of a book one at a time?

- ☐ Yes
- ☐ No
- ☐ Don't know

11. Does your child ever pretend that one object, such as a block, is another object, such as a car or a telephone?

- ☐ Yes
- ☐ No
- ☐ Don't know

12. Can your child stack three small blocks or toys on top of each other by him/herself?

- ☐ Yes
- ☐ No
- ☐ Don't know

13. Does your child ever pretend to do things? For example, riding a horse or making a cup of tea?

- ☐ Yes
- ☐ No
- ☐ Don't know

14. Can your child push a car along the floor with the wheels on the floor?

- [ ] Yes
- [ ] No
- [ ] Don't know

15. Does your child look with interest at pictures in a book?

- [ ] Yes
- [ ] No
- [ ] Don't know

16. Does your child point to pictures in a book?

- [ ] Yes
- [ ] No
- [ ] Don't know

17. Does your child try to copy things you do, such as stirring with a spoon in a cup?

- [ ] Yes
- [ ] No
- [ ] Don't know

18. Can your child stack seven small blocks or toys on top of each other by him/herself?

- [ ] Yes
- [ ] No
- [ ] Don't know

19. Does your child point or show where people or objects are when you ask: "Where is the light?" "Where is Daddy?" or "Where is Teddy?"

- [ ] Yes
- [ ] No
- [ ] Don't know

20. Does your child ever pretend that two dolls are playing together, or are talking to each other, or one is feeding the other?

☐ Yes

☐ No

☐ Don't know

21. Does your child ever play pretend games with another child, pretending to be someone else, such as a mummy, daddy, policeman, or nurse?

☐ Yes

☐ No

☐ Don't know

22. Does your child ever play any game with another child that involves taking turns?

☐ Yes

☐ No

☐ Don't know

23. Does your child ever copy some action shortly (within a few minutes) after s/he has seen it?

☐ Yes

☐ No

☐ Don't know

24. Can your child fetch something, such as a toy, from another room by him/herself when you ask?

☐ Yes

☐ No

☐ Don't know

25. Does your child know where some things belong, such as, that his/her toys belong in a box?

☐ Yes

☐ No

☐ Don't know

26. Does your child ever save or put to one side a biscuit (or snack) for later, on his/her own?

☐ Yes

☐ No

☐ Don't know

27. Have you ever seen your child get together three or more toys before beginning to play with them?

☐ Yes

☐ No

☐ Don't know

28. Have you ever seen your child sort things (blocks, other toys) into groups or piles that go together on his/her own?

☐ Yes

☐ No

☐ Don't know

29. If your child wants something out of reach, does s/he go and find a chair or box to stand on?

☐ Yes

☐ No

☐ Don't know

30. When your child uses or plays with a telephone, does s/he speak into the mouthpiece not the earpiece?

- [ ] Yes
- [ ] No
- [ ] Don't know

31. When your child drinks from a cup, is s/he careful about putting it down, trying not to spill it?

- [ ] Yes
- [ ] No
- [ ] Don't know

32. Does your child try to turn doorknobs, twist tops, or screw lids on or off jars?

- [ ] Yes
- [ ] No
- [ ] Don't know

33. Does your child recognise him/her self when looking in the mirror?

- [ ] Yes
- [ ] No
- [ ] Don't know

34. Does your child ever use his/her index (first) finger to point to show an interest in something?

- [ ] Yes
- [ ] No
- [ ] Don't know

Childs health and development

In this section you will find general questions on your child's general health and development and a question regarding parental education. Please answer these to the best of your ability.

1. Has a doctor said that your child has cerebral palsy?

☐ Yes

☐ No

☐ Don't know

2. Is your child able to walk on his or her own, without any support?

☐ Yes

☐ No

☐ Don't know

3. Is your child able to sit *on the floor* on his or her own, without any support?

☐ Yes

☐ No

☐ Don't know

4. Does your child have a hearing impairment?

☐ Yes

☐ No

☐ Don't know

Only answer 4.a and 4.b if your answer to 4 is YES.
Please continue otherwise.

4.a

Does your child wear hearing aids?

☐ Yes

☐ No

☐ Don't know

4.b

Has your child had cochlear implants?

☐ Yes

☐ No

☐ Don't know

5. Does your child have any visual problems?

☐ Yes

☐ No

☐ Don't know

Only answer 5.a, 5.b, 5.c if your answer to 5 is YES. Please continue otherwise.

5.a

Does your child wear glasses?

☐ Yes

☐ No

☐ Don't know

5.b

Does your child have a squint? (the eyes look in different directions)

☐ Yes

☐ No

☐ Don't know

5.c

Is your child blind on one or two eyes or has poor vision even with glasses?

☐ Yes

☐ No

☐ Don't know

6. Does your child have a chronic health problem that affects daily life on most days? (such as breathing problems, diarrhea, seizures)

☐ Yes

☐ No

☐ Don't know

7. Does your child get any daily medication during the last two months and why? (vitamins are not counted as medication)

☐ Yes

☐ No

☐ Don't know

If yes, please state why:____

8. Has your child been admitted to a hospital (staying overnight) since you got her/him home the first time?

Please check all the boxes that apply in question 8

- ☐ No
- ☐ Yes, more than once
- ☐ Yes, and he/she required intensive care

If yes, please state the main reason:____

9. Is your child happy and thriving?

Only answer 9. b if your answer to 4 is NO. Please continue otherwise.

- ☐ Yes
- ☐ No
- ☐ Don't know

9.a does the problem relate to (check as many as apply)

- ☐ Eating
- ☐ Sleeping
- ☐ Playing independently
- ☐ Mood
- ☐ Social interactions
- ☐ Development

10. Do you have any particular worries about your child?

- ☐ Yes
- ☐ No
- ☐ Don't know

If yes, please elaborate:____

____

____

11. What is the highest level of education the mother of the child has completed?

- ☐ Early childhood education ('less than primary' for educational attainment)
- ☐ Primary education
- ☐ Lower secondary education
- ☐ Upper secondary education
- ☐ Post-secondary non-tertiary education
- ☐ Short-cycle tertiary education
- ☐ Bachelor's or equivalent level
- ☐ Master's or equivalent level
- ☐ Doctoral or equivalent level

12. What is the highest level of education the father of the child has completed?

- ☐ Early childhood education ('less than primary' for educational attainment)
- ☐ Primary education
- ☐ Lower secondary education
- ☐ Upper secondary education
- ☐ Post-secondary non-tertiary education
- ☐ Short-cycle tertiary education
- ☐ Bachelor's or equivalent level
- ☐ Master's or equivalent level
- ☐ Doctoral or equivalent level

Thank you for completing this questionnaire.
Your contribution to the SafeBoosC-III follow up study is truly appreciated.

eReferences

1. Olsen MH, Hansen ML, Safi S, et al. Central data monitoring in the multicentre randomised SafeBoosC-III trial - a pragmatic approach. BMC Med Res Methodol 2021;21(1):160. DOI: 10.1186/s12874-021-01344-4.
2. Squires J, Bricker DD, Twombly E, Potter L. Ages & Stages Questionnaires: A Parent-Completed, Child-Monitoring System (3rd ed.). 2009.
3. Iceta A, Yoldi-Petri M. Psychomotor development of the child and its evaluation in primary care. Anales del sistema sanitario de Navarra 2002;25 Suppl 2:35-43. DOI: 10.23938/ASSN.0829.
4. Green E, Stroud L, O'Connell R, et al. Griffiths Scales of Child Development 3rd Edition; Part 2: Administration and scoring2016.
5. Gauthier SM, Bauer CR, Messinger DS, Closius JM. The Bayley Scales of Infant Development. II: Where to start? J Dev Behav Pediatr 1999;20(2):75-9. (In eng). DOI: 10.1097/00004703-199904000-00001.
6. Frankenburg WK, Dodds J, Archer P, Shapiro H, Bresnick B. The Denver II: a major revision and restandardization of the Denver Developmental Screening Test. Pediatrics 1992;89(1):91-7. (In eng).
7. Elbaum B, Gattamorta K, Penfield R. Evaluation of the Battelle Developmental Inventory, 2nd Edition, Screening Test for Use in States' Child Outcomes Measurement Systems Under the Individuals With Disabilities Education Act. Journal of Early Intervention - J EARLY INTERVENTION 2010;32:255-273. DOI: 10.1177/1053815110384723.
8. Brunet O, Lézine In, Josse D. Brunet-Lézine révisé : échelle de développement psychomoteur de la première enfance : manuel BLR-C. rév ed. Issy-Les-Moulineaux (France): Etablissements d'Applications Psychotechniques, 1997.
9. Bellman M LS, Aukett A. Schedule of Growing Skills II: Users Guide. 1996. (https://support.gl-education.com/media/2407/sgs-ii-user-guide.pdf).

10. Ames LB, Gesell Institute of Child D. The Gesell Institute's child from one to six : evaluating the behavior of the preschool child. 1st ed. New York: Harper & Row, 1979.

11. Alpern GD. Developmental Profile 3 (DP-3) Manual. 2023. (Alpern, G. (2023). Developmental Profile 3 (DP-3) Manual. Western Psychological Services. Retrieved from https://reachoutandread.org/wp-content/uploads/2023/06/DP-3-Manual.pdf) (https://reachoutandread.org/wp-content/uploads/2023/06/DP-3-Manual.pdf).

12. Hansen ML, Pellicer A, Hyttel-Sorensen S, et al. Cerebral Oximetry Monitoring in Extremely Preterm Infants. N Engl J Med 2023;388(16):1501-1511. DOI: 10.1056/NEJMoa2207554.
