# Supplementary material for: Cerebral Oximetry in Extremely Preterm Infants: 2-Year Follow-Up of the SafeBoosC-III Randomized Clinical Trial
Source: JAMA Pediatr. 2026 Apr 20;180(6):619–27. doi: 10.1001/jamapediatrics.2026.1066 (PMC13097032; doi:10.1001/jamapediatrics.2026.1066)
Supplement: Supplement 4. — Nonauthor Collaborators. SafeBoosC-III Follow-Up Collaborator Group [file jamapediatr-e261066-s004.pdf]

Supplemental Online Content: Nonauthor Collaborators
*Indicates required information. Only first name, last name, and suffix will appear in PubMed.

*Group Name(s): SafeBoosC-III Follow-Up Collaborator Group							
***First Name and Middle Initial(s)**	***Last Name**	***Suffix (eg, Jr, III)**	Academic Degrees	Institution	Location (city, state/province, country)	Role or Contribution, eg, chair, principal investigator	Group (if more than 1 Group listed in the byline) and/or Subgroup (eg, Steering Committee)
Elisabeth	Pichler-Stachl			University Hospital Graz	Graz, Austria		
Els	Ortibus			University Hospital Leuven	Leuven, Belgium		
Nancy	Laval			Clinique CHC Montlegia	Liege, Belgium		
Anna	Oostra			AZ St. Jan Bruges	Bruges, Belgium		
Marie-Julie	Debuf			Grand Hospital de Charleroi	Charleroi, Belgium		
Katarina	Ticha			University Hospital Motol	Prague, Czech Republic		
Zuzana	Matějková			The Institute for the Care of Mother a	Prague, Czech Republic		
Alexander	Scheid			Aalborg University Hospital	Aalborg, Denmark		
Aikaterini	Nourloglou			University of Patras General Hospital	Patras, Greece		
Vasiliki	Kourou			Ippokrateion General Hospital of The	Thessaloniki, Greece		
Nicole Hilda	Anagnostatou			University Hospital of Heraklion	Heraklion, Greece		
Anjali	Raj			St. John’s Medical College Hospital	Karnataka, India		
Iyshwarya	Stapleton			INFANT Research Centre, University (	Cork, Ireland		
Jsun	Wong			Coombe Woman and Infant Universit	Dublin, Ireland		
Jyothsna	Purna			National Maternity Hospital, Holles S	Dublin, Ireland		
Camilla	Fontana			Fondazione IRCCS Ca’ Granda Osped	Milan, Italy		
Francesca	Serrao			Fondazione Policlinico Universitario A	Roma, Italy		
Anna Zoraide	Patria			Struttura Complessa di Neonatologia,	Turin, Italy		
Tone	Nordvik			Oslo University Hospital	Oslo, Norway		
Roksana	Malak			Poznan University of Medical Science	Poznan, Poland		
Elzbieta	Rafinska-Wazny			Centrum Medyczne "Ujastek" Sp. z o.	Krakow, Poland		
Agnieszka	Ochoda-Mazur			Jagiellonian University Medical Colleg	Krakow, Poland		
Justyna	Fiałkowska			Medical University of Warsaw	Warsaw, Poland		
Kasia	Szczepanska			Specialist Hospital No. 2	Bytom, Poland		
Paulina	Gaweł			Wroclaw Medical University	Wroclaw, Poland		
Iga	Rupniak			Collegium Medicum in Bydgoszcz Nic	Bydgoszcz, Poland		
Malaika Cordeiro	Alcaine			La Paz University Hospital	Madrid, Spain		
Marta	Teresa-Palacio			Hospital Clinic Barcelona	Barcelona, Spain		
Maria Palomares	Eraso			12 de Octubre University Hospital	Madrid, Spain		

Supplemental Online Content: Nonauthor Collaborators

*Indicates required information. Only first name, last name, and suffix will appear in PubMed.

***First Name and Middle Initial(s)**	***Last Name**	***Suffix (eg, Jr, III)**	Academic Degrees	Institution	Location (city, state/province, country)	Role or Contribution, eg, chair, principal investigator	Group (if more than 1 Group listed in the byline) and/or Subgroup (eg, Steering Committee)
Thais Agut	Quijano			Hospital Sant Joan De Deu	Barcelona, Spain		
Isabel Benavente	Fernández			Puerta del Mar University Hospital	Cadiz, Spain		
Isabel Cuellar	Flores			Hospital Clinico San Carlos	Madrid, Spain		
Rosa Ayesa	Arriola			Marques de Valdecilla University Hos	Santander, Spain		
Pilar	Abenia			Miguel Servet University Hospital	Zaragoza, Spain		
Claudia	Knöpfli			University Hospital Zürich	Zürich, Switzerland		
Barbara	Imboden			Children's Hospital Lucerne	Lucerne, Switzerland		
Cristina Borradori	Tolsa			Children's University Hospital of Gen	Geneva, Switzerland		
Myriam Bickle	Graz			University Hospital Centre of Lausanr	Lausanne, Switzerland		
Sebnem	Soysal			Gazi University Hospital	Ankara, Turkey		
Sinem Gulcan	Kersin			Marmara University Research and Ed	Istanbul, Turkey		
Mustafa	Bostanci			Bursa Uludag University Hospital	Bursa, Turkey		
Halime Sema Can	Buker			Kanuni Sultan Süleyman Training and	Istanbul, Turkey		
Gülsüm	Kadıoğlu			Ankara City Hospital	Ankara, Turkey		
Halime Sema Can	Buker			Basaksehir Cam and Sakura City Hosp	Istanbul, Turkey		
Ani	Majeed			University Hospital Wishaw	Wishaw, United Kingdom		
Trisha	Marchant			University of Utah Hospital	Salt Lake City, USA		
Katelyn	Hoffer			UT Southwestern Medical Center	Dallas, USA		
Amanda	Duncan			St. Louis Children's Hospital	St Louis, USA		
