# Supplementary material for: Cerebral Oximetry in Extremely Preterm Infants: 2-Year Follow-Up of the SafeBoosC-III Randomized Clinical Trial
Source: JAMA Pediatr. 2026 Apr 20;180(6):619–27. doi: 10.1001/jamapediatrics.2026.1066 (PMC13097032; doi:10.1001/jamapediatrics.2026.1066)
Supplement: Supplement 5. — Data Sharing Statement. [file jamapediatr-e261066-s005.pdf]

Data Sharing Statement

Rasmussen. Cerebral Oximetry in Extremely Preterm Infants. *JAMA Pediatr*. Published April 20, 2026. doi:10.1001/jamapediatrics.2026.1066

Data
Additional Information: NCT05134116
Data available: Yes
Data types: Deidentified participant data
How to access data: Per request from the corresponding author and by approval from the steering committee
When available: With publication

Supporting Documents
Document types: None

Additional Information
Who can access the data: Reseachers with a request approved by the steering committee
Types of analyses: Specified purpose
Mechanisms of data availability: Per request from the corresponding author and by approval from the steering committee
